# Combating Antibacterial Resistance: The Integrative Role of Artificial Intelligence in Bio-Based Product Development

**DOI:** 10.3390/antibiotics15050478

**Published:** 2026-05-08

**Authors:** Renuka Gudepu, Swapna Sirikonda, Ravinaik Banoth, Praveen Kumar Annagowni, Swati Dahariya, Aditya Velidandi

**Affiliations:** 1 Department of Microbiology, Pingle Government College for Women (A), Warangal 506370, Telangana, India; renumanduva@gmail.com; 2 Department of Pharmaceutics, School of Pharmacy, Anurag University, Hyderabad 500088, Telangana, India; swapnapharmacy@anurag.edu.in; 3 Department of Mechanical Engineering, St. Martin’s Engineering College, Secunderabad 500100, Telangana, India; drravinaikme@smec.co.in; 4 Department of Pharmaceuticals Sciences, Jawaharlal Nehru Technology University, Anantapur 515002, Andhra Pradesh, India; praveengoudag@gmail.com; 5 Department of Biochemistry, School of Life Sciences, University of Hyderabad, Hyderabad 500019, Telangana, India; swati@uohyd.ac.in; 6 Department of Biotechnology, Vaagdevi Degree and P.G. College, Warangal 506001, Telangana, India

**Keywords:** antibacterial resistance, artificial intelligence, biosynthetic gene clusters, deep learning, genome mining, machine learning, bio-based products

## Abstract

The escalating crisis of antimicrobial resistance claims nearly 5 million lives annually. Resistant infections now account for 4.95 million deaths worldwide and economic losses projected to reach $300 billion by 2030. Despite this urgent threat, traditional antibiotic discovery has declined precipitously. New chemical entity approvals have fallen by over 50%, while existing therapeutics are rapidly rendered obsolete by sophisticated bacterial resistance mechanisms including extended-spectrum β-lactamases, carbapenemases, and multidrug efflux pumps. Bio-based products have historically provided humanity’s most transformative antibiotics, yet conventional discovery pipelines face insurmountable bottlenecks. A total of 99.9% of environmental microbes remain unculturable. Biosynthetic gene clusters are predominantly silent under laboratory conditions, and dereplication efforts achieve only 2 to 5% annotation rates. This review presents a comprehensive examination of how artificial intelligence (AI) is revolutionizing bio-based product-based antibacterial discovery. We analyze AI-driven genome mining tools that have identified over 170,000 biosynthetic gene clusters across bacterial genomes, deep learning architectures achieving 88.5% bioactivity prediction accuracy, and generative models delivering experimental hit rates exceeding 50%—representing 50- to 90-fold improvements over traditional screening. Through validated case studies spanning in silico prediction to in vivo efficacy, we demonstrate that AI integration is not merely accelerating discovery but fundamentally transforming our capacity to access nature’s previously inaccessible chemical diversity in the fight against antimicrobial resistance.

## 1. Introduction: The Current State of Antibacterial Resistance

Antibacterial resistance, also known as antimicrobial resistance (AMR), has emerged as one of the most pressing global health crises of the 21st century, threatening decades of progress in modern medicine and public health. The escalating prevalence of drug-resistant bacterial infections, coupled with their devastating economic consequences, demands urgent and coordinated international action. This introduction provides a comprehensive overview of the current state of antibacterial resistance, examining global and regional prevalence patterns, mortality burdens, and the substantial financial costs that AMR imposes on healthcare systems and economies worldwide.

The current state of antibacterial resistance represents a multifaceted crisis characterized by escalating prevalence rates, mounting mortality burdens, and devastating economic consequences across all world regions (Table 1). The data presented demonstrates that AMR is not merely a clinical challenge but a fundamental threat to global health security and economic stability. With resistance rates continuing to rise, mortality approaching 5 million deaths annually, and economic costs projected to reach $300 billion by 2030, the urgency for innovative solutions has never been greater. The integration of artificial intelligence (AI) with bio-based product development represents a promising frontier in the fight against AMR, offering the potential to accelerate the discovery and optimization of novel antibacterial agents capable of overcoming existing resistance mechanisms. As this review will demonstrate, the convergence of computational intelligence (AI) and nature’s chemical diversity may provide the transformative approaches necessary to address this escalating global health emergency.

## 2. Limitations of Existing Antibacterial Therapeutics

The remarkable success of antibacterial agents in transforming infectious disease treatment has been progressively undermined by the emergence and dissemination of AMR. Despite the availability of multiple antibiotic classes, contemporary clinical practice faces mounting challenges as bacteria evolve sophisticated mechanisms to evade therapeutic interventions.

### 2.1. Molecular Mechanisms of Antibacterial Resistance

Bacteria have evolved diverse and sophisticated mechanisms to resist antibacterial agents, rendering many once-effective therapeutics increasingly ineffective. These resistance mechanisms can be broadly categorized into four principal strategies: enzymatic degradation or modification of antibiotics, alteration in drug targets, reduction in intracellular antibiotic concentration through efflux pumps or decreased permeability, and metabolic bypass of inhibited pathways [12,13,14]. Understanding these mechanisms at the molecular level is essential for developing strategies to overcome resistance and for rational design of next-generation therapeutics (Table 2).

### 2.2. Class-Specific Limitations of Major Antibiotic Classes

Each major antibiotic class faces distinct limitations arising from specific resistance mechanisms, pharmacological constraints, and clinical challenges. Understanding these class-specific vulnerabilities is essential for rational antibiotic selection and for identifying gaps in the current therapeutic arsenal.

#### 2.2.1. β-Lactam Antibiotics

β-Lactam antibiotics, including penicillins, cephalosporins, and carbapenems, remain among the most widely prescribed antibacterial agents. However, their efficacy has been progressively eroded by multiple resistance mechanisms.

- Penicillins: Resistance to penicillin emerged remarkably rapidly, with *S. aureus* resistance reported within 4 years of penicillin’s market introduction [16]. The primary resistance mechanism involves β-lactamase production (encoded by blaZ) and the acquisition of altered PBPs (PBP2a) in MRSA [16]. Current MRSA prevalence rates demonstrate the extent of this problem, with 32.2% resistance in China and historical mortality rates of 82% among infected patients in the early 1940s, rising to 98% for those aged above 50 years [6,16].
- Cephalosporins: 3GCs face widespread resistance mediated by ESBLs. In China, 3GCREC prevalence reached 54.2% [6]. Across multiple WHO regions, over 50% of *E. coli* isolates demonstrate resistance to 3GCs [11]. In Germany, resistance to 3GCs in Gram-negative pathogens ranges from moderate to high (5 to 25%), with *P. aeruginosa* showing 10.1% resistance [9].
- Carbapenems: Carbapenems were developed as ‘last-resort’ β-lactams to overcome ESBL-mediated resistance, but carbapenemase-producing organisms have emerged as critical threats. Carbapenem-resistant *A. baumannii* prevalence in China reached 56.1% [6]. In Greece, meropenem resistance reaches 75.00% in *K. pneumoniae* and 98.00% in *Acinetobacter* spp., representing near-complete therapeutic failure [8]. Germany reports carbapenem resistance in Gram-negative pathogens at low-to-moderate levels (<9%), but *P. aeruginosa* demonstrates 17.0% carbapenem resistance [9]. *K. pneumoniae* shows over 50% resistance to carbapenems across multiple WHO regions [11].
- The emergence of resistance to β-lactam/β-lactamase inhibitor combinations represents an additional concern. Ceftazidime/avibactam-resistant carbapenem-resistant *K. pneumoniae* has been documented, indicating that even novel β-lactamase inhibitors face evolving resistance mechanisms [18].

#### 2.2.2. Fluoroquinolones

Fluoroquinolones, broad-spectrum antibiotics targeting DNA gyrase and topoisomerase IV, face substantial resistance challenges that limit their clinical utility. Fluoroquinolone resistance rates are alarmingly high across multiple pathogens and geographic regions. In Germany, fluoroquinolone resistance in Gram-negative pathogens ranges from moderate to high (5 to 25%), with *P. aeruginosa* demonstrating 24.9% resistance [9]. Over 50% of *E. coli* isolates in 5 out of 6 WHO regions show fluoroquinolone resistance [11]. A documented clinical case illustrates the consequences of fluoroquinolone resistance. A patient with underlying chronic obstructive pulmonary disease experienced ciprofloxacin treatment failure for a lower respiratory tract infection following prior ciprofloxacin therapy [20] The patient’s condition deteriorated, necessitating intensive care unit admission with bilateral pneumonia. Microbiological analysis revealed ciprofloxacin-resistant *S. pneumoniae* (serotype 11A) with an S79F mutation in parC and evidence of efflux pump activity [20]. The patient rapidly improved only after switching to azithromycin and ampicillin/sulbactam, demonstrating the clinical impact of fluoroquinolone resistance and the importance of avoiding fluoroquinolones after recent use of this class [20].

#### 2.2.3. Glycopeptides

Glycopeptide antibiotics, particularly vancomycin, have served as critical agents for treating Gram-positive infections, especially MRSA. However, the emergence of vancomycin resistance has severely compromised this therapeutic option.

- Vancomycin-resistant *Enterococci*: VREfm has increased significantly in Germany, with an odds ratio of 1.18 for temporal trends, reaching 34.9% prevalence in bloodstream infections [9]. The van gene clusters (vanA, vanB, vanC) mediate this resistance by remodeling cell wall precursors, reducing vancomycin binding affinity by approximately 1000-fold [15].
- Vancomycin-resistant and -intermediate *S. aureus*: The emergence of vancomycin-resistant *S. aureus* and vancomycin-intermediate *S. aureus* represents a critical threat, as vancomycin has been a cornerstone therapy for MRSA infections [17]. Linezolid-resistant *S. aureus* has also emerged, with 2.4% prevalence among MRSA isolates in Saudi Arabia [17].
- Colistin resistance: Colistin, often considered a ‘last-resort’ antibiotic for MDR Gram-negative infections, faces emerging resistance. In Greece, colistin resistance reaches 40% for *K. pneumoniae* and 47% for *Acinetobacter* spp., representing failure of even last-resort therapies [8].

#### 2.2.4. Other Classes

- Aminoglycosides: Resistance to aminoglycosides is mediated by aminoglycoside-modifying enzymes (aph(3′)-IIIa, aac(6′)-aph(2″), ant(6)-Ia) and 16S ribosomal RNA methyltransferases [15,17]. Target site alterations also affect newer aminoglycosides like plazomicin [19].
- Macrolides: Macrolide resistance involves erm genes encoding methylases that modify the 23S ribosomal RNA target, as well as efflux mechanisms [15].
- Mupirocin and antiseptics: Even topical agents face resistance, with plasmid-mediated mupA gene conferring mupirocin resistance through altered RNA synthetase, and qacA/B genes encoding efflux pumps that confer chlorhexidine resistance [16].

In summary, the limitations of existing antibacterial therapeutics represent a multifaceted crisis characterized by sophisticated resistance mechanisms, class-specific vulnerabilities, substantial clinical consequences, and devastating economic burdens. Bacteria have evolved diverse strategies to evade antibiotics, including enzymatic degradation (β-lactamases, carbapenemases, aminoglycoside-modifying enzymes), target site modifications (altered PBPs, DNA gyrase mutations, van gene clusters), efflux pumps, reduced permeability, and metabolic bypass mechanisms (Table 2). The convergence of these limitations—molecular resistance mechanisms, clinical treatment failures, mortality burdens, economic costs, and narrowing therapeutic options—underscores the urgent need for innovative approaches to antibacterial drug discovery. The integration of AI with bio-based product development, as explored in subsequent sections of this review, represents a promising frontier for identifying novel antibacterial agents capable of overcoming existing resistance mechanisms and addressing this escalating global health emergency.

## 3. Bottlenecks in Traditional Discovery of Bio-Based Natural Products

Despite the remarkable historical success of natural products in yielding transformative antibacterial agents such as penicillin, tetracycline, and vancomycin, the traditional natural product discovery (NPD) pipeline faces formidable challenges that have significantly constrained the identification of novel antibiotics in recent decades. The decline in new antibiotic approvals—with a greater than 50% reduction in new chemical entities approved by regulatory agencies—reflects the cumulative impact of multiple interconnected bottlenecks that plague conventional discovery approaches [21]. These challenges span the entire discovery continuum, from initial screening and compound identification through to lead optimization and development. Table 3 provides a comprehensive overview of the major bottlenecks in traditional NPD, including dereplication challenges, low hit rates in screening programs, difficulty accessing unculturable organisms, silent biosynthetic gene clusters (BGCs), and associated time and cost constraints. Each bottleneck is illustrated with empirical data, quantitative statistics, and real-world case studies that demonstrate the magnitude of these challenges.

The integration of AI with NPD, as explored in subsequent sections of this review, offers promising approaches to overcome many of these bottlenecks. AI-driven dereplication, predictive models for BGC activation, computational tools for prioritizing uncultured organisms, and machine learning (ML) approaches for hit prediction have the potential to dramatically improve the efficiency, speed, and cost effectiveness of NPD. By addressing the fundamental limitations of traditional approaches, AI integration may enable a renaissance in bio-based natural product-based antibacterial discovery, providing urgently needed solutions to the escalating crisis of AMR.

## 4. AI for Bio-Based Natural Product Discovery: An Overview

The integration of AI and ML into NPD has fundamentally transformed the field, enabling researchers to navigate the vast chemical space of bio-based natural compounds with unprecedented efficiency and precision. Traditional NPD approaches, characterized by labor-intensive isolation, purification, and bioactivity screening processes, are increasingly augmented or replaced by computational methodologies that leverage diverse AI architectures to predict bioactivity, elucidate structures, mine BGCs, and guide synthetic strategies. This paradigm shift is particularly critical in the context of antibacterial drug discovery, where the urgent need for novel antimicrobial agents demands accelerated identification and optimization of bioactive natural products.

Recent advances in AI methodologies have enabled the development of sophisticated tools and platforms that integrate multi-omics data, chemical informatics, and biological knowledge to streamline NPD pipelines. These computational approaches not only reduce the time and cost associated with traditional screening but also expand the accessible chemical space by predicting bioactivities of previously unexplored compounds and identifying cryptic biosynthetic pathways in microbial genomes. The following sections provide a comprehensive overview of major AI/ML methodologies, specific applications, performance metrics, and real-world case studies that demonstrate the transformative impact of AI in NPD.

### 4.1. Major AI/ML Methodologies in NPD

#### 4.1.1. ML Algorithms

Classical ML algorithms form the foundation of many NPD applications, offering interpretable models with robust performance across diverse prediction tasks. Random forest (RF) classifiers have demonstrated exceptional utility in bioactivity prediction, with models achieving area under the curve (AUC) values of 0.9 for predicting protein targets of bio-based natural compounds based on structural similarity to known drugs [33]. In a comprehensive study of natural compound bioactivity assessment, RF models trained on pairwise drug similarities yielded an average AUC of 0.9, Matthews correlation coefficient (MCC) of 0.35, and F1 score of 0.33, effectively capturing structure–activity relationships [33].

Support vector machines (SVMs) have been extensively employed for compound classification and bioactivity prediction, particularly in antiviral drug discovery. K-nearest neighbor (KNN) classifiers have proven particularly effective for integrating multi-omics data, as demonstrated by the Natural Products Mixed Omics (NPOmix) tool, which systematically connects mass spectrometry fragmentation spectra to BGCs independent of chemical class [34].

Gradient boosting machines represent another powerful ML approach, with the ChemoPy light gradient boosting machine model achieving AUC of 0.9371 and MCC of 0.7423 in predicting xanthine oxidase (XOD) inhibitors from a database of 315 compounds [35]. This model successfully identified three potential XOD inhibitors—daphnetin, 7-hydroxycoumarin, and piceatannol—from the FooDB database, with piceatannol exhibiting the strongest inhibitory activity upon experimental validation [35].

#### 4.1.2. Deep Learning Architectures

Deep learning (DL) methodologies have revolutionized NPD by enabling the processing of complex, high-dimensional data and learning hierarchical representations of molecular features. Convolutional neural networks (CNNs) have been successfully applied to molecular property prediction and compound–protein interaction modeling. The BioAct-Het framework employs a heterogeneous Siamese neural network architecture that achieved 88.5% accuracy in bioactivity prediction using novel bioactivity representations [36].

Recurrent neural networks and transformer architectures have demonstrated exceptional performance in sequence-based predictions for BGC analysis. Deep-BGCpred, a unified DL genome-mining framework, integrates multiple neural network architectures to achieve superior BGC detection performance, with precision improvements of 15 to 25% over existing tools [37]. Graph neural networks (GNNs) have emerged as particularly powerful for molecular representation learning, capturing topological and chemical features of bio-based natural products.

Self-supervised learning approaches have shown promise in learning meaningful representations without extensive labeled data. A self-supervised masked language model for BGC detection and product classification demonstrated improved downstream detection and classification performance by learning representations of BGCs and their constituent domains [38]. Transfer learning has proven effective in data-limited scenarios, with deep neural network transfer learning enabling chemical bioactivity predictions even when training data is scarce [39].

#### 4.1.3. Reinforcement Learning

Reinforcement learning (RL) represents an innovative approach to optimizing NPD workflows. A pioneering RL method for improving candidate BGC predictions in fungi achieved gene precision improvements exceeding 15% for tools like TOUCAN, fungiSMASH, and DeepBGC, and cluster precision improvements exceeding 25% for fungiSMASH and DeepBGC [37]. Evaluated on *A. niger* and *A. nidulans* genomes, this approach allowed tools to achieve near-perfect precision in cluster prediction, substantially reducing expert curation requirements [37].

### 4.2. Specific Applications of AI in NPD

#### 4.2.1. Compound Prediction and Virtual Screening

AI-driven virtual screening has dramatically accelerated the identification of bioactive natural products by enabling rapid computational evaluation of large compound libraries. The PECAN (Predicting Patterns of Cancer Cell Cytostatic Activity of Natural Products) platform utilizes DL to predict anticancer activity, achieving robust performance in identifying natural products with cytostatic effects against cancer cell lines [40]. For acetylcholinesterase inhibitor discovery, DL models applied to compounds isolated from *Pongamia pinnata* successfully identified potential inhibitors with high confidence [41].

Multi-target prediction frameworks have demonstrated particular utility in complex disease contexts. The OptNCMiner platform employs DL to discover bio-based natural compounds modulating disease-specific multi-targets, enabling simultaneous prediction of multiple therapeutic activities [42].

The integration of phenotypic profiling with chemical structure information has yielded powerful predictive models. A study combining Cell Painting phenotypic profiles with molecular structures achieved high accuracy in predicting compound activity, demonstrating the value of integrating morphological and chemical data [43]. For Nrf2 activator discovery, hybridization of in silico and in vitro bioassays enabled efficient identification of natural compounds with antioxidant and anti-inflammatory properties [44].

#### 4.2.2. Bioactivity Prediction

**Figure 1:**
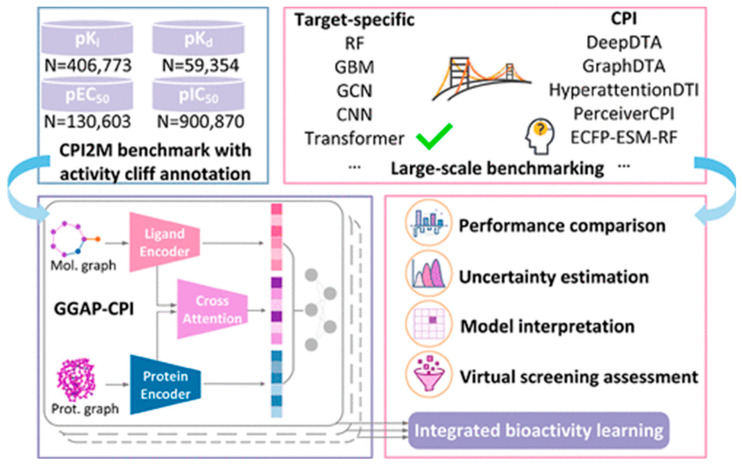
Overview of the GGAP-CPI model architecture and its application within the CPI2M benchmark, demonstrating a complex structure-free approach for compound–protein interaction prediction that integrates bioactivity learning to address data heterogeneity and activity cliffs. Reproduced from [45].

Bioactivity prediction represents one of the most mature applications of AI in NPD, with models achieving clinically relevant accuracy across diverse therapeutic targets. The BioAct-Het heterogeneous Siamese neural network achieved 88.5% accuracy in bioactivity prediction by learning from novel bioactivity representations that capture both structural and functional features [36]. For complex structure-free compound–protein interaction prediction, the Bioactivity Deep Learning framework demonstrated robust performance without requiring explicit 3D structural information (Figure 1) [45].

**Figure 2:**
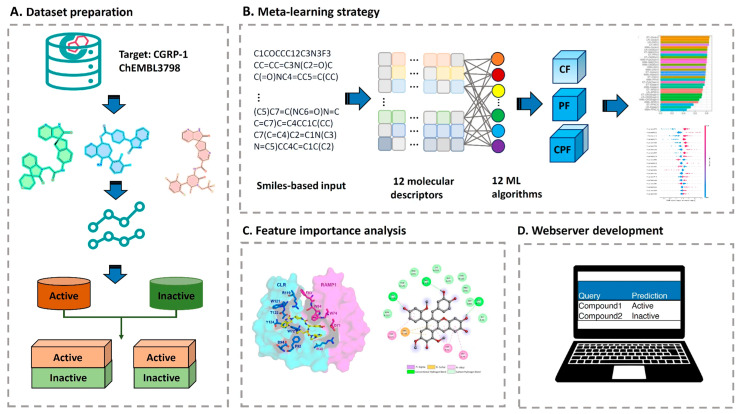
A schematic illustration of MetaCGRP, a method designed to identify CGRP inhibitors using only SMILES notation. (**A**) Dataset preparation and (**B**) meta-learning strategy. Reproduced from [47].

Multi-target deep neural network models have shown exceptional performance under increasingly challenging test conditions. A comprehensive evaluation of multi-target models for compound potency prediction revealed that DL architectures maintain high accuracy even when predicting activities against novel targets, with performance metrics remaining robust across diverse chemical scaffolds [46]. The MetaCGRP (calcitonin gene- related peptide) meta-model achieved high precision in large-scale identification of CGRP inhibitors using multi-view information, demonstrating the power of ensemble approaches (Figure 2) [47].

For specific therapeutic applications, DL has enabled discovery of novel antibacterial agents. A landmark study employing explainable DL discovered a new structural class of antibiotics with potent activity against drug-resistant bacteria, demonstrating that AI can identify compounds with mechanisms distinct from existing antibiotics [48]. For traditional Chinese medicine applications, DL approaches identified active ingredients of Yinchenhao Decoction targeting TLR4 for hepatic inflammatory diseases, achieving high prediction accuracy [49].

#### 4.2.3. BGC Mining and Detection

AI-driven BGC mining has transformed microbial genome analysis, enabling systematic discovery of cryptic biosynthetic pathways encoding novel bio-based natural products. A DL model specifically designed for BGC prediction in bacterial genomes demonstrated superior accuracy compared to traditional rule-based approaches, effectively identifying diverse BGC types, including polyketide synthases (PKS), non-ribosomal peptide synthetases (NRPS), and hybrid clusters [50].

The Seq2PKS platform represents a significant advancement in type I cis-AT polyketide discovery, combining computational mass spectrometry with genome mining to identify novel polyketide structures [51]. This integrated approach enables direct linking of mass spectrometry data to biosynthetic genes, accelerating the discovery of structurally diverse polyketides. For fungal BGC prediction, RL methods achieved precision improvements exceeding 25% for cluster boundary prediction, enabling more accurate identification of complete biosynthetic pathways [37].

Multi-label learning frameworks have enabled simultaneous prediction of chemical classes and biological activities directly from BGC sequences. A comprehensive multi-label model trained on the MIBiG database successfully predicted both the chemical class and bioactivity profile of natural products encoded by BGCs, achieving high accuracy across multiple prediction tasks [52]. Neural network approaches for predicting biological activity from BGCs have demonstrated that biosynthetic gene content alone can provide reliable predictions of compound bioactivity, even without prior knowledge of chemical structures [53].

#### 4.2.4. Structure Elucidation and Prediction

AI-driven structure elucidation has addressed one of the most challenging aspects of NPD—determining the complete chemical structure of novel compounds from spectroscopic data. This approach significantly reduces the time and material requirements traditionally associated with structure determination.

The NPOmix tool demonstrates the power of integrating MS with genomic data for structure prediction. By connecting MS/MS fragmentation spectra to BGCs using KNN classification, NPOmix successfully linked 18 previously known mass spectra (including 17 from the MIBiG database plus palmyramide A) to their corresponding experimentally validated biosynthetic genes [34]. This pattern-based genome mining approach facilitates specialized metabolite prioritization and works effectively on both pure cultures and complex microbiomes [34].

For polyketide structure prediction, the Seq2PKS framework combines DL with genome mining to predict polyketide structures directly from BGC sequences, achieving high accuracy in structure prediction for type I cis-AT polyketides [51]. This approach enables rapid structure prediction for compounds that have not yet been isolated, guiding prioritization of BGCs for experimental characterization.

#### 4.2.5. De Novo Molecule Generation and Optimization

Generative AI models have opened new frontiers in bio-based natural product-inspired drug design, enabling the creation of novel molecular structures with desired properties. Machine intelligence approaches for generating bioactive natural product-inspired molecules employ generative models to transform pharmacologically active natural products into new, easily synthesizable small molecules with optimized properties. These ‘virtual chemist’ approaches systematically transfer the wealth of pharmaceutically active natural products to synthetic small molecule drug discovery [54].

Molecular DL at the edge of chemical space has addressed the critical challenge of model generalization beyond training data. A joint modeling approach combining molecular property prediction with molecular reconstruction introduced ‘unfamiliarity,’ a reconstruction-based metric that enables estimation of model generalizability [55]. Across more than 30 bioactivity datasets, unfamiliarity effectively identified out-of-distribution molecules and served as a reliable predictor of classifier performance [55]. Experimental validation for 2 clinically relevant kinases discovered 7 compounds with low micromolar potency and limited similarity to training molecules, demonstrating that AI can extend discovery beyond the charted chemical space [55].

### 4.3. AI Tools and Platforms for NPD

The proliferation of specialized AI tools and platforms has democratized access to advanced computational methods for NPD.

- NPOmix provides a KNN-based classifier for systematically connecting MS fragmentation spectra to BGCs, facilitating siderophore mining and specialized metabolite prioritization [34]. The tool successfully validated 18 gene–metabolite pairs and works effectively on both bacterial cultures and environmental microbiomes [34].
- BioAct-Het offers a heterogeneous Siamese neural network architecture for bioactivity prediction, achieving 88.5% accuracy through novel bioactivity representations [36]. PECAN employs DL to predict anticancer activity of bio-based natural products, enabling rapid screening of compound libraries for cytostatic effects [40].
- OptNCMiner provides a DL framework for discovering bio-based natural compounds that modulate disease-specific multi-targets, enabling simultaneous prediction of multiple therapeutic activities [42]. MetaCGRP represents a high-precision meta-model for large-scale identification of CGRP inhibitors using multi-view information, demonstrating the power of ensemble approaches in natural product screening [47].
- For BGC mining, Seq2PKS combines computational MS with genome mining for type I cis-AT polyketide discovery, enabling direct linking of MS data to biosynthetic genes [51]. An RL tool for improving fungal BGC predictions is available at https://github.com/bioinfoUQAM/RL-bgc-components, providing open-source access to advanced BGC refinement methods [37].

### 4.4. Integration of AI with Traditional Approaches

The most successful NPD programs integrate AI methodologies with traditional experimental approaches, creating synergistic workflows that leverage the strengths of both computational and laboratory-based methods. The hybridization of in silico and in vitro bioassays for studying Nrf2 activation by natural compounds exemplifies this integration, combining computational predictions with experimental validation to efficiently identify bioactive compounds [44].

For XOD inhibitor discovery, a comprehensive pipeline integrated ML Quantitative Structure–Activity Relationships (QSAR) modeling with molecular docking, molecular dynamics simulations, ADME predictions, and in vitro enzyme inhibition assays [35]. This integrated approach identified piceatannol as a potent XOD inhibitor, with computational predictions validated through experimental enzyme inhibition assays demonstrating the strongest inhibitory activity among tested compounds [35].

The NPOmix workflow demonstrates effective integration of metabolomics and genomics, linking MS data to BGCs to accelerate drug discovery programs [34]. This integrative omics mining approach minimizes the need for extensive culturing and facilitates metabolite prioritization based on both chemical and genetic information [34]. Similarly, the Seq2PKS platform integrates computational MS with genome mining, enabling the discovery of type I cis-AT polyketides through combined analysis of spectroscopic and genomic data [51].

For bioactivity prediction, the integration of phenotypic profiling (Cell Painting) with chemical structure information has yielded models with superior predictive performance compared to structure-only approaches [43]. This multi-modal integration captures both morphological effects and chemical features, providing a more comprehensive assessment of compound bioactivity. The combination of ML models with target similarity analysis between drugs has enabled effective bioactivity assessment of bio-based natural compounds, with experimental validation confirming Cox-1 inhibition by 5-methoxysalicylic acid as predicted computationally [33].

### 4.5. Performance Metrics and Comparative Analysis

Rigorous evaluation of AI models through standardized performance metrics is essential for assessing their utility in NPD. Across bioactivity prediction applications, models consistently achieve [55] values ranging from 0.90 to 0.94, indicating excellent discriminative ability. The RF model for natural compound bioactivity assessment achieved an AUC of 0.9, an MCC of 0.35, and an F1 score of 0.33 [33], while the gradient boosting machine for XOD inhibitor prediction achieved an AUC of 0.9371 and an MCC of 0.7423 [35]. The BioAct-Het heterogeneous Siamese neural network achieved 88.5% accuracy in bioactivity prediction [36].

For BGC detection and refinement, RL approaches demonstrated substantial improvements over existing tools, with gene precision improvements exceeding 15% and cluster precision improvements exceeding 25% [37]. These improvements enabled near-perfect precision in cluster prediction for tools like fungiSMASH and DeepBGC when applied to *Aspergillus* genomes [37].

Dataset sizes vary considerably across applications, reflecting the availability of training data for different prediction tasks. The XOD inhibitor prediction model was trained on 315 compounds [35]. The natural compound bioactivity assessment study trained models on pairwise similarities between 1410 drugs and applied them to predict targets of approximately 11,000 natural compounds [33]. The unfamiliarity-based molecular DL approach was validated across more than 30 bioactivity datasets, discovering seven compounds with low micromolar potency through experimental validation [55].

Comparison of AI with traditional approaches reveals significant advantages in throughput, cost efficiency, and ability to explore chemical space. The NPOmix tool successfully linked 18 mass spectra to biosynthetic genes, a task that would require extensive experimental effort using traditional approaches involving nuclear magnetic resonance (NMR) or genetic engineering [34]. The integration of AI with experimental validation has proven particularly effective, as demonstrated by the XOD inhibitor study where computational predictions guided experimental testing, with piceatannol exhibiting the strongest inhibitory activity among predicted candidates [35].

## 5. AI-Driven Genome Mining for Novel Biosynthetic Pathways

The exponential growth of microbial genomic data has revealed an unprecedented reservoir of biosynthetic potential, with bacterial and fungal genomes harboring thousands of BGCs encoding diverse bio-based natural products. However, the vast majority of these BGCs remain cryptic or silent under standard laboratory conditions, representing an untapped source of novel antibacterial compounds. AI and ML have emerged as transformative technologies for genome mining, enabling systematic detection, classification, and prioritization of BGCs across large genomic datasets. These computational approaches have fundamentally changed the paradigm of NPD, shifting from serendipitous isolation to rational, genome-guided identification of biosynthetic pathways.

AI-driven genome mining encompasses a diverse array of computational methodologies, from traditional ML classifiers to sophisticated DL architectures, each designed to address specific challenges in BGC detection and characterization. These tools must navigate the complexity of microbial genomes, distinguishing biosynthetic genes from primary metabolism while accurately predicting BGC boundaries, classifying product types, and assessing novelty. The integration of AI with experimental validation workflows has accelerated the discovery of novel natural products, with several recent studies demonstrating successful identification and characterization of previously unknown antibacterial compounds through genome-mining approaches.

### 5.1. AI/ML Methodologies for BGC Prediction and Detection

#### 5.1.1. DL Architectures

**Figure 3:**
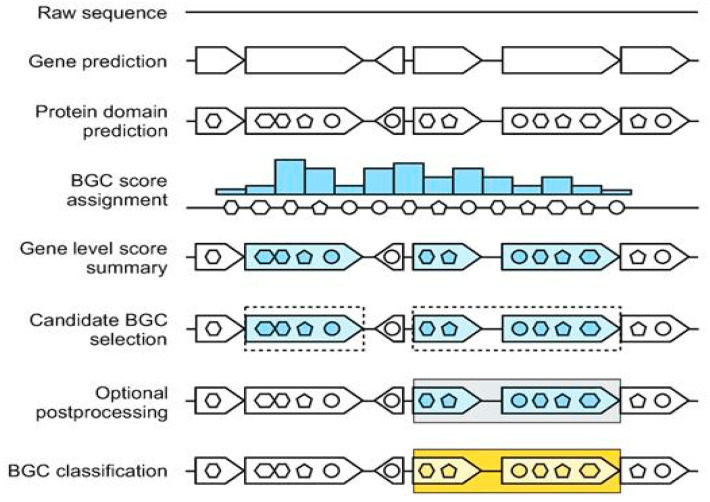
DL-based detection of BGCs in bacterial genomes. Reproduced from [56].

DL has revolutionized BGC detection by enabling the learning of complex patterns from genomic sequences without extensive feature engineering. DeepBGC, a pioneering DL tool, employs recurrent neural networks with Long Short-Term Memory (LSTM) cells for BGC identification (Figure 3), supplemented with RF classifiers for predicting BGC product classes and chemical activity [56]. This architecture offers reduced false-positive rates and improved ability to identify novel BGC classes compared to existing ML tools, uncovering previously undetectable putative BGCs in bacterial genomes [56].

Building upon DeepBGC, e-DeepBGC extends the original architecture by incorporating validated BGCs, protein family domains (Pfams), Pfam functions, and clan information as input features [50]. Applied to 5666 RefSeq bacterial genomes, e-DeepBGC detected 170,685 BGCs with an average of 30.1 BGCs per genome, demonstrating increased sensitivity in identifying BGCs and reduced false-positive rates compared to its predecessor [50].

#### 5.1.2. Self-Supervised Learning Approaches

Self-supervised learning has emerged as a powerful paradigm for BGC detection, enabling models to learn meaningful representations from unlabeled genomic data. BiGCARP, a self-supervised masked language model based on CNNs using ByteNet and CARP architectures, incorporates pretrained protein embeddings from Evolutionary Scale Modeling (ESM)-1b for feature extraction [38]. The BiGCARP-ESM-1b-finetuned model achieved 75% accuracy on the self-supervised dataset and outperformed DeepBGC on average AUC across product classes, with AUC values of 0.898 for both polyketide and non-ribosomal peptide classes [38]. The model was pretrained on 127,294 BGCs and applied to 773 unannotated bacterial genomes, identifying 4287 clusters with 3174 matching Pfam domains [38]. Notably, BiGCARP identified 199 possible BGC start locations with better scores than antiSMASH (antibiotics & Secondary Metabolite Analysis Shell) 6.1.1, demonstrating the potential of self-supervised learning to improve upon established tools [38].

The self-supervised approach represents BGCs as chains of functional protein domains, allowing the model to learn contextual relationships between biosynthetic enzymes without requiring extensive manual annotation. This methodology is particularly valuable for detecting novel BGC classes that may not be well-represented in training databases, as the model learns general principles of biosynthetic organization rather than memorizing specific patterns.

#### 5.1.3. Natural Language Processing and Transformer Models

The conceptualization of BGCs as biological ‘sentences’ composed of protein domain ‘words’ has enabled the application of natural language processing (NLP) techniques to genome mining. BGCCGB integrates BERT (Bidirectional Encoder Representations from Transformers) and its self-attention mechanism with Graph Convolutional Networks (GCNs) for BGC identification and product type classification [57]. The model represents BGCs as sequences of protein family (Pfam) identifiers and employs a synonym replacement method for data augmentation, learning both local and global features [57]. BGCCGB achieved a 6.9% average precision improvement in BGC identification over previous methods, effectively localizing BGCs in bacterial genomes and predicting their product types [57].

BGC-Prophet, a language model based on the transformer encoder, utilizes neural networks to capture distant location-dependent relationships among biosynthetic genes [58]. This ultrahigh-throughput method is several orders of magnitude faster than existing tools like DeepBGC, demonstrating superior performance in both accuracy and speed [58]. BGC-Prophet analyzed 85,203 genomes and 9428 metagenomes, revealing a profound enrichment of BGCs, particularly polyketides, after the Great Oxidation and Cambrian Explosion events [58]. This evolutionary perspective demonstrates how AI-driven genome mining can provide insights into the historical development of biosynthetic capabilities across geological timescales.

An NLP-based approach employing CountVec, TFIDF, and Word2Vec embeddings combined with physicochemical properties achieved 0.96 accuracy and 0.9912 AUC in BGC classification using a logistic regression model with TFIDF and SMOTE [59]. This demonstrates that even relatively simple NLP techniques, when properly integrated with domain-specific features, can achieve excellent classification performance.

#### 5.1.4. RF and Ensemble Methods

Classical ML approaches, particularly RF classifiers, continue to demonstrate competitive performance in BGC prediction tasks. RFBGCPred employs RF as the top-performing classifier, using Word2Vec for feature extraction, supervised UMAP for dimensionality reduction, and SMOTE to address class [60]. Focused on five clinically and agriculturally important classes—PKS, NRPS, Ribosomally Synthesized and Post-translationally Modified Peptides (RiPPs), terpenes, and PKS-NRPS hybrids—RFBGCPred achieved an accuracy of 98.0% (MCC: 0.9752, AUC: 0.9928) on a balanced test set [60]. On an unbalanced validation set, the model maintained strong generalization with 94.8% accuracy (MCC: 0.89, AUC: 0.96) [60]. Compared with antiSMASH and DeepBGC, RFBGCPred showed improved recall for hybrid PKS-NRPS clusters while sustaining competitive precision, thereby reducing misclassification of atypical arrangements [60].

**Figure 4:**
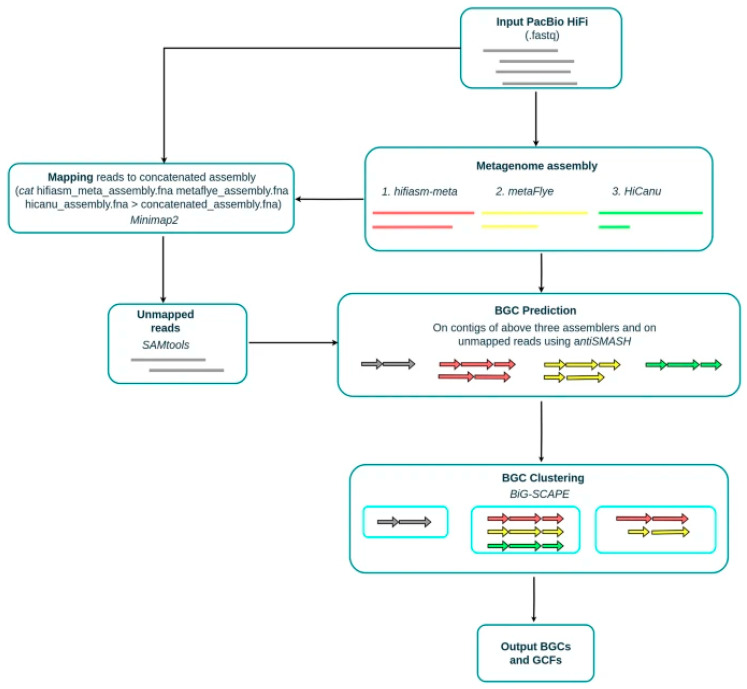
The HiFiBGC workflow for detecting BGCs from PacBio HiFi metagenomic data. The process begins with assembly of reads by three tools (hifiasm-meta, metaFlye, HiCanu). Reads are then mapped to the combined assembly to capture unmapped reads. Finally, BGCs are predicted on all assemblies and unmapped reads with antiSMASH, and subsequently clustered with BiG-SCAPE to produce the final BGC predictions. Reproduced from [61].

HiFiBGC demonstrates the power of ensemble approaches for BGC detection in metagenomic data, leveraging an ensemble of assemblies from three HiFi-tailored metagenome assemblers (Figure 4) [61]. Based on analyses of four HiFi metagenomic datasets from diverse environments, HiFiBGC identified on average 78% more BGCs than the top-performing single-assembler-based method, with the ensemble assembly approach improving recovery by 25% [61]. This substantial improvement highlights the value of ensemble strategies in capturing the full diversity of BGCs present in complex metagenomic samples.

#### 5.1.5. RL for BGC Refinement

RL represents an innovative approach to improving BGC predictions by iteratively refining cluster boundaries and gene assignments. An RL method for improving candidate BGC predictions in fungi achieved gene precision improvements exceeding 15% for tools like TOUCAN, fungiSMASH, and DeepBGC, and cluster precision improvements exceeding 25% for fungiSMASH and DeepBGC [37]. Evaluated on *A. niger* and *A. nidulans* genomes, this approach allowed tools to achieve almost perfect precision in cluster prediction, substantially reducing the need for expert manual curation [37]. The RL framework relies on protein domains and functional annotations from expert-curated BGCs, learning optimal strategies for including or excluding genes from predicted clusters based on their functional relevance to secondary metabolism.

### 5.2. Major AI Tools and Platforms for Genome Mining

#### 5.2.1. antiSMASH: The Gold Standard

antiSMASH remains the most widely used tool for BGC prediction, serving as the benchmark against which newer AI-driven tools are evaluated. While antiSMASH primarily employs rule-based approaches using Hidden Markov Models for protein domain detection, it has incorporated ML components in recent versions [38]. The antiSMASH training set is approximately 100-times larger than the MIBiG database, providing extensive coverage of known BGC types [38]. Multiple studies have compared novel AI tools against antiSMASH, with tools like BiGCARP identifying 199 possible BGC start locations with better scores than antiSMASH 6.1.1 [38].

#### 5.2.2. DeepBGC and Extensions

DeepBGC pioneered the application of DL to BGC detection, employing bidirectional LSTM recurrent neural networks for sequence analysis [56]. The tool offers reduced false-positive rates and improved ability to extrapolate and identify novel BGC classes compared to existing ML tools [56]. DeepBGC also incorporates RF classifiers for predicting BGC product classes and potential chemical activity, providing multi-level predictions from detection to functional annotation [56]. e-DeepBGC extends the original DeepBGC architecture by incorporating additional features including validated BGCs, protein family domains, Pfam functions, and clan information [50]. This enhanced feature set enables more accurate BGC detection and classification, with the tool successfully applied to large-scale genome mining of 5666 RefSeq bacterial genomes [50].

#### 5.2.3. Specialized Tools for Specific BGC Types

Seq2PKS represents a specialized tool for discovering type I cis-AT polyketides through integration of computational MS and genome mining [51]. The ML algorithm predicts chemical structures derived from type I PKS, generating numerous putative structures for each gene cluster to enhance accuracy [51]. The correct structure is identified using variable mass spectral database search, with benchmarks showing that Seq2PKS outperforms existing methods [51]. Application of Seq2PKS to Actinobacteria datasets led to the discovery of BGCs for monazomycin, oasomycin A, and 2-aminobenzamide-actiphenol [51].

**Figure 5:**
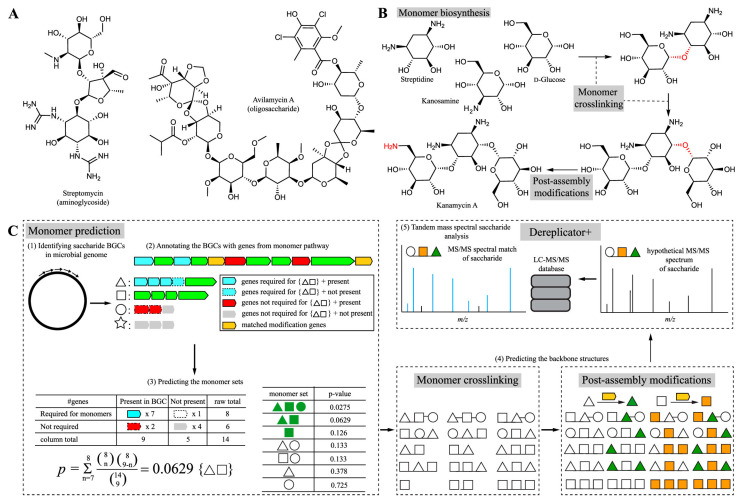
The Seq2Saccharide pipeline is designed to predict the structures of candidate saccharides based on their BGCs. (**A**) Representative structures of aminoglycosides and oligosaccharides that are targeted by Seq2Saccharide. (**B**) The biosynthetic logic underlying saccharide natural products targeted by Seq2Saccharide, illustrated through the biosynthesis of kanamycin A. (**C**) Overview of the Seq2Saccharide pipeline. Reproduced from [62].

Seq2Saccharide addresses the challenge of oligosaccharide and aminoglycoside discovery by integrating computational MS with genome mining (Figure 5) [62]. The tool predicts hundreds or thousands of putative structures for each gene cluster, with the correct structure identified through mass spectral search. Benchmarks against saccharides in the MiBIG database demonstrate that Seq2Saccharide outperforms existing methods in predicting saccharide structures, with MS analysis indicating that the variable search module can correct mispredictions from genome mining. The tool successfully identified the BGC for the polysaccharide oligosaccharide trestatin B through integrated analysis of genomic and MS data [62].

SeMPI 2.0 provides a refined rule-based pipeline for precise PKS and NRPS predictions combined with metabolite screening in bio-based natural product databases [63]. The tool focuses on high-quality scaffold predictions through thoroughly refined and benchmarked cluster detection algorithms, including building block generation and domain substrate prediction. In a benchmark based on 559 gene clusters, SeMPI 2.0 achieved comparable or better results than antiSMASH v5. The screening algorithm was designed to detect homologous structures even for partial, incomplete clusters, enabling the linking of gene clusters to known natural products and providing a metric to estimate cluster novelty [63].

#### 5.2.4. Tools for Targeted BGC Family Expansion

**Figure 6:**
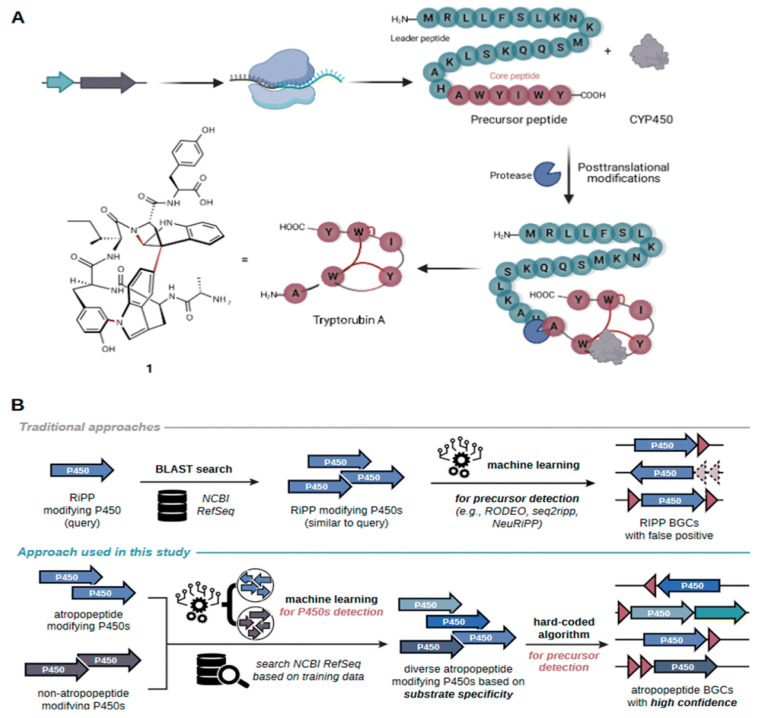
Schematic overview of the proposed model for tryptorubin A (1) biosynthesis and comparison of the genome mining concept used in AtropoFinder to state-of-the-art genome mining tools for the identification of RiPP BGCs. (**A**) Within the trp BGC, the genes are transcribed, and the resulting mRNA is ribosomally translated. The translated precursor peptide, which contains both a core peptide and a leader peptide, then undergoes posttranslational modification by a BGC-encoded cytochrome P450. This enzyme installs the characteristic crosslinks of atropopeptides. Finally, a ubiquitous protease removes the leader peptide, yielding the mature hexapeptide tryptorubin A (1). (**B**) A comparison between existing ML approaches for RiPP discovery and the strategy developed in this study. Current state-of-the-art tools typically rely on BLAST or Hidden Markov Model-based searches to identify putative RiPP-modifying tailoring enzymes. Following this, ML algorithms are employed to locate potential precursor genes in the genomic proximity of those tailoring enzymes. By contrast, this approach first utilized ML to precisely identify tailoring enzymes responsible for atropopeptide modifications. In a subsequent step, a hard-coded algorithm is applied to detect the corresponding precursor genes with a high degree of confidence. Reproduced from [64].

AtropoFinder, an ML-based algorithm, was developed specifically to explore atropopeptide BGCs, identifying over 650 atropopeptide BGCs (Figure 6) [64]. This study used bioinformatics and modeling analyses to pinpoint crucial motifs and residues in leader and core peptide sequences. Four atropopeptide BGCs were heterologously expressed, leading to the identification of novel atropopeptides with varying lengths and modifications, including one characterized BGC that produced two peptides with the same sequence but distinct modification patterns [64]. This work substantially expanded the atropopeptide chemical space, demonstrating the power of targeted ML approaches for specific RiPP families.

#### 5.2.5. Workflow and Integration Platforms

**Figure 7:**
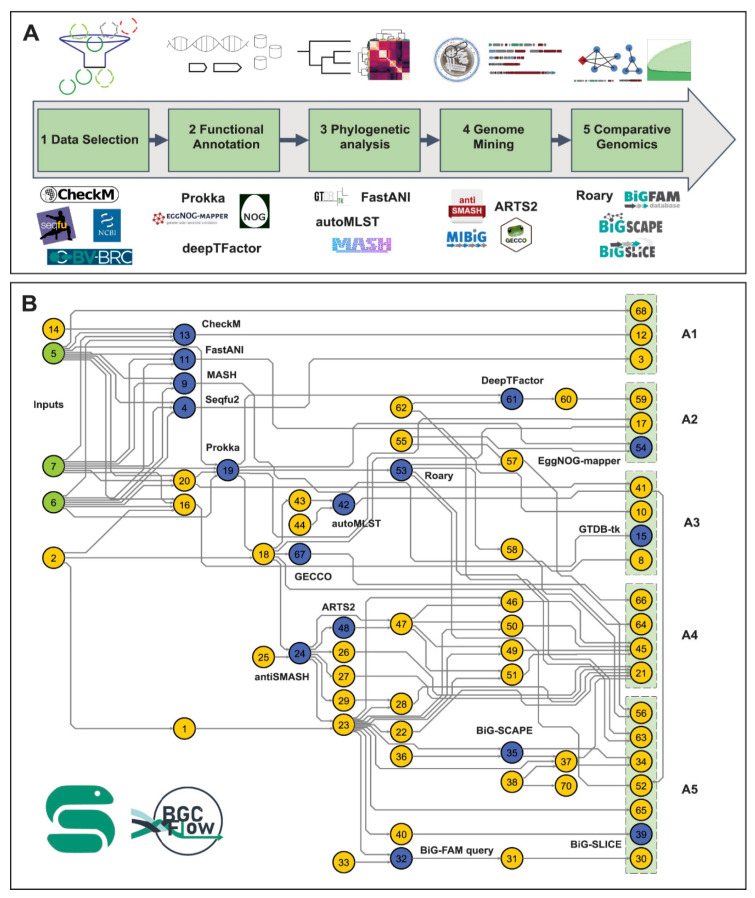
BGCFlow supported rules to carry out common stages of large-scale genome mining studies. (**A**) An overview of the five primary analysis stages supported by BGCFlow. (**B**) A detailed Snakemake rulegraph illustrating the BGCFlow architecture. Input nodes are depicted in green, nodes representing the main bioinformatic tools are shown in blue, and custom rules designed for interoperability are highlighted in yellow. The nodes within the green boxes on the right serve as endpoints for individual pipelines, which can be enabled or disabled through configuration files. These endpoint custom rules correspond to the five main analysis categories: (A1) Data Selection, (A2) Functional Annotation, (A3) Phylogenomic placement, (A4) Genome Mining, and (A5) Comparative genomics. Reproduced from [65].

BGCFlow provides a systematic workflow integrating analytics for large-scale genome mining of bacterial pangenomes [65]. The platform incorporates several genome analytics and mining tools grouped into five common stages: data selection, functional annotation, phylogenetic analysis, genome mining, and comparative analysis (Figure 7). BGCFlow provides easy configuration of different projects, parallel distribution, scheduled job monitoring, an interactive database to visualize tables, exploratory Jupyter Notebooks, and customized reports. Application of BGCFlow to 42 genomes of the *Saccharopolyspora* genus predicted more accurate dereplication of BGCs and guided targeted comparative analysis of selected RiPPs. The scalable, interoperable, adaptable, re-entrant, and reproducible nature of BGCFlow provides an effective way to extract biosynthetic knowledge from ever-growing genomic datasets [65].

SYNTERUPTOR employs a complementary approach by mining genomic islands for non-classical specialized metabolite gene clusters [66]. The program identifies genomic islands, known to be enriched in SMBGCs, in the genomes of closely related species. Using this tool, researchers identified a SMBGC in the genome of *Streptomyces ambofaciens* ATCC23877 that was undetected by earlier versions of antiSMASH, experimentally demonstrating that it directs the biosynthesis of two metabolites, one of which was identified as sphydrofuran. SYNTERUPTOR is also valuable for delineating individual SMBGCs within antiSMASH regions that may encompass multiple clusters and for refining cluster boundaries [66].

### 5.3. BGC Types Targeted by AI Tools

#### 5.3.1. Polyketide Synthases

PKSs represent one of the most extensively studied BGC types, encoding enzymes that produce structurally diverse compounds with significant pharmaceutical applications. AI tools have demonstrated particular success in PKS detection and characterization. RFBGCPred specifically targets PKS clusters as one of its five focus classes, achieving 98.0% accuracy with improved recall for hybrid PKS-NRPS clusters [60]. BiGCARP achieved area under receiver operating characteristic (AUROC) of 0.898 for polyketide classification [38], while BGC-Prophet revealed a surge in polyketide BGC diversity and abundance after important geological events [58].

Specialized tools for PKS analysis have enabled discovery of novel polyketide natural products. Seq2PKS successfully identified BGCs for monazomycin, oasomycin A, and 2-aminobenzamide-actiphenol through integrated computational MS and genome mining [51]. SeMPI 2.0 provides precise PKS predictions with performance comparable to or better than antiSMASH v5 in benchmarks of 559 gene clusters [63]. HiFiBGC’s ensemble approach proved particularly valuable for recovering complete PKS clusters from metagenomic data, as these clusters are often long and repetitive, challenging for short-read assembly [61].

#### 5.3.2. Non-Ribosomal Peptide Synthetases

NRPSs produce a diverse array of peptide natural products, including many clinically important antibiotics. AI tools have achieved high accuracy in NRPS detection and classification. BiGCARP achieved an AUC of 0.898 for non-ribosomal peptide classification [38], while RFBGCPred targets NRPS as one of its core classes with 98.0% overall accuracy [60]. The NLP-based approach using TFIDF embeddings achieved 0.96 accuracy across 5 natural product classes including NRPS [59].

SeMPI 2.0 provides specialized NRPS predictions with refined building block generation and domain substrate prediction, achieving results comparable to or better than antiSMASH v5 [63]. Like PKS clusters, NRPS BGCs are often long and repetitive, making them challenging targets for assembly from short-read data. HiFiBGC’s ensemble approach addresses this limitation, identifying 78% more BGCs on average than single-assembler methods, with particular benefits for recovering complete NRPS clusters [61].

#### 5.3.3. Hybrid PKS-NRPS Clusters

Hybrid PKS-NRPS clusters represent a particularly challenging target for BGC detection due to their complex, atypical architectures combining features of both polyketide and non-ribosomal peptide biosynthesis. RFBGCPred specifically addresses this challenge, demonstrating improved recall for hybrid PKS-NRPS clusters compared to antiSMASH and DeepBGC while maintaining competitive precision [60]. The tool’s focus on this hybrid class, combined with Word2Vec feature extraction and SMOTE-based handling of class imbalance, enables more accurate identification of these atypical arrangements [60]. The 98.0% accuracy on balanced test sets and 94.8% accuracy on unbalanced validation sets demonstrates robust performance across diverse cluster architectures [60].

#### 5.3.4. RiPPs

RiPPs represent a rapidly expanding class of natural products with diverse structures and bioactivities. AI tools have demonstrated particular success in RiPP discovery and characterization. RFBGCPred targets RiPPs as one of its five cores [60], while BiGCARP includes RiPPs among its target product classes [38]. BGCFlow-guided analysis enabled targeted comparative analysis of selected RiPPs across 42 Saccharopolyspora genomes [65]. Specialized tools for RiPP discovery have enabled identification of novel peptide families. AtropoFinder identified over 650 atropopeptide BGCs, with heterologous expression of 4 BGCs leading to discovery of novel atropopeptides with varying lengths and modifications [64].

#### 5.3.5. Terpenes

Terpene BGCs encode enzymes producing a vast array of structurally diverse compounds with applications ranging from antibiotics to anticancer agents. RFBGCPred specifically targets terpenes as one of its five focus classes, achieving 98.0% overall accuracy [60]. BiGCARP includes terpenes among its target product classes, achieving strong classification performance [38]. The NLP-based approach using TFIDF embeddings achieved 0.96 accuracy across five natural product classes, including terpenes [59].

#### 5.3.6. Saccharides and Aminoglycosides

Oligosaccharides and aminoglycosides represent important sources of antibacterial compounds, but their complex biosynthesis pathways have historically made structure prediction challenging. These compound classes present unique challenges for AI-driven discovery due to three factors. First, the non-linear relationship between modifying enzymes and product structures means that the order and stereochemistry of glycosyltransferase reactions create complex branching patterns that linear sequence-based models struggle to predict. Second, extensive post-biosynthetic modifications including variable methylation, acetylation, and phosphorylation generate structural microheterogeneity that complicates structure prediction. Third, mass spectrometric differentiation of stereoisomers remains difficult because epimers and anomers produce nearly identical fragmentation patterns despite having distinct biological activities.

Seq2Saccharide addresses this gap by integrating computational MS with genome mining specifically for saccharide natural product discovery [62]. The tool predicts hundreds or thousands of putative structures for each gene cluster, with benchmarks against the MiBIG database demonstrating superior performance compared to existing methods [62]. Seq2Saccharide successfully identified the BGC for trestatin B, validating its integrated approach to saccharide discovery [62]. BiGCARP includes saccharides among its target product classes [38], expanding the range of AI tools capable of detecting these important BGC types.

### 5.4. Integration of Genome Mining with Experimental Validation

#### 5.4.1. Heterologous Expression and Compound Isolation

The ultimate validation of AI-driven genome mining predictions requires experimental characterization of predicted BGCs and isolation of their products. Several recent studies have demonstrated successful integration of computational predictions with experimental validation. AtropoFinder’s identification of over 650 atropopeptide BGCs was validated through heterologous expression of four selected clusters, leading to the discovery of novel atropopeptides with varying lengths and modifications [64]. One characterized BGC encoding a single P450 was found to be involved in the biosynthesis of two peptides with the same sequence but distinct modification patterns, revealing unexpected biosynthetic complexity [64]. SYNTERUPTOR’s identification of a previously undetected SMBGC in *S. ambofaciens* ATCC23877 was experimentally validated, demonstrating that the cluster directs biosynthesis of two metabolites, one identified as sphydrofuran [66].

#### 5.4.2. MS Integration

Integration of genome mining with MS provides a powerful approach for linking predicted BGCs to their molecular products. Seq2PKS combines ML-based structure prediction with variable mass spectral database search, enabling discovery of BGCs for monazomycin, oasomycin A, and 2-aminobenzamide-actiphenol from *Actinobacteria* datasets [51]. The tool’s ability to predict numerous putative structures for each gene cluster, followed by mass spectral matching, substantially improves accuracy compared to methods that predict single structures [51].

Seq2Saccharide demonstrates similar integration for oligosaccharide and aminoglycoside discovery, with MS analysis indicating that the variable search module can correct mispredictions from genome mining [62]. The successful identification of the trestatin B BGC through integrated genomic and MS analysis validates this approach [62]. This bidirectional integration—using genomics to guide MS analysis and MS to validate genomic predictions—represents a powerful paradigm for NPD.

### 5.5. Novel Biosynthetic Pathways Discovered Through AI Approaches

#### 5.5.1. Large-Scale BGC Discovery

AI-driven genome mining has enabled unprecedented large-scale discovery of biosynthetic pathways. e-DeepBGC’s application to 5666 RefSeq bacterial genomes detected 170,685 BGCs, averaging 30.1 BGCs per genome [50]. This massive dataset reveals the extraordinary biosynthetic potential encoded in bacterial genomes, with the vast majority of these BGCs representing unexplored biosynthetic pathways.

BiGCARP’s analysis of 773 unannotated bacterial genomes identified 4287 clusters, with 3174 matching Pfam domains, and identified 199 possible BGC start locations with better scores than antiSMASH 6.1.1 [38]. BGC-Prophet’s analysis of 85,203 genomes and 9428 metagenomes revealed evolutionary patterns in BGC distribution, including enrichment of polyketide BGCs after major geological events [58]. These large-scale analyses demonstrate that AI tools are uncovering biosynthetic pathways at a scale impossible with traditional approaches.

#### 5.5.2. Novel Natural Product Families

AI-driven genome mining has enabled the discovery of entirely new families of natural products. AtropoFinder’s identification of over 650 atropopeptide BGCs substantially expanded this RiPP family, with heterologous expression revealing atropopeptides more extensively modified than previously identified members [64]. The discovery that a single P450-encoding BGC produces two peptides with identical sequences but distinct modification patterns reveals previously unknown biosynthetic mechanisms [64].

Seq2PKS’s discovery of BGCs for monazomycin, oasomycin A, and 2-aminobenzamide-actiphenol from *Actinobacteria* datasets demonstrates the power of integrating ML with MS for polyketide discovery [51].

#### 5.5.3. Cryptic and Silent BGCs

A major advantage of AI-driven genome mining is the ability to identify cryptic or silent BGCs that are not expressed under standard laboratory conditions. SYNTERUPTOR’s identification of a previously undetected SMBGC in *S. ambofaciens* ATCC23877, missed by earlier versions of antiSMASH, demonstrates the ability of AI tools to uncover cryptic biosynthetic pathways [66]. Experimental validation confirmed that this cluster directs biosynthesis of sphydrofuran and another metabolite [66]. DeepBGC’s ability to uncover previously undetectable putative BGCs that may code for bio-based natural products with novel biologic activities demonstrates that AI tools are expanding access to the ‘dark matter’ of microbial biosynthetic potential [56].

### 5.6. Performance Metrics and Comparative Analysis

#### 5.6.1. Detection Accuracy and Precision

AI tools for BGC detection have achieved impressive performance metrics across diverse evaluation datasets. RFBGCPred achieved 98.0% accuracy (MCC: 0.9752, AUC: 0.9928) on a balanced test set, maintaining 94.8% accuracy (MCC: 0.89, AUC: 0.96) on an unbalanced validation set [60]. The NLP-based approach using logistic regression with TFIDF embeddings achieved 0.96 accuracy and 0.9912 AUC for BGC classification [59]. BiGCARP-ESM-1b-finetuned achieved 75% accuracy on the self-supervised dataset, with AUC values of 0.898 for both polyketide and non-ribosomal peptide classification [38]. BGCCGB achieved a 6.9% average precision improvement in BGC identification over previous methods [57].

#### 5.6.2. Comparison with Established Tools

Multiple studies have benchmarked novel AI tools against established platforms like antiSMASH and DeepBGC. RFBGCPred showed improved recall for hybrid PKS-NRPS clusters compared to antiSMASH and DeepBGC while sustaining competitive precision [60]. BiGCARP identified 199 possible BGC start locations with better scores than antiSMASH 6.1.1 [38]. SeMPI 2.0 achieved comparable or better results than antiSMASH v5 in a benchmark of 559 gene clusters [63]. RL approaches improved gene precision by above 15% for TOUCAN, fungiSMASH, and DeepBGC, with cluster precision improvements exceeding 25% for fungiSMASH and DeepBGC [37]. These improvements enabled near-perfect precision in cluster prediction for fungal genomes [37].

#### 5.6.3. Scalability and Computational Efficiency

Computational efficiency is critical for large-scale genome mining applications. BGC-Prophet demonstrated performance several orders of magnitude faster than existing tools like DeepBGC, enabling analysis of 85,203 genomes and 9428 metagenomes [58]. This ultrahigh-throughput capability enables pan-phylogenetic and whole-metagenome screening at unprecedented scales [58].

HiFiBGC’s ensemble approach, while computationally more intensive than single-assembler methods, identified 78% more BGCs on average, with the ensemble assembly approach improving recovery by 25% [61]. BGCFlow’s scalable, interoperable, adaptable, re-entrant, and reproducible architecture provides effective extraction of biosynthetic knowledge from ever-growing genomic datasets [65]. The platform’s integration of parallel distribution and scheduled job monitoring enables efficient processing of large pangenomic datasets [65].

#### 5.6.4. Dataset Sizes and Training Data

The scale of training and evaluation datasets varies considerably across AI tools, reflecting different approaches to model development. BiGCARP was pretrained on 127,294 BGCs, with the antiSMASH training set approximately 100-times larger than the MIBiG database [38]. e-DeepBGC was applied to 5666 RefSeq bacterial genomes, detecting 170,685 BGCs [50]. BiGCARP analyzed 773 unannotated bacterial genomes, identifying 4287 clusters [38]. SeMPI 2.0 was benchmarked on 559 gene clusters [63], while the RL approach was evaluated on *A. niger* and *A. nidulans* genomes [37]. These diverse dataset sizes reflect the maturity and intended applications of different tools, from focused validation studies to large-scale genome mining initiatives.

### 5.7. Challenges and Future Directions

Despite remarkable progress, AI-driven genome mining faces several ongoing challenges. The detection of novel BGC classes that differ substantially from training data remains difficult, as most tools perform best on BGC types well-represented in databases like MIBiG. The accurate prediction of BGC boundaries, particularly for complex or hybrid clusters, continues to require refinement, as evidenced by the substantial improvements achieved through RL approaches [37]. Integration of metagenomic data presents additional challenges due to assembly fragmentation, though ensemble approaches like HiFiBGC have demonstrated significant improvements [61].

Future directions include development of more sophisticated models that can predict not only BGC presence and class but also detailed product structures and bioactivities directly from sequence data. Integration of multiple data modalities—genomics, transcriptomics, metabolomics, and MS—promises to provide more comprehensive characterization of biosynthetic pathways. The application of foundation models and transfer learning approaches may enable better generalization to novel BGC types and organisms. Ultimately, the continued development of AI-driven genome mining tools, coupled with streamlined experimental validation workflows, will accelerate the discovery of novel antibacterial compounds from the vast reservoir of microbial biosynthetic potential.

## 6. ML for Structure Prediction and Activity Screening

The integration of AI and ML into antibacterial drug discovery has fundamentally transformed the paradigm of structure prediction and activity screening. Traditional high-throughput screening campaigns typically achieve hit rates below 1%, requiring extensive resources to identify promising lead compounds. AI-driven approaches have demonstrated the capacity to dramatically improve these hit rates while simultaneously reducing screening costs and accelerating the discovery timeline. ML models trained on large-scale bioactivity datasets can learn complex structure–activity relationships that enable accurate prediction of antibacterial activity, drug–target interactions, and molecular properties from chemical structures alone.

The application of AI to structure prediction and activity screening encompasses diverse methodologies, from classical ML algorithms such as RFs and SVMs to sophisticated DL architectures including CNNs, recurrent neural networks, GNNs, and transformer models. These approaches have been successfully applied across the antibacterial discovery pipeline, from initial virtual screening of ultra-large chemical libraries to prediction of minimum inhibitory concentrations (MICs) and identification of novel scaffolds with activity against priority pathogens. The integration of AI predictions with experimental validation has yielded numerous success stories, with several studies reporting discovery of sub-micromolar antibacterial compounds and validated hits against multidrug-resistant ESKAPE pathogens.

### 6.1. DL Architectures for Molecular Property Prediction

#### 6.1.1. Attention-Based DL Models

**Figure 8:**
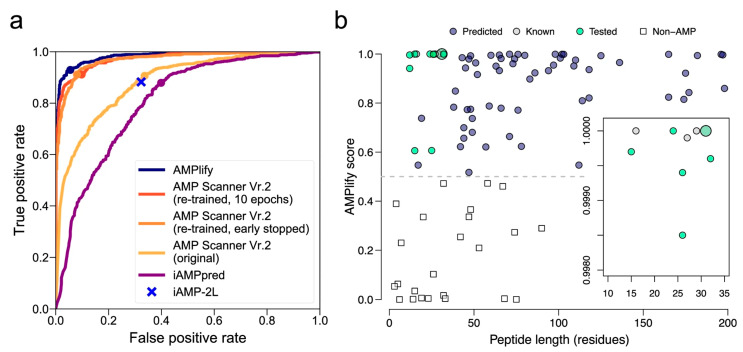
Visualization of AMPlify model performance and the AMP discovery pipeline application results. (**a**) ROC curves are shown for AMPlify and comparator tools, with round dots indicating performance at the 0.5 threshold. The iAMP-2L online server is represented as a single point, as it only provides AMP/non-AMP labels without corresponding probability scores. (**b**) AMPlify prediction scores are plotted against the peptide lengths of 101 sequences analyzed by AMPlify. The grey dotted line marks the 0.5 score threshold used to differentiate AMPs from non-AMPs. The inset provides a magnified view of the upper left region to better visualize the majority of the selected sequences. Reproduced from [67].

Attention mechanisms have emerged as powerful tools for molecular property prediction, enabling models to focus on the most relevant structural features for antibacterial activity. AMPlify, an attentive DL model for antimicrobial peptide (AMP) discovery, exemplifies this approach through its integration of bidirectional LSTM) layers with multi-head scaled dot-product attention (MHSDPA) and context attention layers [67]. The AMPlify ensemble model achieved exceptional performance metrics: 93.71% accuracy, 92.93% sensitivity, 94.49% specificity, 93.66% F1 score, and 98.37% AUROC on a dataset of 3338 training and 835 test sequences (Figure 8). When applied to mining the North American bullfrog genome, AMPlify identified 101 candidate mature sequences, from which 16 peptides were synthesized and tested experimentally [67].

The experimental validation of AMPlify predictions demonstrated substantial practical impact. Four novel AMPs (RaCa-1, RaCa-2, RaCa-3, and RaCa-7) exhibited activity against multiple bacterial species, including WHO priority pathogens and MDR strains. RaCa-1 showed antibacterial activity against all *E. coli* strains tested with MIC values of 10 to 39 μM, while RaCa-2 demonstrated robust bactericidal action against *S. aureus* (MIC = 1 to 2 μM) and *S. pyogenes* (MIC = 25 to 49 μM). RaCa-3 inhibited all bacterial strains tested, and RaCa-7 was active against all *E. coli* strains with MIC values of 6 to 44 μM. This represents a 25% hit rate (4 of 16 tested peptides), substantially higher than typical screening campaigns [67].

#### 6.1.2. Recurrent Neural Networks and Message-Passing Architectures

**Figure 9:**
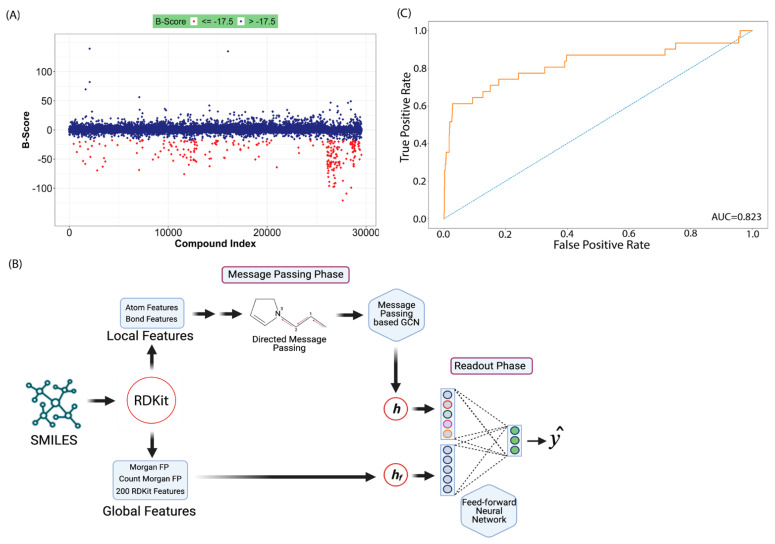
Initial training and performance evaluation of the ML model. (**A**) High-throughput screening was conducted on a compound library comprising 29,537 compounds tested against *B. cenocepacia* K56-2 wild-type. Using a B-score threshold of ≤−17.5, the screen identified 256 active compounds. Inactive compounds are shown in dark blue, while active compounds are highlighted in red. (**B**) The ML model was trained using a D-MPNN approach, which captures local features of compounds, including atom and bond characteristics. To enhance predictive accuracy, the model was also provided with over 200 additional global molecular descriptors. The dataset was partitioned into training, validation, and test sets using an 80:10:10 ratio. (**C**) An AUROC plot is presented to evaluate model performance following training. Reproduced from [68].

Recurrent neural networks and message-passing neural networks excel at capturing sequential and graph-based molecular representations. A directed-message-passing neural network (D-MPNN) trained on high-throughput antibacterial screening data against *Burkholderia cenocepacia* achieved an AUROC score of 0.823 on the test set [68]. The initial experimental screen of 29,537 compounds yielded a hit rate of only 0.87%, typical of unguided high-throughput screening (Figure 9). However, when the trained D-MPNN model was applied to virtual screening of 1614 Food and Drug Administration approved compounds and 224,205 bio-based natural products, experimental validation of top-ranked predictions achieved hit rates of 26% and 12%, respectively—representing at least a 14-fold improvement over the initial screen. Remarkably, over 51% of the predicted antibacterial bio-based natural compounds inhibited ESKAPE pathogens, demonstrating the model’s ability to generalize beyond the specific training organism [68].

### 6.2. GNNs for Drug-Target Interaction Prediction

The AMPredictor, a GCN-based regression model for antimicrobial peptide activity prediction, integrates ESM for node features and predicted contact maps as graph edges, supplemented with Morgan fingerprints and a three-layer fully connected network [69]. Trained on 3280 antimicrobial peptide sequences divided into 8:1:1 training/validation/test sets, AMPredictor achieved a low root mean squared error (RMSE) of 0.5348 and high Pearson’s correlation coefficient (PCC) of 0.7072 on the test set. The model substantially outperformed baseline architectures including MLP, CNN, GRU, LSTM, and Transformer. When integrated with a GAN generator for de novo peptide design, the framework generated 104 novel peptides, with 26 predicted to have MICs below 5 μM [69].

### 6.3. Transformer Models and NLP Approaches

#### 6.3.1. Transformer Architectures for Molecular Representation

Transformer models have emerged as powerful tools for learning molecular representations by treating chemical structures as sequences. The Intermolecular Graph Transformer employs a three-way transformer-based architecture with a dedicated attention mechanism to model intermolecular information for binding activity and binding pose prediction [70]. Intermolecular Graph Transformer outperformed state-of-the-art approaches by 9.1% for binding activity prediction and 20.5% for binding pose prediction. Applied to drug screening against SARS-CoV-2, Intermolecular Graph Transformer identified 83.1% active drugs that were subsequently validated by wet-lab experiments, with predicted binding poses near-native [70]. This exceptional validation rate demonstrates the accuracy of transformer-based approaches for both activity prediction and structural modeling.

The Principal Neighborhood Aggregation-Transformer architecture, combining Principal Neighborhood Aggregation with transformer mechanisms, was applied to serial virtual screening for DYRK1A inhibitor discovery [71]. The hybrid architecture captured complex structure–activity relationships across multiple feature sets, identifying three hit compounds with IC_50_ values below 500 nM [71]. This demonstrates the capability of transformer models to learn subtle structure–activity relationship (SAR) patterns that enable identification of nanomolar inhibitors through computational screening.

#### 6.3.2. Pre-Trained Molecular Representations

**Figure 10:**
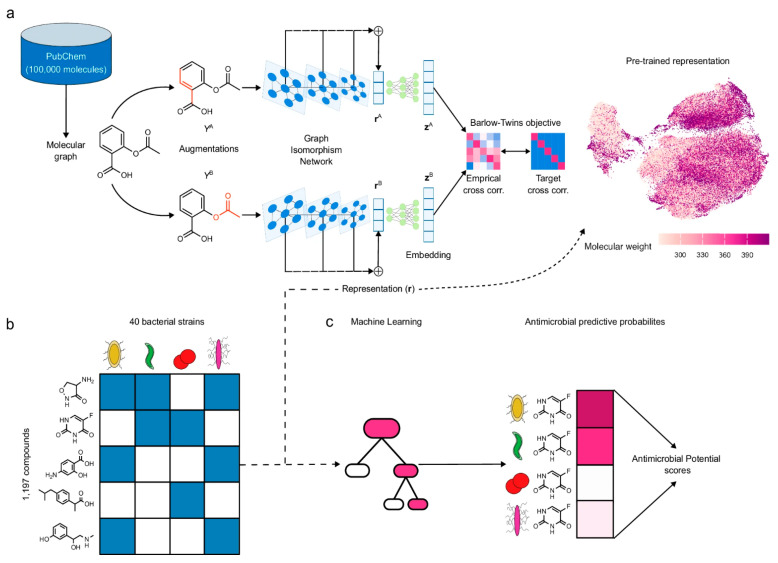
(**a**) The MolE pre-training framework leverages a set of 100,000 unlabeled structures obtained from PubChem to develop a task-independent representation of molecules. (**b**) A predictive model is trained using publicly available growth inhibition data measured against 40 different microbial strains. (**c**) By integrating the pre-trained molecular representation with compound-microbe activity data, a ML model is trained to generate a probability for each compound–microbe pair. This probability reflects the likelihood that the compound inhibits microbial growth. These probabilities are then used to compute Antimicrobial Potential scores, which help prioritize compounds for subsequent experimental validation. Reproduced from [72].

Self-supervised pre-training on large unlabeled molecular datasets has enabled development of general-purpose molecular representations. MolE (Molecular representation through redundancy reduced Embedding), a self-supervised DL framework, leverages unlabeled chemical structures to learn task-independent molecular representations [72]. By combining MolE representation learning with experimental antibacterial screening data, researchers developed a general predictive model to assess antimicrobial potential (Figure 10). The model correctly identified recent growth-inhibitory compounds and discovered de novo three human-targeted drugs as growth inhibitors of *S. aureus*, which were subsequently experimentally confirmed. This lightweight computational strategy offers a viable, cost-effective approach to accelerate antibiotic discovery through transfer learning from pre-trained representations [72].

### 6.4. Ultra-Large-Scale Virtual Screening

**Figure 11:**
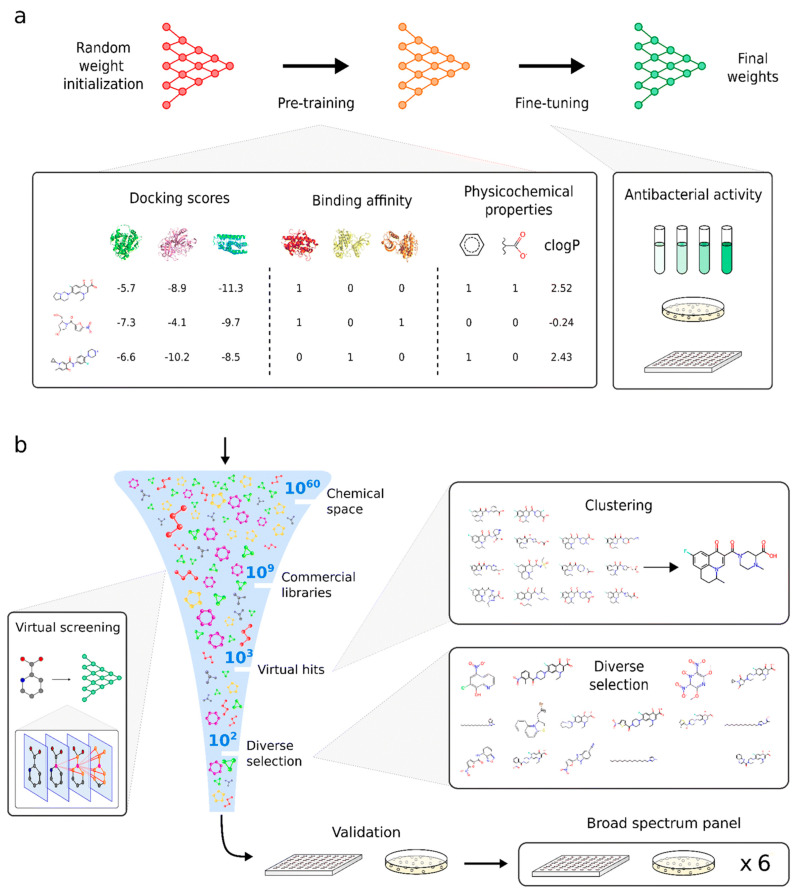
Workflows for transfer learning (**a**) and virtual screening of large chemical libraries (**b**). Prior to fine-tuning on antibacterial data, GNNs underwent pre-training using diverse molecular data, including docking scores, binding affinity, and physicochemical properties. During the virtual screening process, molecules from extensive libraries were ranked based on their predicted inhibition potential. Clustering techniques were then applied to ensure a diverse selection of candidates, thereby increasing the likelihood of identifying structurally novel hits. Selected candidates were initially validated by assessing their antibacterial activity against a single bacterial strain. Those demonstrating activity were subsequently evaluated against a broader panel of both Gram-positive and Gram-negative bacteria. Reproduced from [73].

Transfer learning has enabled virtual screening at unprecedented scales by leveraging knowledge from large pre-training datasets. A transfer learning framework using deep GNNs pre-trained on large molecular datasets and fine-tuned on limited antibacterial screening data achieved remarkable success in identifying antibacterials for ESKAPE pathogens (Figure 11) [73]. The approach virtually screened over 1 billion compounds from ChemDiv and Enamine libraries, prioritizing 156 candidates for experimental testing. Experimental validation against *E. coli* revealed that 54% of tested compounds exhibited antibacterial activity with MIC values ≤ 10 μg/mL, with several demonstrating sub-micromolar potency. These hits showed broad-spectrum efficacy against both Gram-positive and Gram-negative pathogens, including three ESKAPE species. Of 18 broad-spectrum candidates, 15 exhibited minimal cytotoxicity and no hemolytic activity, representing promising leads for further development. The 54% hit rate represents a dramatic improvement over traditional screening and demonstrates the power of transfer learning for ultra-large-scale virtual screening [73].

### 6.5. Classical ML Approaches: RF and Ensemble Methods

Despite the rise of DL, classical ML methods remain competitive for many applications, particularly when training data is limited or interpretability is prioritized. A RF classifier trained on 226 active compounds and 2550 inert compounds for BacA-targeted anti-biofilm drug discovery achieved 96% accuracy with an MCC of 0.93 [74]. The RF model outperformed KNN, SVM, and naive Bayes classifiers. Applied to virtual screening of 9000 phytochemicals, the model identified 300 potentially active compounds, of which 192 exhibited drug-like properties. Molecular docking studies identified Ergotamine, Withanolide E, and DOPPA as top inhibitors with binding affinities of −8.8, −8.1, and −7.9 kcal/mol, respectively. Molecular dynamics simulations over 100 ns confirmed the stability of the BacA-Withanolide E complex, with MMGBSA analysis revealing favorable binding energy profiles dominated by van der Waals interactions [74].

### 6.6. Integration of AI with Experimental Validation

#### 6.6.1. Cell Painting and Morphological Profiling

**Figure 12:**
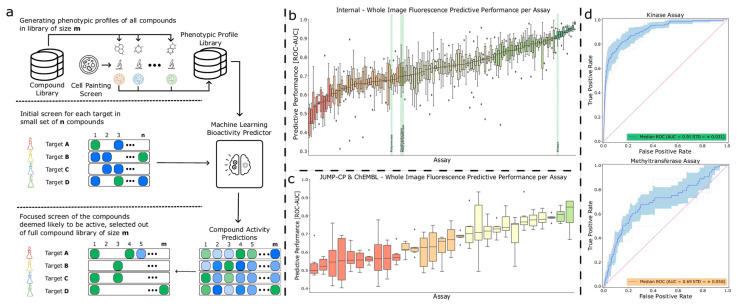
(**a**) The proposed approach leveraging phenotypic screening for bioactivity prediction (predicted active compounds in green, predicted inactive in blue). (**b**) Boxplot illustrating the AUROC performance of each assay across cross-validation test splits (n = 6 splits), ordered by median AUROC. (**c**) Boxplot displaying the AUROC performance of each assay across cross-validation test splits (n = 6 splits) for the JUMP-CP dataset, ranked by median AUROC. (**d**) ROC curves for two representative assays. Reproduced from [75].

The integration of morphological profiling with ML has emerged as a powerful approach for bioactivity prediction (Figure 12). DL models trained on Cell Painting data combined with single-concentration activity readouts achieved an average AUROC of 0.744 ± 0.108 across 140 diverse assays [75]. Performance analysis revealed that 62% of assays reached AUROC ≥0.7, 30% achieved ≥0.8, and 7% exceeded 0.9. Experimental validation confirmed enrichment of active compounds, demonstrating that models trained on Cell Painting data can reliably predict compound activity across diverse targets while maintaining high hit rates and scaffold diversity. This approach enables smaller, more focused compound screens, reducing screening campaign size and saving substantial time and resources [75].

#### 6.6.2. Fusion of Docking Scores with DL

The integration of traditional computational methods with DL has yielded improved screening performance. A fusion strategy multiplying Watvina docking scores by CNNscore (pose scores from GNINA’s CNN) demonstrated state-of-the-art screening power across diverse validation datasets [76]. Applied to virtual screening for TYK2 inhibitors, this docking-score fusion technique identified two promising hits with measured IC values, demonstrating practical improvement in virtual screening workflows [76]. The success of this hybrid approach highlights the complementary strengths of physics-based and data-driven methods.

### 6.7. Generative AI for De Novo Compound Design

#### 6.7.1. Generative Adversarial Networks for Peptide Design

Generative adversarial networks (GANs) have enabled de novo design of antimicrobial peptides with predicted activity. A GAN-based framework employing a three-layer 2D convolution architecture with amino acid factor encoding was coupled with the AMPredictor GCN-based regression model for activity prediction [69]. The GAN generated sequences with approximately 0.35 identity to known databases, ensuring novelty. From 104 generated novel peptides, 26 were predicted to have MICs below 5 μM. Experimental validation of three peptides (P001, P002, P076) confirmed their bifunctional antimicrobial activity [69]. P076 demonstrated potent bactericidal activity with MIC of 0.21 μM against MDR *A. baumannii*, binding to lipid A with dissociation constant (Kd) of 56.1 nM [69]. In vivo studies showed that P076 reduced bacterial loads in mouse peritoneal lavage fluid and spleen. P002 exhibited broad antiviral activity, inhibiting five enveloped viruses with EC_50_ values ranging from 0.37 μM (against CHIKV) to 2.08 μM (against HTNV). P001 bound to lipid A with Kd of 65.7 nM [69]. These results demonstrate the capability of generative AI to design novel AMPs with potent, validated activity against priority pathogens.

#### 6.7.2. Reconstruction-Based Approaches for Chemical Space Exploration

Novel approaches combining molecular property prediction with molecular reconstruction have enabled exploration beyond charted chemical space. A joint modeling approach utilizing ‘unfamiliarity,’ a reconstruction-based metric, effectively identifies out-of-distribution molecules and serves as a reliable predictor of classifier performance [55]. Systematic analysis spanning more than 30 bioactivity datasets demonstrated that unfamiliarity-based molecule screening can discover diverse and structurally novel compounds [55]. Experimental validation for two clinically relevant kinases discovered seven compounds with low micromolar potency that showed limited similarity to training molecules [55]. This approach addresses a critical limitation of ML models—their tendency to perform poorly on molecules dissimilar to training data—by explicitly quantifying and leveraging unfamiliarity to guide exploration of novel chemical space [55].

### 6.8. Comparative Analysis of AI Methodologies

#### 6.8.1. Performance Across Different Architectures

Systematic comparisons of AI architectures reveal important insights for method selection. The comparison of nine models for activity prediction demonstrated that co-representation models (FP-GNN, HiGNN, FG-BERT) generally outperformed pure fingerprint-based (RF::Morgan, XGBoost::Morgan) or pure graph-based (GCN, GAT, MPNN, Attentive FP) approaches, with FP-GNN achieving the highest AUROC of 0.884 [77]. However, the analysis revealed dataset size-dependent performance: fingerprint-based ML models outperformed graph-based DL models on large datasets (>1000 compounds), while graph-based models showed advantages on smaller datasets [77]. This finding provides practical guidance for method selection based on available training data.

For AMP activity prediction, the GCN-based AMPredictor (RMSE: 0.5348, PCC: 0.7072) substantially outperformed baseline architectures including MLP, CNN, GRU, LSTM, and Transformer [69]. The superior performance of GCN suggests that explicit modeling of the molecular graph structure provides advantages over sequence-based representations for peptide activity prediction. In contrast, for small molecule activity prediction, the attention-based AMPlify model (93.71% accuracy, 98.37% AUROC) outperformed RF, SVMs, and earlier DL models including iAMP-2L, iAMPpred, Deep-AmPEP30, Deep-ABPpred, and AMP Scanner Vr.2 [67].

#### 6.8.2. Hit-Rate Improvements Across Studies

The impact of AI-guided virtual screening is most clearly demonstrated through hit-rate improvements. The D-MPNN model for antibacterial discovery achieved 14-fold to 30-fold hit rate improvements (from 0.87% to 12 to 26%) when applied to virtual screening of Food and Drug Administration approved drugs and natural products [68]. Transfer learning-based screening of over 1 billion compounds achieved a 54% hit rate for antibacterial activity against *E. coli* [73]. The MTL approach for antileishmanial discovery achieved a 45% hit rate (9 of 20 tested compounds) [78]. These dramatic improvements—from typical hit rates below 1% to rates exceeding 10 to 50%—demonstrate the transformative impact of AI on drug discovery efficiency.

### 6.9. Challenges and Future Directions

Despite remarkable progress, several challenges remain in AI-driven structure prediction and activity screening. Model performance on out-of-distribution molecules remains a critical limitation, as demonstrated by the need for unfamiliarity metrics to identify when predictions may be unreliable [55]. The dataset size-dependent performance of different architectures suggests that no single approach is universally optimal, requiring careful method selection based on available data [77]. Integration of multiple data modalities—including chemical structures, bioactivity data, morphological profiles, and omics data—represents a promising direction for improving prediction accuracy and expanding the scope of AI-driven discovery [75].

The successful examples reviewed here demonstrate that AI-driven structure prediction and activity screening have matured from proof-of-concept studies to practical tools delivering validated antibacterial compounds. The consistent achievement of hit rates exceeding 10 to 50% across diverse studies, combined with discovery of compounds with sub-micromolar to nanomolar potencies against priority pathogens, establishes AI as an essential component of modern antibacterial drug discovery. As models continue to improve and training datasets expand, AI-driven approaches are poised to play an increasingly central role in combating antibacterial resistance through accelerated discovery of novel therapeutic agents.

## 7. Accelerating Dereplication with Computational Tools

The identification and characterization of bio-based natural products from complex biological matrices represents a critical bottleneck in antibacterial drug discovery. Traditional dereplication approaches, which rely on manual interpretation of spectroscopic data and comparison against limited reference libraries, are time-consuming and often fail to identify novel compounds. The integration of AI and computational tools into dereplication workflows has revolutionized this process, enabling rapid, accurate, and scalable identification of known compounds while prioritizing novel structures for further investigation. This section examines the state-of-the-art computational tools and methodologies that are accelerating dereplication in NPD, with particular emphasis on MS-based identification, NMR prediction, chemoinformatic databases, DL approaches, and integrated multi-analytical workflows.

### 7.1. MS-Based Identification Tools

#### 7.1.1. Molecular Networking and GNPS

**Figure 13:**
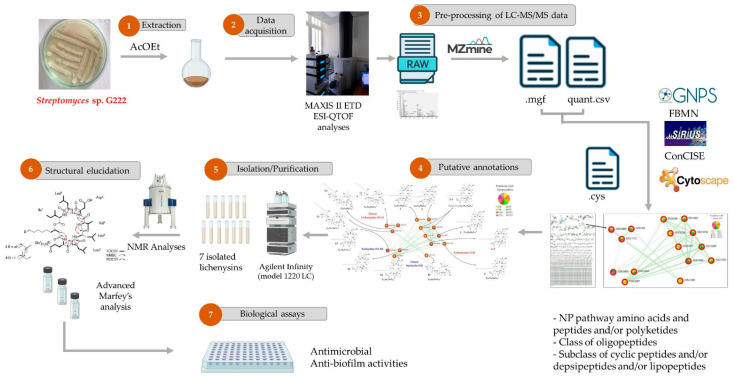
Integrated workflow for the discovery and characterization of lichenysins from *Streptomyces* sp. G222. Reproduced from [79].

The GNPS platform has emerged as a cornerstone tool for dereplication, enabling researchers to visualize and annotate complex metabolomic datasets through spectral similarity networks. Feature-Based Molecular Networking (FBMN) has been successfully applied across diverse NPD projects, demonstrating its versatility in mapping chemical diversity. In a study [79] of marine-derived *Streptomyces* strain G222 (Figure 13), GNPS molecular networking combined with spectral library matching enabled the putative annotation of cyclic and linear lipopeptides from the lichenysin and surfactin families, leading to the isolation and NMR confirmation of five lichenysins (3–7) with anti-biofilm activity against *P. aeruginosa* at 100 µM and selective antibacterial activity against MRSA [79].

The application of FBMN to algal research has demonstrated the power of molecular networking for dereplication of specialized metabolite classes. When applied to 33 crude algal extracts using UHPLC-HRMS/MS, FBMN successfully identified and dereplicated mycosporine-like amino acids, with structures corroborated through integration of in-house and open-source spectral libraries on the GNPS platform [80]. Similarly, molecular networking facilitated the identification of budmunchiamines (macrocyclic alkaloids) from *Acacia senegal* fractions that exhibited synergistic activity with chloramphenicol against *E. coli* resistant strains producing AcrB efflux pumps [81].

Advanced molecular networking approaches have extended beyond traditional spectral matching. Anchor-Based Molecular Networking combined with orthogonal chromatography (reversed-phase liquid chromatography and hydrophilic interaction liquid chromatography (HILIC)) achieved comprehensive coverage of new psychoactive substances, with Protocol P1 recovering 90% of analytes and enabling MS/MS acquisition for 100% of compounds at 50 ng/mL, 97% at 5 ng/mL, and 42% at 0.5 ng/mL, with detection limits down to 1 ng/mL [82].

#### 7.1.2. SIRIUS and CSI:FingerID

**Figure 14:**
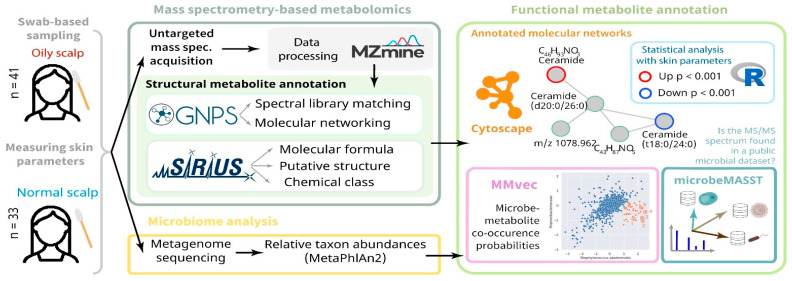
Study design and multi-omics analysis workflow integrating untargeted metabolomics with metagenomic sequencing. Multi-omics data integration was performed using MMvec, with the resulting associations visualized as a microbe–metabolite network. Reproduced from [83].

SIRIUS has become an indispensable tool for molecular formula prediction and structure elucidation from tandem MS data. The integration of SIRIUS with CSI:FingerID for structure prediction has demonstrated remarkable performance across multiple studies. In a multi-omics study of human skin microbiome (Figure 14), SIRIUS expanded annotation coverage by 23.5-fold for C18 positive mode, 45-fold for C18 negative mode, 7.6-fold for HILIC positive mode, and 13.5-fold for HILIC negative mode compared to spectral library matching alone [83]. The molecular formula predictions from SIRIUS matched spectral library annotations with accuracies of 66%, 70%, 90%, and 95% across different datasets, while chemical class prediction accuracy reached 84 to 92% at the superclass level, 80 to 90% at the class level, and 57 to 86% at the subclass level for positive ionization mode [83].

**Figure 15:**
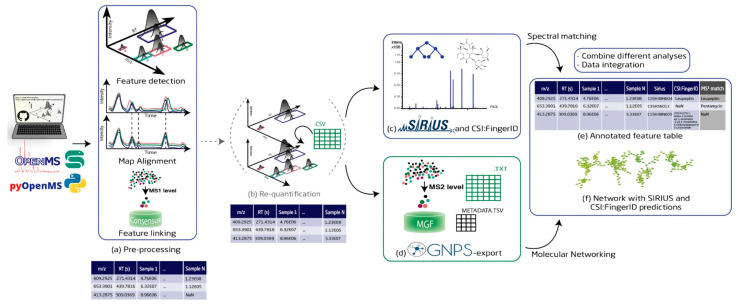
Overview of UmetaFlow. (**a**) The pre-processing step comprises a series of algorithms that convert raw data into a table of metabolic features. A key algorithm in this stage is feature detection, which identifies mass traces, deconvolutes them, and assembles individual isotopic mass traces into metabolite features. Map alignment corrects for retention time shifts, and feature linking connects corresponding features across multiple runs. (**b**) Immediately following pre-processing, an optional re-quantification step can be selected to address features with missing values. (**c**) The resulting feature files (whether re-quantified or not), along with the corresponding mzML files, are fed into the SIRIUS executable for molecular formula and structural predictions. (**d**) The clustered feature files and mzML files are passed to the GNPSexport algorithm, which generates all necessary files for FBMN or Ion Identity Molecular Networking. (**e**,**f**) The final outputs of UmetaFlow include a feature matrix and a GraphML network file. These contain MS^2^ library matches, as well as formula and structural prediction annotations. Reproduced from [84].

The UmetaFlow workflow (Figure 15), which integrates SIRIUS and CSI:FingerID with OpenMS algorithms, demonstrated that SIRIUS accurately predicted 76% of molecular formulas, while CSI:FingerID achieved approximately 62% accuracy for structure predictions in actinomycete-derived secondary metabolites [84]. When validated on publicly available datasets MTBLS733 and MTBLS736, UmetaFlow detected more than 90% of ground truth features, with accurate quantification of 94.6% of features and successful identification of 47 true discriminating markers with only 5 false positives for MTBLS733. For MTBLS736, the workflow annotated 874 out of 970 features (90.1% true feature identification rate) and quantified 81.7% accurately [84].

The Metabolome Annotation Workflow (MAW) demonstrated the complementary nature of SIRIUS-based predictions and spectral library matching [85]. In a standards dataset from hypersalinity studies in diatoms, MAW correctly identified 6 of 9 standard metabolites as top candidates, including acetyl-L-carnitine, butanoyl-L-carnitine, methionine sulfoxide, pipecolic acid, propanoyl-L-carnitine, and N,N-dimethyl arginine. The workflow achieved 97.3% annotation of features with chemical class and 100% with molecular formula prediction, with SIRIUS providing 70.2% total structural annotations compared to 75.7% from spectral library matching. In a bryophyte dataset, MAW annotated 881 out of 933 features with chemical structures, including 4 marchantin compounds and 1 perrottetin compound [85].

#### 7.1.3. MetFrag and MS-FINDER

MetFrag has been integrated into multiple dereplication workflows as a complementary tool for structure elucidation. In the MAW workflow, MetFrag searching against PubChem achieved 32.4% total structural annotations (12 features), demonstrating its utility when combined with other annotation methods [85]. The Cross-Modal Compound Identification from MS/MS Spectra to Molecular Structures (CSU-MS^2^) framework, which employs cross-modal contrastive learning, demonstrated that MetFrag achieved a Recall@1 of 48.59% when matching 1047 spectra against 1,001,047 compounds, though this was surpassed by more advanced DL approaches [86].

#### 7.1.4. Integrated MS-Based Platforms

The integration of multiple MS-based tools has proven essential for comprehensive dereplication. An integrated platform coupling chromatography with MS and computer-aided annotation using MS-DIAL and SIRIUS achieved detection limits of 0.03 µg/g for dimethoate impurities, successfully identifying 3 known impurities and 15 novel structures through spectral deconvolution, sub-ppm molecular formula prediction, and fragmentation modeling [87].

**Figure 16:**
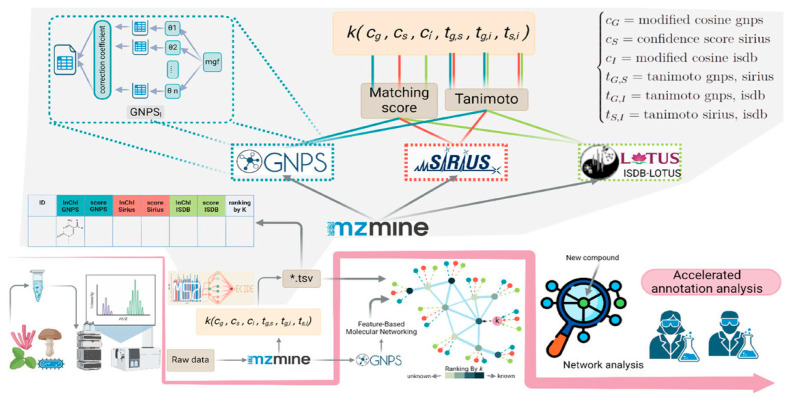
Overview of the MS2DECIDE workflow and its integration into NPD. Reproduced from [88].

The MS2DECIDE platform represents a significant advancement in aggregating outputs from multiple annotation tools (Figure 16). By leveraging decision theory and expert knowledge to combine outputs from GNPS, SIRIUS, and ISDB-LOTUS, MS2DECIDE computes recommendations for targeting bio-based natural products based on their potential novelty, reliably capturing the novelty of bio-based natural products from tandem mass spectra [88].

### 7.2. DL Approaches for Spectral Prediction and Matching

#### 7.2.1. Transformer-Based Models for Mass Spectra

The emergence of transformer-based neural networks pre-trained on large-scale spectral repositories has marked a paradigm shift in computational dereplication. Deep Representations Empowering the Annotation of Mass Spectra (DreaMS), a foundation model pre-trained on millions of unannotated tandem mass spectra from the GNPS Experimental Mass Spectra (GeMS) dataset mined from the MassIVE GNPS repository, has achieved state-of-the-art performance across diverse spectrum interpretation tasks [89]. The model learns rich molecular representations through self-supervised learning by predicting masked spectral peaks and chromatographic retention orders. The DreaMS Atlas, a molecular network constructed using DreaMS annotations, encompasses 201 million MS/MS spectra, representing an unprecedented resource for spectral similarity searches and compound identification [89].

#### 7.2.2. DL for Fragment and Spectral Prediction

**Figure 17:**
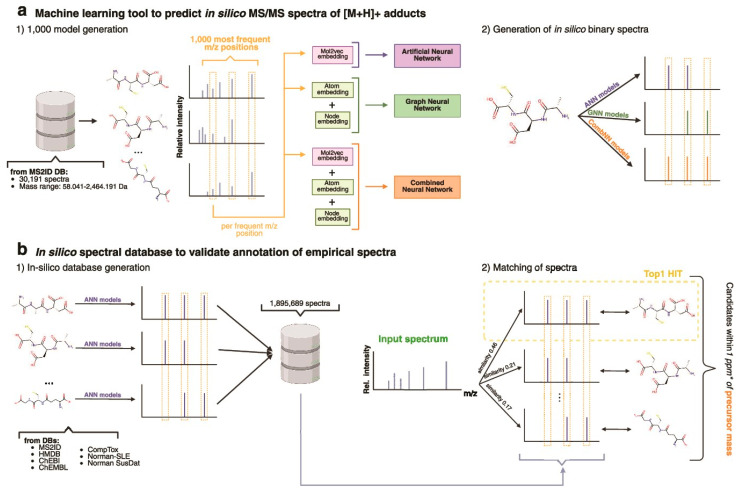
SingleFrag for spectral prediction and annotation. (**a**) Using an empirical database of MS/MS spectra, three distinct model types were trained to predict the presence or absence of individual fragment ions across spectra. Specifically, 1000 models were developed for each of the three model types, corresponding to the 1000 most frequently observed fragment ions. After training is complete, the full spectrum for a given molecule is generated by independently predicting each of these 1000 peaks. (**b**) For annotating unknown empirical spectra, a database containing in silico predicted spectra for more than 1.8 million compounds was constructed. When annotating an unknown empirical spectrum, all candidates from the database with masses matching the unknown spectrum are retrieved. These candidates are then ranked based on the similarity between their predicted spectra and the target empirical spectrum. Reproduced from [90].

SingleFrag (Figure 17), a novel DL tool that predicts individual MS/MS fragments separately rather than attempting to predict entire fragmentation spectra at once, has demonstrated superior performance compared to state-of-the-art in silico fragmentation tools for metabolite annotation. As proof of concept, SingleFrag successfully identified three previously unidentified compounds frequently found in human samples, demonstrating its utility for annotating unknown MS/MS spectra of known compounds [90].

The NPS-MS DL method, trained by transfer learning from a generic MS/MS prediction model, provides MS/MS spectra prediction and identification capabilities against a database of approximately 8.7 million predicted new psychoactive substance compounds from DarkNPS and 24.5 million predicted ESI-QToF-MS/MS spectra. This approach successfully identified a novel derivative of phencyclidine within an unknown powder seized in Denmark without requiring reference standards, demonstrating the power of DL for identifying compounds absent from spectral libraries [91].

#### 7.2.3. Formula Prediction with DL

**Figure 18:**
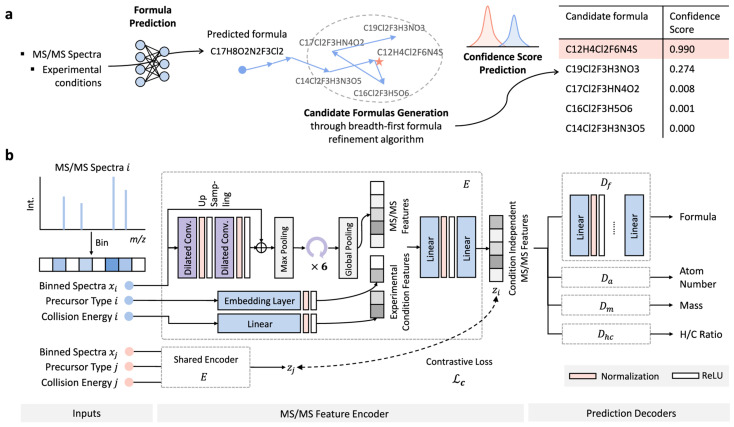
Formula identification from tandem mass spectra using DL. (**a**) The FIDDLE workflow consists of three primary stages: predicting molecular formulas from MS/MS spectra using a DL model; generating candidate formulas via a breadth-first formula refinement algorithm; and assigning confidence scores to the resulting candidate formulas. (**b**) The DL model architecture features an MS/MS spectrum encoder (E) alongside multiple decoders (D_f_, D_a_, D_m_, and D_hc_), which output the predicted formula and auxiliary variables, including atom counts, molecular mass, and H/C ratio. A contrastive loss is calculated on pairs of condition-independent MS/MS features (z_i_ and z_j_) to support model convergence. Reproduced from [92].

FIDDLE (Formula IDentification by Deep LEarning), trained on over 38,000 molecules and 1 million MS/MS spectra from various Q-TOF and Orbitrap instruments, accelerates formula identification by more than 10-fold compared to conventional methods (Figure 18) [92]. FIDDLE achieves top 1 and top 5 accuracies of 88.3% and 93.6%, respectively, outperforming state-of-the-art methods based on top–down (SIRIUS) and bottom-up (BUDDY) approaches by over 10%. On external metabolomics datasets, FIDDLE achieves top 5 accuracies of 75.1% in positive ion mode and 66.2% in negative ion mode, with further improvements to 80.0% and 73.8% when combined with SIRIUS and BUDDY [92].

#### 7.2.4. Cross-Modal Contrastive Learning

CSU-MS^2^ employs a contrastive learning framework with an External Space Attention Aggregation module to bridge the gap between spectral data and molecular structures [86]. When validated on three external datasets (MTBLS265 for human metabolomics, PMhub for plant metabolites, and CASMI 2022 challenge), CSU-MS^2^ achieved a Recall@1 of 75.45% matching 1047 spectra against 1,001,047 compounds from a Spectrum-searchable Structural Feature Database (SSFDB) assembled from 23 structural databases. This significantly surpassed CFM-ID (68.38%), SIRIUS (64.85%), MetFrag (48.59%), and CMSSP (30.47%). Domain-specific retrieval demonstrated even higher performance, with a Recall@10 of 91.67% for blood metabolites [86].

#### 7.2.5. De Novo Structure Generation

**Figure 19:**
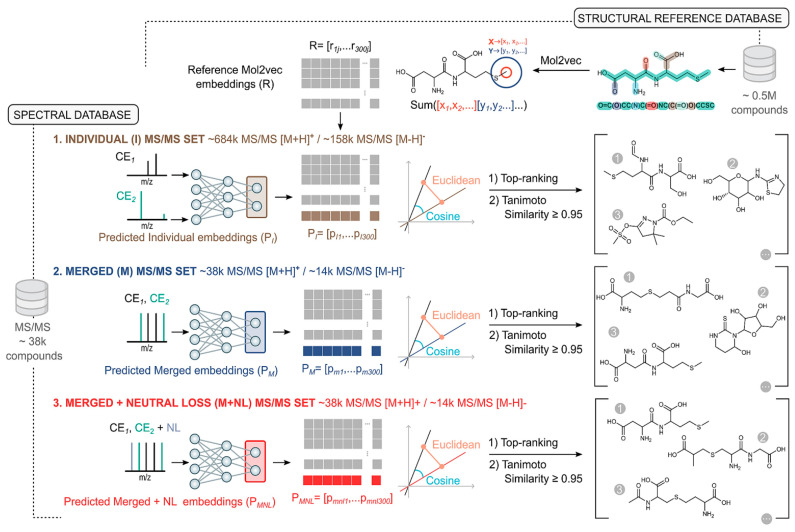
Computational workflow for molecular identification using CNN-generated embeddings. MS/MS spectra are fed into a CNN trained to produce 300-dimensional embeddings that align with Mol2vec representations. The predicted embeddings are then matched against a reference database through a multi-step process: (**1**) Precursor mass filtering: candidate molecules are excluded if their precursor ion masses fall outside a tolerance of ±0.001 Da. (**2**) Similarity calculations: both Euclidean distance and cosine similarity are computed between the predicted embeddings and reference Mol2vec embeddings, with molecules ranked accordingly. (**3**) Structural validation: the top-ranked candidates undergo further evaluation using the Tanimoto score to confirm structural alignment between the predicted and reference molecules. Reproduced from [93].

ChemEmbed (Figure 19), a DL framework utilizing enhanced MS/MS data merged across multiple collision energies and incorporating calculated neutral losses from 38,472 distinct compounds, ranks the correct candidate first in over 42% of cases and within the top 5 in more than 76% of cases. When applied to the Annotated Recurrent Unidentified Spectra dataset, ChemEmbed confirmed 25 previously unidentified compounds and outperformed SIRIUS 6 in external benchmarks such as CASMI 2016 and 2022 [93].

Spec2Mol, an end-to-end DL architecture inspired by Speech2Text models, translates mass spectra to de novo molecular structures using an encoder–decoder architecture [94]. The encoder learns spectral embeddings while the decoder, pre-trained on 135 million molecules from PubChem and ZINC-12, reconstructs SMILES sequences of recommended chemical structures. Evaluated on the NIST Tandem Mass Spectral Library 2020 containing over 1 million spectra from more than 30,000 compounds (including 6000 human metabolites, 8000 plant metabolites, and 20% drugs), Spec2Mol achieved autoencoder reconstruction accuracy of 93.3% for NIST molecules and 94.95% on held-out test sets. The framework identified exact structures for 7% of test cases and exact molecular formulas for 26%, with maximum common substructure metrics showing that the common substructure represents nearly 70% of the reference structure for the closest prediction and over 50% for average predictions [94].

MS2SMILES, which treats hydrogen atoms as implicitly linked to heavy atoms and incorporates SMILES grammatical rules, achieved SMILES prediction accuracies of 53.6% on the GNPS dataset and 63.8% on the CASMI 2016 dataset, representing improvements of 19.9% and 10.9% compared to the leading method at the time [95].

#### 7.2.6. Domain-Inspired Neural Architectures

Metabolite Inference with Spectrum Transformers (MIST) incorporates domain insights into its neural architecture by forcing the network to more directly link peaks to physical atom representations, neutral losses, and chemical substructures [96]. MIST outperforms both standard neural architectures and state-of-the-art kernel methods on fingerprint prediction from spectra for over 70% of metabolite standards, retrieving over 66% of metabolites with equal or improved accuracy, with 29% showing strictly better performance. In a prospective application to an inflammatory bowel disease patient cohort, MIST successfully identified new differentially abundant metabolite structures and subsequently annotated dipeptides and alkaloid compounds without requiring spectral standards [96].

### 7.3. Chemoinformatic Databases and Resources

#### 7.3.1. Spectral Libraries and Bio-Based Natural Product Databases

The availability of comprehensive spectral libraries and bio-based natural product databases is fundamental to computational dereplication. The MassIVE GNPS repository, which served as the source for the GeMS dataset containing millions of unannotated tandem mass spectra, represents one of the largest publicly accessible spectral resources [89]. The microbeMASST database, containing approximately 60,000 taxonomically curated microbial cultures, has enabled researchers to link metabolites to specific microbial producers through spectral matching [83].

The Human Metabolome Database, METLIN, MassBank, mzCloud, and LIPID MAPS Structure Database have been integrated into multiple dereplication workflows, providing reference spectra for known metabolites [94]. The NIST Tandem Mass Spectral Library 2020, containing over 1 million spectra from more than 30,000 compounds, has served as a benchmark dataset for evaluating DL models [94].

#### 7.3.2. Structural Databases

Large-scale structural databases have become essential for in silico spectral prediction and structure matching. PubChem and ZINC-12, collectively containing 135 million molecules, have been used for pre-training DL models for molecular structure recommendation [94]. The COCONUT (COlleCtion of Open Natural prodUcTs) database has been integrated into multiple annotation workflows, providing comprehensive coverage of natural product chemical space [93]. ChEMBL, SuperNatural, and ZINC20 databases have been utilized for training multidimensional molecular embeddings in frameworks such as ChemEmbed [93]. The SSFDB, assembled from 23 structural databases for the CSU-MS^2^ framework, contains 1,001,047 compounds and represents a comprehensive resource for cross-modal compound identification [86].

**Figure 20:**
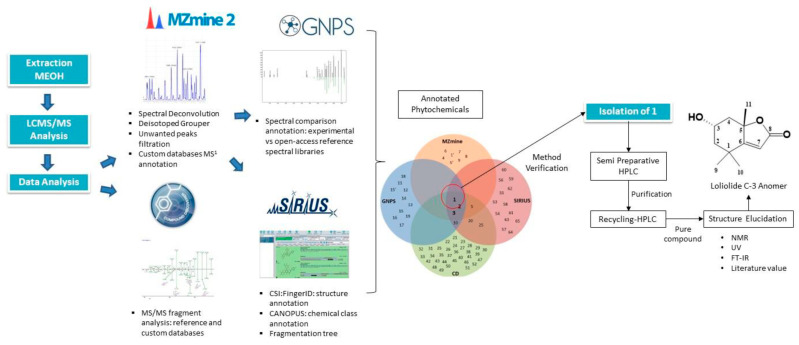
Infographic depicting the workflow for phytochemical annotation and identification from *E. indica* using HPLC-MS/MS analysis. Reproduced from [97].

Database-driven approaches have demonstrated high efficiency in phytochemical annotation. A study of *Eleusine indica* using MZmine, GNPS, Compound Discoverer, and SIRIUS platforms successfully identified 65 phytochemicals comprising primary and secondary metabolites, with structural elucidation verified by isolation and NMR characterization of a 3-OH anomer of loliolide. This approach demonstrates the utility of integrating multiple database resources for rapid and simultaneous identification of phytochemicals (Figure 20) [97].

### 7.4. NMR Prediction and Structure Elucidation Tools

While MS-based tools dominate current computational dereplication workflows, NMR spectroscopy remains essential for definitive structure confirmation. The integration of AI-based NMR prediction tools with MS-based dereplication is an emerging area. Traditional NMR structure elucidation continues to serve as the gold standard for validation, as demonstrated in multiple studies where computational dereplication predictions were confirmed through detailed 1D and 2D NMR spectroscopy (COSY, HSQC, HMBC, TOCSY, ROESY) [79,97].

### 7.5. Integrated Multi-Analytical Workflows

#### 7.5.1. Automated High-Throughput Workflows

UmetaFlow represents a comprehensive automated workflow that integrates data pre-processing, spectral matching, molecular formula and structural predictions, and downstream analysis through GNPS FBMN and Ion Identity Molecular Networking [84]. Implemented as a Snakemake workflow with Python bindings to OpenMS algorithms (pyOpenMS), UmetaFlow provides scalability and reproducibility for large-scale metabolomics studies. When processing 1245 raw files from 100 actinomycete strains, the workflow completed pre-processing in 1 h 12 min 24 s, with formula and structural predictions requiring 9 days 23 h 58 min 16 s. The workflow annotated 1684 features (~3%) with spectral matches, 25,976 features (~46%) with formula predictions, and 13,722 features (~24%) with both spectral matches and formula predictions, achieving Metabolomics Standards Initiative level-three annotations [84].

The MAW provides a reproducible framework implemented in Galaxy, KNIME, and other platforms, combining in silico generated spectral matching with experimental spectral matching for higher confidence annotations [85]. MAW integrates SIRIUS, MetFrag, RDKit, ClassyFire, and PubChemPy to provide comprehensive chemical class annotations, molecular formula predictions, and structural annotations [85].

#### 7.5.2. Multi-Omics Integration

The integration of metabolomics with genomics has been enhanced through computational tools that link BGCs to their metabolic products. NPClassScore, implemented in the NPLinker platform, matches natural product ontologies between BGCs and MS/MS spectra, reducing the number of potential BGC-MS/MS spectrum links by an average of 63% compared to co-occurrence-based strategies alone while retaining 96% of experimentally validated links. This approach was validated on three paired omics datasets totaling 189 bacterial strains, demonstrating its utility in prioritizing plausible candidates for manual inspection [98].

A multi-omics strategy applied to human skin microbiome samples from 74 healthy volunteers integrated metabolomics data processed through MZmine, GNPS, SIRIUS, ZODIAC, CSI:FingerID, and microbeMASST with genomics data processed through Qiime2 [83]. The workflow achieved combined annotation rates of 52% for C18-positive mode, 90% for C18-negative mode, 24% for HILIC-positive mode, and 54% for HILIC-negative mode. MMvec processing reduced 44,570 features to 1225 molecular networks, facilitating the identification of an antagonistic interaction between *Staphylococcus epidermidis* and *Cutibacterium acnes* involving three molecular families [83].

#### 7.5.3. Orthogonal Analytical Techniques

The integration of orthogonal chromatographic modes has proven essential for comprehensive metabolite coverage. The combination of reversed-phase liquid chromatography and HILIC in the Anchor-Based Molecular Networking workflow demonstrated complementary coverage, with Protocol P1 achieving 90% analyte recovery and enabling MS/MS acquisition for 100% of compounds at 50 ng/mL [82]. This dual-mode approach successfully identified structurally related analogues including bromazolam, fluoromethamphetamine, and MDPHP in clinical samples [82].

### 7.6. Performance Benchmarking and Validation

#### 7.6.1. Comparative Tool Performance

Systematic benchmarking across multiple tools has revealed significant performance differences. In the CSU-MS^2^ study, when matching 1047 spectra against 1,001,047 compounds, CSU-MS^2^ achieved Recall@1 of 75.45%, significantly outperforming CFM-ID (68.38%), SIRIUS (64.85%), MetFrag (48.59%), and CMSSP (30.47%) [86]. For formula prediction, FIDDLE demonstrated top 1 accuracy of 88.3% and top 5 accuracy of 93.6%, exceeding SIRIUS and BUDDY by over 10% [92].

#### 7.6.2. Annotation Rate Improvements

The integration of multiple annotation strategies has consistently demonstrated superior performance compared to single-method approaches. In the multi-omics skin microbiome study, spectral library matching alone yielded 2 to 4% annotation rates, increasing to 9 to 10% in analogue mode [83]. The addition of SIRIUS expanded annotation coverage by 23.5-fold to 45-fold depending on the chromatographic mode and ionization polarity. CSI:FingerID further increased network annotation rates by 1.75-fold to 15.5-fold. The combined approach achieved total annotation rates of 52% to 90% depending on the analytical conditions [83].

#### 7.6.3. Time Efficiency and Scalability

Computational dereplication tools have demonstrated substantial time savings compared to manual approaches. FIDDLE accelerates formula identification by more than 10-fold compared to conventional methods [92]. The UmetaFlow workflow, despite requiring approximately 10 days for complete processing of 1245 raw files including formula and structural predictions, enables fully automated analysis that would be impractical to perform manually [84]. The pre-processing step alone, completed in approximately 1 h for 1245 files, demonstrates the scalability of modern computational workflows [84].

### 7.7. Case Studies

#### 7.7.1. Marine Natural Products

The application of computational dereplication to marine-derived *Streptomyces* strain G222 exemplifies the power of integrated approaches [79]. GNPS molecular networking combined with SIRIUS and conCISE (Consensus Annotation Propagation of in silico Elucidations) enabled putative annotation of cyclic and linear lipopeptides from the lichenysin and surfactin families. This computational dereplication strategy guided the isolation of five lichenysins (3–7), which were confirmed by detailed NMR spectroscopy and modified Marfey’s method for absolute configuration determination. The isolated compounds exhibited anti-biofilm activity against *P. aeruginosa* MUC-N1 at 100 µM and selective antibacterial activity against MRSA without affecting bacterial growth curves or causing membranotropic activity. This study represents the first report of combined cyclic and linear lichenysins and surfactins from a Streptomyces strain, compounds previously known only from *Bacillus* species [79].

#### 7.7.2. Fermented Food Metabolomics

FBMN combined with DL was applied to map metabolites in *Lactobacillus plantarum*-fermented sea buckthorn milk, demonstrating the utility of computational dereplication in food science applications. The FBMN-DL pipeline successfully mapped the metabolite landscape of the fermented product, though specific annotation rates were not detailed in the available metadata [99].

#### 7.7.3. Actinomycete Secondary Metabolites

UmetaFlow validation on actinomycete-derived secondary metabolites demonstrated 100% detection of expected features from commercial standards including germicidins A and B, kanamycin, tetracycline hydrochloride, thiostrepton, globomycin, ampicillin, and apramycin [84]. The workflow successfully annotated compounds from *Streptomyces collinus* Tü 365 (kirromycin and desferrioxamine B), *Kutzneria* sp. CA-103260 (epemicins A and B), and *Streptomyces* sp. NBC 00162 (pyracrimycin A), achieving 76% accuracy for molecular formula predictions and 65% for structural annotations [84].

#### 7.7.4. Plant Metabolomics

The application of database-driven approaches to *E. indica* using MZmine, GNPS, Compound Discoverer, and SIRIUS successfully identified 65 phytochemicals, with verification through isolation and NMR characterization of a 3-OH anomer of loliolide. This demonstrates the reliability of computational dereplication for rapid phytochemical profiling when combined with targeted isolation for validation [97].

### 7.8. Challenges and Future Directions

Despite remarkable advances in computational dereplication, several challenges remain. The accuracy of structure prediction from MS/MS data, while improving, still requires experimental validation through NMR spectroscopy or other orthogonal methods. The coverage of spectral libraries and structural databases, though expanding rapidly, remains incomplete for many natural product classes, particularly those from underexplored microbial and marine sources. The integration of AI-based NMR prediction tools into automated dereplication workflows represents an important frontier.

The computational cost of DL approaches, as evidenced by the 10-day processing time for large-scale predictions in UmetaFlow [84], highlights the need for continued optimization of algorithms and computational infrastructure. However, the substantial improvements in accuracy, annotation rates, and throughput compared to manual methods justify these computational investments.

Future developments should focus on: (1) expanding spectral and structural databases to improve coverage of bio-based natural product chemical space, (2) developing more sophisticated DL architectures that better capture the physics and chemistry of fragmentation processes, (3) integrating multiple analytical modalities (MS, NMR, ultraviolet (UV), infrared (IR)) into unified computational frameworks, (4) improving the interpretability of AI-based predictions to facilitate expert validation, and (5) developing standardized benchmarking datasets and metrics to enable rigorous comparison of emerging methods.

The convergence of large-scale spectral repositories, advanced DL architectures, and integrated multi-omics workflows is transforming natural product dereplication from a labor-intensive bottleneck into a rapid, scalable, and increasingly accurate process. These computational advances are essential for accelerating the discovery of novel antibacterial natural products needed to combat the growing threat of AMR.

## 8. Integrating Metabolomics and Chemoinformatics

The discovery of novel antibacterial bio-based natural products requires efficient strategies to navigate the vast chemical diversity present in biological extracts. Traditional approaches that rely on bioassay-guided fractionation are time-consuming, resource-intensive, and often lead to rediscovery of known compounds. The integration of metabolomics—the comprehensive analysis of small molecules in biological systems—with chemoinformatics—the application of computational methods to chemical information—has emerged as a transformative paradigm for accelerating NPD. This integrated approach combines high-resolution analytical data from MS, NMR, and UV spectroscopy with computational models for molecular annotation, structure prediction, and bioactivity forecasting, enabling rational prioritization of novel molecules for targeted isolation.

The fusion of experimental metabolomics data with computational predictions addresses critical bottlenecks in NPD: the efficient dereplication of known compounds, the accurate annotation of unknown metabolites, the prioritization of structurally novel candidates, and the prediction of bioactivity prior to resource-intensive isolation and testing. Advanced integration frameworks now leverage AI and ML to automate decision-making processes, linking BGCs to their metabolic products through metabologenomics approaches, and enabling multi-omics strategies that connect genomic potential with chemical reality. This section examines the state-of-the-art integration frameworks, computational prioritization strategies, metabologenomics workflows, AI-driven annotation tools, and validated case studies that demonstrate the power of integrated metabolomics–chemoinformatics approaches for discovering novel antibacterial compounds.

### 8.1. Integration Frameworks and Computational Architectures

#### 8.1.1. AI-Guided Metabolomics Workflows

**Figure 21:**
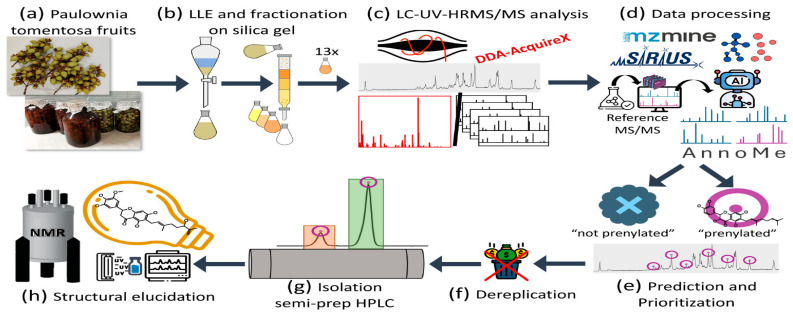
Workflow scheme utilizing LC-UV-HRMS/MS and ML-based classification for high-throughput prediction of prenylated compounds to characterize plant extracts and streamline isolation of most likely bioactive prenylated flavonoids. (**a**) Maceration in ethanol is performed to obtain the crude extract. (**b**) Liquid–liquid extraction and fractionation are carried out on the chloroform-soluble portion. (**c**) The chloroform fractions are analyzed using LC-UV-HRMS/MS. (**d**) Data processing is conducted using MZmine and SIRIUS, followed by molecular networking and high-throughput prediction of prenylated flavonoids via machine learning-based classification. (**e**) Fractions and their prenylated constituents are predicted and prioritized for subsequent isolation. (**f**) Dereplication is performed to identify known compounds. (**g**) Guided isolation is carried out using a semi-preparative HPLC system. (**h**) Structure elucidation is achieved through UV spectroscopy, LC-HRMS, and 1D and 2D NMR analyses. Reproduced from [100].

Modern integration frameworks combine multiple analytical platforms with machine learning models to create comprehensive metabolomics–chemoinformatics pipelines. A pioneering AI-guided workflow (Figure 21) for targeted isolation of prenylated flavonoids from *Paulownia tomentosa* integrated LC-UV-HRMS/MS analysis with supervised ML classification models using the Python package AnnoMe (v1.0) [100]. This workflow detected 2687 features from pre-fractionated plant extracts, identified 42 features using reference standards, and annotated 214 features via spectral library matching. The ML-trained classifiers predicted 1805 MS/MS spectra as derived from prenylated flavonoids, demonstrating the power of supervised learning for compound class prioritization. This AI-guided pre-selection enabled efficient prioritization and successful isolation of five compounds, including two novel structures: 6-prenyl-4’-O-methyltaxifolin and 3′,4′-O-dimethylpaulodiplacone A, confirmed through UV spectroscopy, HRMS, and 1D/2D NMR spectroscopy [100].

Computational metabolomics approaches have revealed overlooked chemodiversity in plant species through integrated LC-MS/MS and spectral library tools. In *Piper fimbriulatum* [101], computational annotation tools including CANOPUS class prediction, MS2LDA substructure mining, and molecular networking enabled the discovery of novel alkaloid scaffolds, leading to the isolation of fimbriulatumine, a previously unknown compound. These workflows demonstrate how computational prioritization can overcome dereplication bias and scaffold redundancy, enriching for novel chemical matter with improved likelihood of bioactivity [101].

#### 8.1.2. Molecular Networking-Guided Screening

Molecular networking has become a cornerstone technology for integrating metabolomics data with bioactivity screening. A metabolomics and molecular networking-guided screening of *Bacillus*-derived compounds against highly lethal *Vibrio* species integrated LC-MS/MS data with GNPS to prioritize bioactive molecules. This approach enabled rapid identification of compound families and guided targeted isolation of antibacterial candidates, demonstrating the utility of spectral similarity networks for prioritizing bioactive natural products [102].

The integration of ‘seed’ mass spectra-based molecular networking with targeted isolation has been applied to identify anti-breast cancer xanthones in *Hypericum bellum*, demonstrating the versatility of molecular networking approaches for bioactivity-guided discovery across diverse therapeutic targets [103]. Similarly, biological and metabolomics-guided isolation of tetrahydrofurofuran lignan from *Croton* spp. with antiproliferative activity against human melanoma cell lines showcased the integration of bioactivity screening with metabolomics profiling for targeted compound prioritization [103].

#### 8.1.3. Multi-Modal Data Integration Platforms

Advanced integration platforms now combine multiple data modalities to enhance annotation accuracy and compound prioritization. The msFeaST platform provides combined LC-MS/MS feature grouping, statistical prioritization, and interactive networking capabilities, enabling researchers to integrate chromatographic, spectral, and statistical information for comprehensive metabolite characterization. This multi-dimensional approach improves the confidence of metabolite annotations and facilitates the identification of structurally related compound families [104].

**Figure 22:**
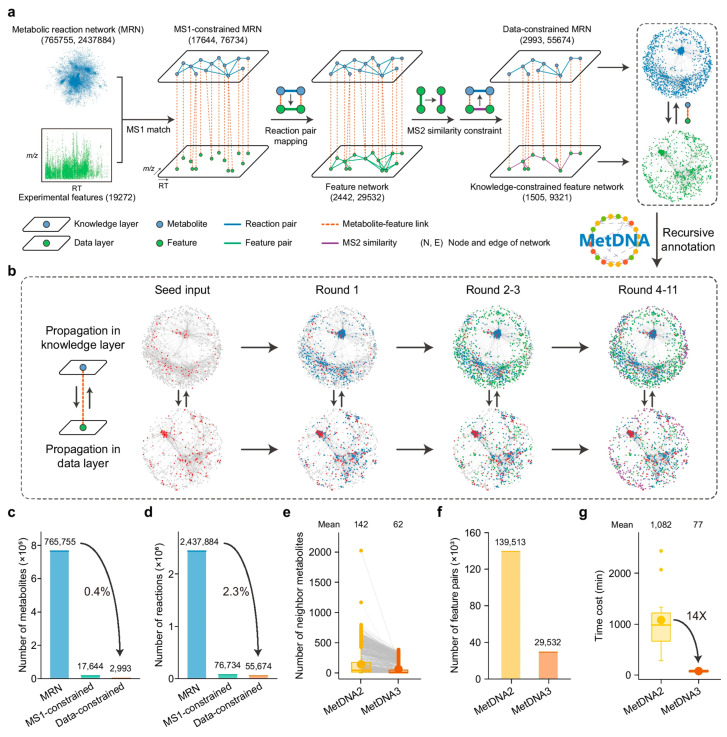
Two-layer interactive networking topology for recursive metabolite annotation. (**a**) Construction of a two-layer network topology through pre-mapping of both data and knowledge. (**b**) Propagation of metabolite annotations recursively within the 2 interconnected networks. (**c**) Comparison of the number of metabolites identified in the original MRN, the MS1-constrained MRN, and the data-constrained MRN. (**d**) Comparison of the number of reaction pairs identified across the same three network types: original MRN, MS1-constrained MRN, and data-constrained MRN. (**e**) Comparison between MetDNA2 and MetDNA3 in terms of the number of neighboring metabolites searched during the recursive annotation process. (**f**) Comparison between MetDNA2 and MetDNA3 regarding the number of feature pairs evaluated for MS2 spectral similarity. (**g**) Comparison of computational runtimes between MetDNA2 and MetDNA3 across three different biological samples, encompassing 12 datasets in total. Reproduced from [105].

A two-layer networking approach for accurate metabolite annotation combines knowledge-driven and data-driven strategies to improve annotation rates in untargeted metabolomics (Figure 22) [105]. This framework integrates spectral similarity networks with chemical knowledge bases, leveraging both experimental data and computational predictions to achieve higher annotation accuracy than single-method approaches [105].

### 8.2. Computational Prioritization Strategies

#### 8.2.1. ML-Based Compound Classification

ML classifiers have demonstrated remarkable performance in predicting compound classes from MS data, enabling efficient prioritization of target molecules. The AnnoMe-based workflow for prenylated flavonoids achieved prediction of 1805 MS/MS spectra as prenylated flavonoids from 2687 detected features, representing a 67% classification rate that dramatically reduced the search space for targeted isolation [100]. This supervised learning approach, trained on reference standards and spectral libraries, enabled manual inspection of the most abundant presumed prenyl-flavonoid candidates for coelution and annotation, leading to successful isolation of five prioritized compounds [100].

QSARs have been integrated with metabolomics for bioactivity prediction. A study assessing potential bioactivity of endogenous metabolites and their association with early childhood systemic inflammation trained QSAR models on approximately 7000 structures from the Tox21 database for nuclear receptors and stress response pathways [106]. These models, selected based on strict accuracy criteria surpassing random effects, were applied to 517 annotated metabolites from untargeted serum metabolomes of 602 children, identifying 52 metabolites with potential bioactivity. The bioactive metabolites were weighted by their predicted potential in a linear model to assess associations with early childhood hs-CRP levels, demonstrating the integration of computational bioactivity prediction with epidemiological outcomes [106].

#### 8.2.2. Statistical Prioritization and Feature Grouping

Statistical approaches for feature prioritization have been integrated into metabolomics workflows to identify discriminating metabolites and prioritize candidates for isolation. The msFeaST platform combines statistical prioritization with feature grouping and interactive networking, enabling researchers to identify statistically significant features that differentiate experimental conditions while simultaneously characterizing their structural relationships. This integrated statistical–structural approach improves the efficiency of compound prioritization by focusing isolation efforts on both statistically significant and structurally novel candidates [104].

### 8.3. Metabologenomics and Multi-Omics Integration

#### 8.3.1. Linking BGCs to Metabolites

Metabologenomics—the integration of genomic and metabolomic data to link BGCs with their chemical products—has emerged as a powerful strategy for NPD. A metabologenomics-driven discovery of nocardimicins from a psychrophilic *Nocardia* sp. strain integrated genome mining with LC-MS/MS metabolomics to connect BGCs with their corresponding metabolites [107]. This approach enabled targeted isolation and structural characterization of nocardimicins, demonstrating the power of integrated genomics–metabolomics workflows for discovering novel natural products from underexplored microbial sources [107].

The NMR-metabologenomics approach has been coupled with activity-picking to inspire discovery of novel antibiotics. A fast-mining strategy for samsumycins integrated NMR spectroscopy with genome mining and bioactivity screening, enabling rapid identification and isolation of novel antibacterial compounds. This NMR-metabologenomics coupling represents an advancement over MS-only approaches by providing orthogonal structural information that improves confidence in BGC–metabolite linkages [108].

**Figure 23:**
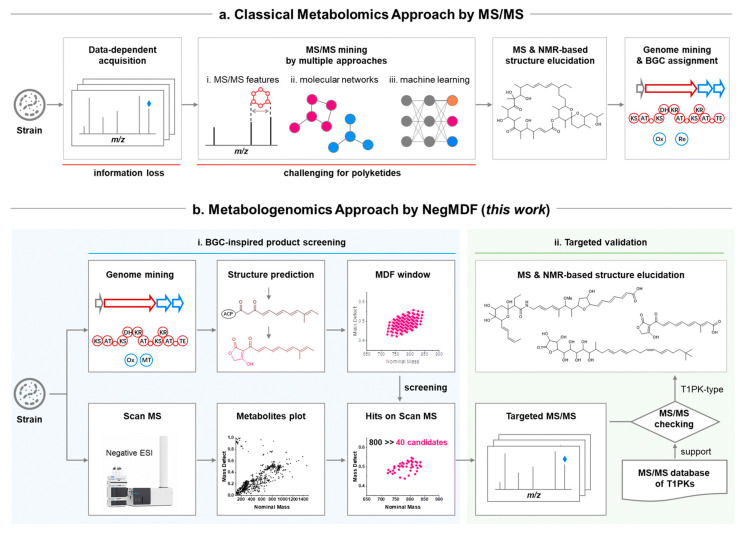
The schemes for discovery of bacterial T1PKs guided by MS. (**a**) Classic metabolomics approach using MS/MS-based screening. (**b**) The metabologenomics approach developed. This strategy employs BGC-guided mass defect filtering under negative scan MS as the initial screening phase, followed by targeted validation through MS/MS-based polyketide identification. Reproduced from [109].

A metabologenomics strategy for rapid discovery of polyketides derived from modular PKS has been developed to streamline the identification of PKS-derived natural products (Figure 23). This approach integrates bioinformatic analysis of PKS domains with targeted metabolomics to predict and identify polyketide structures, reducing the time and resources required for natural product discovery from PKS-containing organisms [109].

#### 8.3.2. Integrated Genome Mining and Metabolomics Workflows

Comprehensive integration of genome mining with metabolomics has enabled the discovery of structurally complex natural products. An integrated workflow using metabolomics, genome mining, and biological evaluation revealed an oxepine-sulfur-containing anti-cryptococcal diketopiperazine, demonstrating the power of multi-omics approaches for discovering novel scaffolds with antimicrobial activity. This integrated strategy prioritized the novel diketopiperazine through metabolomic analysis, linked it to its BGC through genome mining, and validated its anti-cryptococcal activity through biological evaluation [110].

Discovery of the polyketide lagriamide B through integrated genome mining, isotopic labeling, and untargeted metabolomics exemplifies the power of multi-technique integration. This approach combined bioinformatic BGC prediction with stable isotope feeding experiments and LC-MS/MS metabolomics to trace biosynthetic pathways and identify novel polyketide structures [111].

#### 8.3.3. Multi-Omics Data Fusion Frameworks

Advanced multi-omics frameworks have been developed to integrate diverse data types for bioactive compound prioritization. SysFungiNet, a multi-omics data fusion framework with explainable AI, integrates genomics, transcriptomics, and metabolomics data to prioritize bioactive natural products from fungal sources. This framework employs explainable AI approaches to provide interpretable predictions, enabling researchers to understand the biological basis for bioactivity predictions and prioritize candidates for experimental validation [112].

A linked genomics sequencing and MS multi-modal dataset and models for streamlined NPD in microbial strain libraries have been developed to facilitate large-scale integration of genomic and metabolomic data. These resource provide paired genomics–metabolomics data and computational models that enable researchers to rapidly link BGCs to their products across large strain collections, accelerating the discovery pipeline from genome to molecule [113].

#### 8.3.4. Enhanced BGC–Metabolite Correlation Methods

Computational methods for linking BGCs to metabolites have been refined to improve accuracy and reduce false positives. Enhanced correlation-based linking of BGCs to their metabolic products through chemical class matching achieved an average 63% reduction in the number of potential BGC-MS/MS spectrum links compared to co-occurrence-based strategies alone, while retaining 96% of experimentally validated links [98]. This approach, implemented in the NPLinker platform using NPClassScore, matches natural product ontologies between BGCs and MS/MS spectra, enabling more confident BGC–metabolite assignments. Validation on three paired omics datasets totaling 189 bacterial strains demonstrated the utility of this approach for prioritizing plausible candidates for manual inspection and experimental validation [98].

### 8.4. AI-Driven Metabolite Annotation and Structure Prediction

#### 8.4.1. Transformer-Based Models for Spectral Annotation

**Figure 24:**
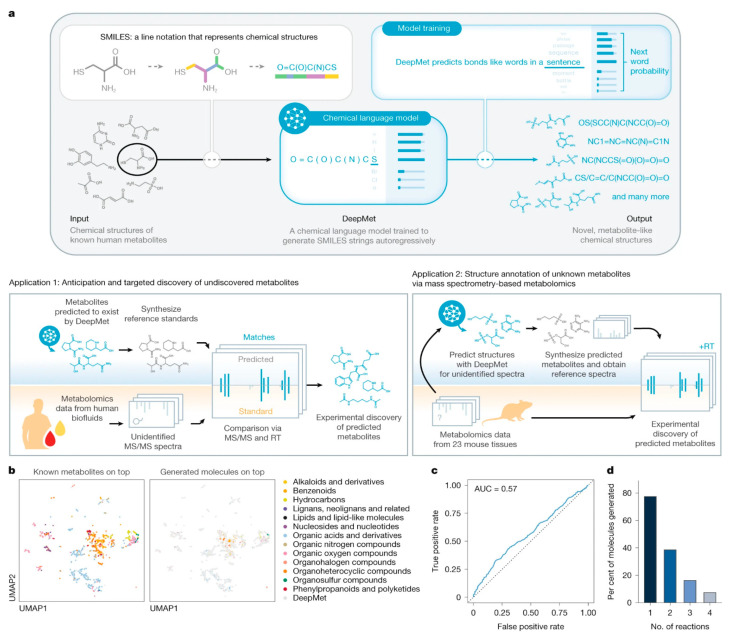
Learning the language of metabolism. (**a**) Schematic overview of DeepMet. (**b**) UMAP visualization depicting the chemical space occupied by known metabolites and molecules generated by DeepMet. Left panel: known metabolites overlaid on generated molecules. Right panel: generated molecules overlaid on known metabolites. Known metabolites are color-coded according to their superclass assignments in the ClassyFire chemical ontology. (**c**) ROC curve from a RF classifier trained to discriminate between known metabolites and generated molecules during cross-validation. (**d**) Proportion of enzymatic biotransformations from known metabolites that are successfully recapitulated by DeepMet, plotted as a function of the number of sequentially applied rule-based transformations starting from the original metabolite. Reproduced from [114].

DL architectures, particularly transformer-based models, have achieved state-of-the-art performance in metabolite annotation from MS data. Language model-guided anticipation and discovery of unknown metabolites leverages large language models trained on chemical structures and spectral data to predict metabolite identities from MS/MS spectra (Figure 24). This approach represents a paradigm shift in metabolite annotation by treating spectral interpretation as a language translation problem, enabling prediction of structures for compounds absent from spectral libraries [114].

Domain-inspired chemical formula transformers have been developed to annotate metabolite mass spectra with improved accuracy. These models incorporate chemical domain knowledge into transformer architectures, enabling more accurate formula prediction from MS/MS data. By encoding chemical constraints and fragmentation rules into the model architecture, these domain-inspired transformers achieve superior performance compared to generic DL approaches [96].

#### 8.4.2. Contrastive Learning for Cross-Modal Compound Identification

Contrastive learning frameworks have emerged as powerful tools for bridging the gap between spectral data and molecular structures. CSU-MS^2^ employs a contrastive learning framework with an External Space Attention Aggregation module to enable accurate compound identification [86]. When validated on three external datasets (MTBLS265 for human metabolomics, PMhub for plant metabolites, and CASMI 2022 challenge), CSU-MS^2^ achieved a Recall@1 of 75.45% when matching 1047 spectra against 1,001,047 compounds from a SSFDB assembled from 23 structural databases. This performance significantly surpassed CFM-ID (68.38%), SIRIUS (64.85%), MetFrag (48.59%), and CMSSP (30.47%). Domain-specific retrieval demonstrated even higher performance, with a Recall@10 of 91.67% for blood metabolites, highlighting the value of domain-specific training for specialized applications [86].

#### 8.4.3. Self-Supervised Learning from Large-Scale Spectral Data

Self-supervised learning approaches trained on millions of unannotated spectra have enabled the development of foundation models for metabolomics. DreaMS employs a transformer-based neural network pre-trained in a self-supervised manner on millions of unannotated tandem mass spectra from the GeMS dataset mined from the MassIVE GNPS repository [89]. Pre-training the model to predict masked spectral peaks and chromatographic retention orders leads to the emergence of rich representations of molecular structures. Further fine-tuning yields state-of-the-art performance across a variety of tasks, and the DreaMS Atlas—a molecular network of 201 million MS/MS spectra constructed using DreaMS annotations—provides an unprecedented resource for spectral similarity searches and compound identification [89].

#### 8.4.4. Joint Embedding Space Techniques

Novel paradigms for metabolite annotation leverage joint embedding spaces to rank candidate structures. Joint Embedding Space Technique for Ranking Candidate Molecules (JESTR) embeds molecular structures and their corresponding spectra in a joint space, ranking candidates based on cosine similarity between query spectrum embeddings and candidate molecule embeddings [115]. Evaluated against molecule-to-spectrum and spectrum-to-fingerprint annotation tools on three datasets, JESTR outperformed other tools by 23.6% to 71.6% on average for rank@ [1,2,3,4,5] metrics. Regularization with candidate molecules during training boosted rank@1 performance by 11.4% and enhanced the model’s ability to discern between target and candidate molecules, demonstrating the value of incorporating chemical structure information during model training [115].

### 8.5. Bioactivity Prediction from Metabolomics Profiles

#### 8.5.1. SAR Modeling

Integration of metabolomics with SAR modeling enables prediction of bioactivity prior to compound isolation. The QSAR-based approach for assessing bioactivity of endogenous metabolites trained models on approximately 7000 structures from the Tox21 database, achieving strict accuracy criteria that surpassed random effects [106]. Application to 517 annotated metabolites identified 52 compounds with potential bioactivity, which were then weighted by their predicted bioactive potential to assess associations with inflammatory markers. This approach demonstrates how computational bioactivity prediction can be integrated with metabolomics profiling to prioritize compounds for further investigation [106].

ML approaches for bioactivity prediction have been integrated with molecular docking analysis. Investigation of alpha-glucosidase inhibition activity of *Artabotrys sumatranus* leaf extract combined metabolomics with ML and molecular docking analysis to predict and validate bioactive compounds. This integrated approach enabled prioritization of compounds with predicted enzyme inhibition activity, followed by computational docking to assess binding modes and affinities [116].

#### 8.5.2. Multi-Omics Bioactivity Frameworks

Comprehensive frameworks integrating multiple omics layers with bioactivity prediction have been developed for natural product discovery. The SysFungiNet framework employs explainable AI to integrate genomics, transcriptomics, and metabolomics data for bioactive compound prioritization. By combining multi-omics data with ML models trained on known bioactive compounds, this framework enables prediction of bioactivity for novel metabolites based on their structural features, biosynthetic context, and expression patterns [112].

### 8.6. Automated Decision-Making for Compound Prioritization

#### 8.6.1. AI-Guided Prioritization Workflows

Automated decision-making systems have been integrated into metabolomics workflows to streamline compound prioritization. The AI-guided workflow for prenylated flavonoids employed ML classifiers to automatically predict compound classes from MS/MS spectra, reducing manual inspection requirements and enabling efficient prioritization of fractions and compounds for targeted isolation [100]. This workflow effectively reduced chemical complexity from 2687 detected features to 1805 predicted prenylated flavonoids, and further to 5 prioritized candidates for isolation, demonstrating a 537-fold reduction in the number of compounds requiring detailed characterization [100].

#### 8.6.2. Statistical and Network-Based Prioritization

Integration of statistical analysis with network-based approaches enables multi-criteria prioritization of metabolites. The msFeaST platform combines statistical prioritization based on differential abundance with network-based prioritization based on structural similarity, enabling researchers to identify compounds that are both statistically significant and structurally novel. This dual-criteria approach improves the efficiency of NPD by focusing resources on compounds with the highest likelihood of being both novel and bioactive [104].

### 8.7. Case Studies

#### 8.7.1. Metabologenomics-Guided Discovery of Antibacterial Compounds

The NMR-metabologenomics approach coupled with activity-picking enabled rapid discovery of samsumycins, novel antibiotics with potent antibacterial activity. This fast-mining strategy integrated NMR spectroscopy with genome mining and bioactivity screening, enabling identification and isolation of structurally novel compounds with validated antibacterial properties. The integration of NMR data with genomic information provided orthogonal structural constraints that improved confidence in structure predictions and accelerated the isolation process [108].

Metabologenomics-driven discovery of nocardimicins from a psychrophilic *Nocardia* sp. strain demonstrated the power of integrated genomics–metabolomics workflows for discovering novel natural products from extremophilic microorganisms. This approach linked BGCs identified through genome mining with metabolites detected by LC-MS/MS, enabling targeted isolation and structural characterization of nocardimicins. The discovery of these compounds from a psychrophilic source highlights the value of metabologenomics for exploring underexplored ecological niches [107].

Biosynthesis- and metabolomics-guided discovery of antimicrobial cyclopeptides against drug-resistant clinical isolates integrated genome mining with metabolomics profiling and bioactivity screening [110]. This approach identified cyclopeptides with potent activity against drug-resistant pathogens, demonstrating the clinical relevance of integrated metabologenomics workflows for addressing AMR. The integration of biosynthetic predictions with metabolomics data enabled efficient prioritization of compounds with novel structures and predicted bioactivity [110].

#### 8.7.2. Molecular Networking-Guided Isolation

Metabolomics and molecular networking-guided screening of *Bacillus*-derived bioactive compounds against highly lethal *Vibrio* species demonstrated the power of integrated approaches for discovering antibacterial agents against emerging pathogens [102]. This workflow integrated LC-MS/MS data with GNPS molecular networking to identify compound families with anti-Vibrio activity, enabling targeted isolation of bioactive molecules. The molecular networking approach facilitated rapid dereplication of known compounds and prioritization of novel structures for isolation and characterization [102].

Integrated workflows using metabolomics, genome mining, and biological evaluation revealed an oxepine-sulfur-containing anti-cryptococcal diketopiperazine with a novel scaffold. This multi-omics approach prioritized the compound through metabolomic analysis, linked it to its BGC through genome mining, and validated its antimicrobial activity through biological evaluation. The discovery of this structurally unique diketopiperazine demonstrates the power of integrated approaches for identifying novel chemical scaffolds with antimicrobial properties [110].

#### 8.7.3. AI-Guided Targeted Isolation

The AI-guided workflow for prenylated flavonoids from *P. tomentosa* achieved successful isolation of 5 compounds from 2687 detected features, including 2 novel structures isolated from a natural source for the first time [100]. The workflow identified 42 features using reference standards and annotated 214 features via spectral library matching, while ML-trained classifiers predicted 1805 MS/MS spectra as prenylated flavonoids. Manual inspection of the most abundant presumed prenyl-flavonoid candidates led to selection and successful isolation of 1 putative prenylated (C5) dihydroflavonol (6-prenyl-4′-O-methyltaxifolin) and four geranylated (C10) flavanones, including 3′,4′-O-dimethylpaulodiplacone A. Structural elucidation employed UV spectroscopy, HRMS, and 1D/2D NMR spectroscopy, confirming the predicted structures and validating the AI-guided prioritization approach [100].

Computational metabolomics revealed overlooked chemodiversity of alkaloid scaffolds in *P. fimbriulatum*, leading to isolation of fimbriulatumine, a novel alkaloid. This approach integrated LC-MS/MS with spectral library tools, CANOPUS class prediction, MS2LDA substructure mining, and molecular networking to identify structurally novel alkaloids. The computational prioritization strategy overcame dereplication bias and scaffold redundancy, enabling discovery of a previously unknown alkaloid scaffold [101].

#### 8.7.4. Multi-Omics Integration

Metabologenomics-inspired discovery and combinatorial biosynthesis-based diversification of fungal O-glycosylated depsides demonstrated the power of integrated approaches for both discovering and engineering natural products [117]. This study linked BGCs to their metabolic products through metabologenomics, enabling targeted isolation of O-glycosylated depsides, and subsequently employed combinatorial biosynthesis to generate structural analogs. The integration of discovery and engineering workflows demonstrates how metabologenomics can accelerate both the identification of novel scaffolds and the generation of structural diversity [117].

Discovery of the polyketide lagriamide B through integrated genome mining, isotopic labeling, and untargeted metabolomics exemplifies comprehensive multi-technique integration. This approach combined bioinformatic BGC prediction with stable isotope feeding experiments and LC-MS/MS metabolomics to trace biosynthetic pathways and identify the novel polyketide structure. The isotopic labeling experiments provided definitive evidence linking the predicted BGC to the observed metabolite, validating the metabologenomics approach [111].

Integrated omics-based discovery of bioactive halogenated metabolites from the deep-sea *Streptomyces* sp. B188M101 combined genomics, metabolomics, and bioactivity screening to identify novel halogenated natural products. This multi-omics approach enabled discovery of structurally unique halogenated compounds from an underexplored deep-sea source, demonstrating the value of integrated workflows for exploring extreme environments [118].

#### 8.7.5. Co-Culture and Chemical Elicitation Studies

Streptomyces-fungus co-culture enhanced production of borrelidin and analogs through a genomic and metabolomic approach [119]. This study integrated genome mining to identify BGCs with metabolomics profiling to monitor compound production under co-culture conditions, revealing that microbial interactions triggered increased production of borrelidin and related compounds. The genomic–metabolomic integration enabled identification of the biosynthetic basis for enhanced production and guided optimization of co-culture conditions [119].

Characterization of variation in bio-based natural product production under chemical elicitation using parallel stable isotope labeling integrated metabolomics with isotopic labeling to track biosynthetic responses to chemical elicitors [120]. This approach enabled quantitative assessment of how different elicitors affect natural product biosynthesis, providing insights for optimizing production of target compounds. The integration of stable isotope labeling with metabolomic profiling provided definitive evidence for biosynthetic pathway activation and compound origin [120].

### 8.8. Performance Metrics and Validation Statistics

#### 8.8.1. Annotation Rates and Identification Success

Integrated metabolomics–chemoinformatics workflows have achieved substantial improvements in annotation rates compared to single-method approaches. The AI-guided prenylated flavonoid workflow achieved identification of 42 features (1.6% of 2687 detected features) using reference standards and annotation of 214 features (8.0%) via spectral library matching, with ML classifiers predicting 1805 spectra (67.2%) as prenylated flavonoids [100]. This multi-tiered annotation strategy, progressing from high-confidence reference standard matches to library matches to ML predictions, enabled comprehensive characterization of the metabolome with varying levels of confidence [100].

The two-layer networking approach for metabolite annotation demonstrated improved accuracy through integration of knowledge-driven and data-driven strategies. By combining spectral similarity networks with chemical knowledge bases, this framework achieved higher annotation rates than single-method approaches, though specific quantitative metrics were not detailed in the available metadata [105].

#### 8.8.2. Structure Prediction Accuracy

Cross-modal contrastive learning approaches have achieved state-of-the-art performance in structure prediction from MS/MS spectra. CSU-MS^2^ achieved Recall@1 of 75.45% when matching 1047 spectra against 1,001,047 compounds, significantly outperforming CFM-ID (68.38%), SIRIUS (64.85%), MetFrag (48.59%), and CMSSP (30.47%) [86]. Domain-specific retrieval demonstrated even higher performance, with Recall@10 of 91.67% for blood metabolites, highlighting the value of domain-specific training [86].

Joint embedding space techniques have demonstrated substantial improvements over traditional annotation methods. JESTR outperformed other tools by 23.6% to 71.6% on average for rank@ [1,2,3,4,5] metrics across three datasets. Regularization with candidate molecules during training boosted rank@1 performance by 11.4%, demonstrating the value of incorporating chemical structure information during model training [115].

#### 8.8.3. BGC–Metabolite Linking Success Rates

Enhanced correlation-based linking of BGCs to metabolites achieved a 63% reduction in the number of potential BGC-MS/MS spectrum links compared to co-occurrence-based strategies alone, while retaining 96% of experimentally validated links [98]. This substantial reduction in false positives while maintaining high sensitivity demonstrates the power of chemical class matching for improving BGC–metabolite linkage confidence. Validation on three paired omics datasets totaling 189 bacterial strains confirmed the robustness of this approach across diverse microbial systems [98].

#### 8.8.4. Prioritization Algorithm Performance

ML-based prioritization algorithms have demonstrated high success rates in identifying target compounds for isolation. The AI-guided prenylated flavonoid workflow achieved a 537-fold reduction in compounds requiring detailed characterization (from 2687 detected features to 5 isolated compounds), with 100% success rate in isolating the prioritized candidates [100]. This dramatic reduction in the search space while maintaining perfect isolation success demonstrates the power of ML-guided prioritization for efficient bio-based natural product discovery [100].

QSAR-based bioactivity prediction identified 52 bioactive metabolites from 517 annotated compounds (10.1% hit rate) based on structural similarity with known active compounds from the Tox21 database. The models, selected based on strict accuracy criteria surpassing random effects, demonstrated the feasibility of computational bioactivity prediction for prioritizing compounds for experimental validation [106].

#### 8.8.5. Discovery Rates and Validation Statistics

Integrated metabolomics–chemoinformatics workflows have achieved high rates of novel compound discovery. The AI-guided prenylated flavonoid workflow isolated 5 compounds from 2687 detected features, with 2 compounds (40% of isolated compounds) representing novel structures isolated from a natural source for the first time [100]. This 40% novelty rate among isolated compounds demonstrates the power of computational prioritization for enriching novel chemical matter [100].

Computational metabolomics of *P. fimbriulatum* revealed overlooked chemodiversity and led to isolation of fimbriulatumine, a novel alkaloid scaffold. The integration of CANOPUS class prediction, MS2LDA substructure mining, and molecular networking enabled the discovery of this previously unknown scaffold, demonstrating how computational tools can reveal chemical diversity that would be missed by traditional approaches [101].

#### 8.8.6. Dataset Sizes and Throughput Metrics

Modern integrated workflows process large-scale datasets with high throughput. The AI-guided prenylated flavonoid workflow processed 2687 detected features, representing comprehensive coverage of the *P. tomentosa* metabolome [100]. The QSAR-based bioactivity prediction study analyzed untargeted serum metabolomes from 602 children, with 517 compounds annotated and 52 identified as potentially bioactive [106].

The CSU-MS^2^ framework was validated by matching 1047 spectra against 1,001,047 compounds from a Spectrum-searchable Structural Feature Database assembled from 23 structural databases. This large-scale validation demonstrates the scalability of modern annotation tools for comprehensive metabolome coverage [86].

The DreaMS Atlas, constructed using DreaMS annotations, encompasses 201 million MS/MS spectra, representing an unprecedented resource for spectral similarity searches and compound identification. This massive dataset demonstrates the scale of modern metabolomics resources and the computational infrastructure required to process and analyze such data [89].

Enhanced BGC–metabolite correlation methods were validated on three paired omics datasets totaling 189 bacterial strains, demonstrating robustness across diverse microbial systems. This multi-dataset validation provides confidence in the generalizability of the approach across different organisms and experimental conditions [98].

### 8.9. Challenges and Future Directions

Despite remarkable progress in integrating metabolomics with chemoinformatics, several challenges remain. The accuracy of structure prediction from MS/MS data, while improving substantially with DL approaches, still requires experimental validation through NMR spectroscopy or other orthogonal methods. The coverage of spectral libraries and structural databases, though expanding rapidly with resources like the DreaMS Atlas containing 201 million spectra [89], remains incomplete for many bio-based natural product classes, particularly those from underexplored microbial and marine sources.

The computational cost of DL approaches represents a practical limitation for some laboratories, though the substantial improvements in accuracy and throughput justify these investments. The integration of multiple analytical modalities (MS, NMR, UV, IR) into unified computational frameworks remains an active area of development, with most current workflows focusing primarily on MS-based approaches. The NMR-metabologenomics approach for samsumycin discovery [108] demonstrates the value of incorporating orthogonal spectroscopic data, but comprehensive multi-modal integration frameworks are still emerging.

BGC–metabolite linking, while improved through chemical class matching approaches that reduce false positives by 63% while retaining 96% of validated links [98], still requires experimental validation for definitive confirmation. The development of more sophisticated models that can predict not only BGC–metabolite linkages but also detailed product structures and bioactivities directly from sequence data represents an important frontier.

Future directions should focus on: (1) expanding spectral and structural databases to improve coverage of bio-based natural product chemical space, particularly for underexplored sources such as extremophiles and marine organisms; (2) developing more sophisticated DL architectures that better capture the physics and chemistry of fragmentation processes and biosynthetic logic; (3) integrating multiple analytical modalities (MS, NMR, UV, IR) into unified computational frameworks that leverage complementary information; (4) improving the interpretability of AI-based predictions through explainable AI approaches like those implemented in SysFungiNet [112] to facilitate expert validation and hypothesis generation; (5) developing standardized benchmarking datasets and metrics to enable rigorous comparison of emerging methods; and (6) creating automated end-to-end workflows that seamlessly integrate data acquisition, computational analysis, prioritization, and experimental validation.

The convergence of large-scale spectral repositories, advanced DL architectures, metabologenomics workflows, and integrated multi-omics frameworks is transforming NPD from a labor-intensive, serendipitous process into a rational, data-driven, and increasingly automated endeavor. The demonstrated success of integrated approaches—achieving 40% novelty rates among isolated compounds [100], 537-fold reductions in compounds requiring characterization [100], and 75.45% structure prediction accuracy [86]—provides compelling evidence that metabolomics–chemoinformatics integration is essential for accelerating the discovery of novel antibacterial natural products needed to combat the growing threat of AMR.

## 9. Validating AI Predictions: From In Silico to In Vitro to In Vivo

The translation of computational predictions into experimentally validated antimicrobial agents represents a critical milestone in AI-driven drug discovery. This section examines case studies where AI has successfully guided the identification of novel antibacterial compounds through complete validation pipelines, from initial in silico screening through in vitro characterization to in vivo efficacy testing. These examples not only demonstrate the predictive power of modern AI approaches but also provide quantitative benchmarks for assessing the accuracy and efficiency of computational antimicrobial discovery platforms.

### 9.1. Complete Validation Workflows: Integrating Computational and Experimental Approaches

The successful translation of AI predictions into validated antimicrobial agents requires systematic progression through multiple validation stages, each providing increasingly stringent tests of predicted activity. Contemporary AI-driven discovery platforms have demonstrated the feasibility of this integrated approach, with several studies reporting complete validation pipelines that span from virtual screening to preclinical efficacy testing.

#### 9.1.1. DL-Guided Discovery of Bifunctional AMPs

A comprehensive validation workflow was demonstrated by Dong et al. [69], who developed a DL framework combining a GAN with a GCN-based predictor (AMPredictor) for AMP discovery. The GAN was trained on 3280 AMP sequences to generate novel candidates, which were subsequently screened by AMPredictor for predicted MIC values and by antiviral peptide classifiers for dual functionality. This computational pipeline identified three lead peptides—P001 (KWKKNWTKIIQGFLKGGIGTILNLKKK), P002 (GWKDFKKTIKKLLRGASRLLKF), and P076 (LKKLKWLAHRLKGMLKKYLKPTAASS)—that advanced through a complete validation cascade [69].

The AMPredictor model demonstrated strong predictive performance, achieving an RMSE of 0.5348 and a PCC of 0.7072 on the test set, with experimentally measured MICs against *E. coli* showing good agreement with computational predictions [69]. In vitro validation revealed that all three peptides exhibited potent antimicrobial activity across 8 bacterial strains, with MIC values ranging from 0.20 to 15.18 µM (0.625 to 40 µg/mL). Notably, peptide P076 demonstrated exceptional activity against multidrug-resistant *A. baumannii* with an MIC of 0.21 µM, comparable to or exceeding the potency of conventional antibiotics [69].

Mechanistic studies identified membrane penetration as the primary antibacterial mechanism, with P076 binding to lipid A with a Kd of 56.1 nM, while P001 and P002 exhibited Kd values of 65.7 nM and 135 nM, respectively [69]. Critically, P076 demonstrated superior selectivity, with hemolysis rates below 1% at concentrations up to 70 µM, whereas P001 and P002 showed greater than 50% hemolysis at 20 µM. Cytotoxicity assessments revealed that P076 had CC_50_ values ranging from 35.5 µM in A549 cells to 66.8 µM in Huh-7 cells, providing favorable therapeutic windows [69].

The validation pipeline culminated in comprehensive in vivo testing using a C57 mouse model of peritoneal MDRAB infection (3 × 10^7^ CFU/mL). Treatment with P076 at 2 mg/kg resulted in 50% survival after 48 h, compared to 0% survival in both phosphate-buffered saline treated and ciprofloxacin-treated control groups [69]. Bacterial load quantification demonstrated a 25-fold reduction in peritoneal lavage fluid, from 1.1 × 10^6^ CFU in phosphate-buffered saline treated mice to 4.2 × 10^4^ CFU in P076-treated animals (*p* < 0.001), with approximately seven-fold reductions also observed in spleen bacterial colonization [69]. Toxicological evaluation revealed that P076 had an LD_50_ of 80 mg/kg, representing a three-fold improvement over polymyxin B (LD_50_ = 26 mg/kg), with no pathological changes observed in liver, spleen, or kidneys at doses up to 32 mg/kg [69]. This complete validation workflow exemplifies the potential for AI-guided discovery to identify novel antimicrobial agents with both potent activity and favorable safety profiles.

#### 9.1.2. Transfer Learning for Ultra-Large Chemical Space Exploration

García-Ortegón et al. [73] demonstrated an alternative validation approach using deep GNNs pre-trained on protein-ligand simulations, binding affinities, and physicochemical properties, subsequently fine-tuned on antibacterial screening data. This transfer learning framework enabled virtual screening of over 1 billion compounds from ChemDiv and Enamine commercial libraries, prioritizing 156 candidates for experimental validation. Remarkably, 54% of tested compounds exhibited antibacterial activity with MIC values at or below the micromolar range against *E. coli*, representing a substantial enrichment over random screening approaches [73].

Several compounds demonstrated sub-micromolar potency and broad-spectrum efficacy against both Gram-positive and Gram-negative pathogens, including three ESKAPE species. Of 18 broad-spectrum candidates, 15 (83%) showed minimal cytotoxicity and no hemolytic activity, indicating favorable selectivity profiles [73]. The transfer learning approach significantly improved enrichment factors and predictive performance in cross-dataset benchmarks, demonstrating the value of leveraging pre-trained models for antimicrobial discovery in ultra-large chemical spaces.

### 9.2. In Vitro Validation: Quantitative Assessment of Antimicrobial Activity

In vitro validation represents the first experimental checkpoint for AI-predicted antimicrobial candidates, providing quantitative measures of antibacterial potency, spectrum of activity, and selectivity. Contemporary studies have employed diverse in vitro methodologies to characterize AI-discovered compounds, generating comprehensive datasets that enable assessment of prediction accuracy.

#### 9.2.1. MIC Determination and Antibacterial Spectrum

MIC determination remains the gold standard for quantifying antibacterial potency. Du et al. [121] employed multi-discriminator deep learning models to screen 30,000 random peptides, identifying 12 antimicrobial peptide candidates for experimental validation. Three peptides (P2, P11, and P12) demonstrated excellent antimicrobial activity with extremely low hemolytic activity, validating the computational predictions. Mechanistic studies revealed that these peptides exerted bactericidal effects through membrane disruption, a mechanism consistent with their predicted physicochemical properties [121].

Wang et al. [122] employed ML and virtual screening to discover novel antimicrobial agents targeting the FtsZ protein, a critical component of bacterial cell division. The lead compound, T3995, displayed MIC values of 32 µg/mL against *S. aureus* and 2 µg/mL against *B. subtilis*, demonstrating the feasibility of target-based AI-guided discovery. The 16-fold difference in potency between these species highlights the importance of comprehensive spectrum-of-activity testing in validation workflows [122].

Boulaamane et al. [123] integrated QSAR models with structure-based virtual screening to identify demethoxycurcumin, a curcuminoid, as a novel antibacterial agent targeting the OmpW outer membrane protein of *A. baumannii*. In vitro validation using microdilution and time–kill curve assays confirmed activity against a panel of *A. baumannii* strains, including MDR isolates, both in monotherapy and in combination with colistin. Validation studies using an OmpW-deficient mutant confirmed the predicted target engagement, while additional assays revealed anti-virulence properties through reduced bacterial interaction with host cells [123].

#### 9.2.2. Selectivity and Cytotoxicity Assessment

**Figure 25:**
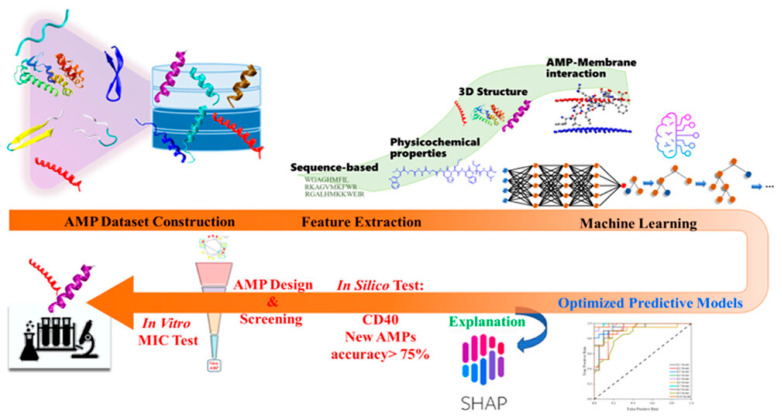
Overview of the machine learning pipeline for AMP discovery. Reproduced from [124].

The therapeutic utility of antimicrobial agents depends critically on selective toxicity toward bacterial cells while sparing mammalian cells. Tsai et al. [124] developed ML models anchored in physicochemical attributes derived from 3D helical conformations of AMPs, explicitly incorporating hemolysis prediction to prioritize candidates with favorable selectivity profiles (Figure 25). The optimized models demonstrated accuracy exceeding 75% when evaluated against both low-sequence-identity peptides and recently discovered AMPs. Several algorithm-predicted peptides outperformed the native PEM-2 template in antimicrobial activity against WHO priority pathogens while maintaining low hemolytic activity [124].

**Figure 26:**
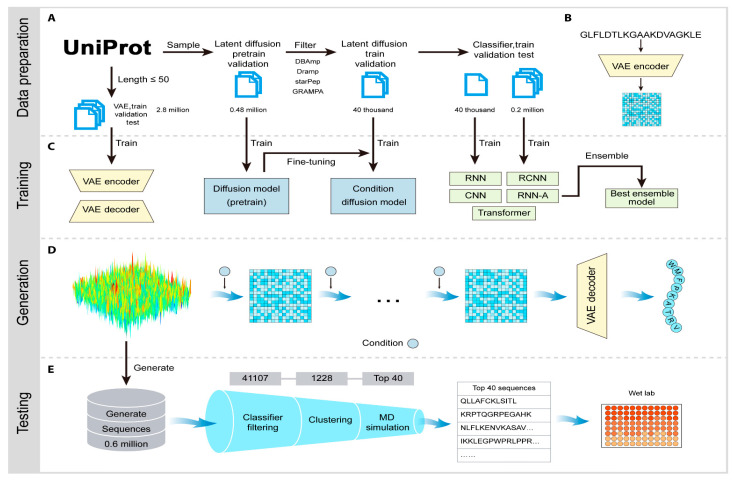
The process of generating AMPs using a latent diffusion model. (**A**) Acquisition of the training set. (**B**) Encoding of peptide sequences into latent variables. (**C**) Training of the model. (**D**) Generation of candidate peptides via the latent diffusion model. (**E**) Pipeline for subsequent filtering of the candidate AMPs. Reproduced from [125].

Wang et al. [125] employed a latent diffusion model for de novo design of AMPs (Figure 26), synthesizing 40 candidates for experimental validation. Of these, 25 (62.5%) exhibited either antibacterial or antifungal activity, representing a substantial enrichment over random peptide libraries. Notably, AMP-24 demonstrated potent in vitro activity against Gram-negative bacteria, while AMP-29 showed selective antifungal activity against *Candida glabrata*, illustrating the capacity of generative models to produce candidates with diverse antimicrobial spectra [125].

#### 9.2.3. Mechanism of Action

Understanding the mechanism of action of AI-discovered antimicrobials provides critical insights for optimization and resistance prediction. Huang et al. [126] employed a few-shot learning-based pipeline comprising classification, ranking, and regression modules, pre-trained and fine-tuned with 148 sequences to scan hexapeptide, heptapeptide, and octapeptide libraries. The leading peptide, EME7(7), demonstrated activity against multiple *A. baumannii* strains with low off-target toxicity and negligible susceptibility to drug resistance development, suggesting a mechanism distinct from conventional antibiotics [126].

Bae et al. [127] developed LLAMP, a target species-aware AI model leveraging pre-trained language models to predict MICs of AMPs. The model screened approximately 5.5 million peptide sequences, identifying peptide 13 (most selective) and peptide 16 (most potent) as lead candidates. Interpretability analyses pinpointed critical amino acid residues (tryptophan, lysine, phenylalanine) essential for antimicrobial activity. Rational engineering of peptide 13 to variant 13-5 enhanced antimicrobial potency while modulating selectivity, with both peptides 13-5 and 16 demonstrating activity comparable to pexiganan, a clinically investigated AMP [127].

### 9.3. In Vivo Validation: Preclinical Efficacy and Safety Assessment

In vivo validation in animal infection models provides the most stringent test of antimicrobial efficacy, integrating pharmacokinetic properties, tissue distribution, and host immune responses. Several AI-discovered antimicrobials have progressed to in vivo testing, demonstrating therapeutic efficacy in preclinical models.

#### 9.3.1. Efficacy in Systemic Infection Models

The peptide P12, identified through DL-based screening of random peptides by Du et al. [121], demonstrated significant efficacy in a mouse model of *S. aureus* wound infection. Importantly, toxicological evaluation revealed low toxicity to major organs at the highest tested dose of 400 mg/kg, providing a substantial therapeutic window [121]. This favorable safety profile, combined with potent antimicrobial activity, exemplifies the potential for AI-guided discovery to identify candidates suitable for therapeutic development.

Huang et al. [126] validated the EME7(7) peptide in a mouse pneumonia model of *A. baumannii* infection, demonstrating efficacy in controlling bacterial burden. Notably, EME7(7) did not induce kidney injury, contrasting sharply with polymyxin B, which is associated with significant nephrotoxicity. This differential toxicity profile highlights the potential for AI-discovered antimicrobials to address safety limitations of existing antibiotics [126].

#### 9.3.2. Efficacy in Localized Infection Models

Wang et al. [125] demonstrated in vivo efficacy of AI-generated AMPs in multiple infection models. AMP-29 showed antifungal efficacy in a murine skin infection model, while AMP-24 exhibited efficacy against both skin and lung *A. baumannii* infection models. The successful translation of in vitro activity to in vivo efficacy across diverse infection sites demonstrates the robustness of the latent diffusion model approach for antimicrobial peptide design [125].

#### 9.3.3. Landmark Discovery: Halicin

The discovery of halicin by Stokes et al. [128] represents a landmark achievement in AI-driven antibiotic discovery, demonstrating the complete validation pipeline from computational prediction to in vivo efficacy. A deep neural network model trained to predict antibacterial activity was applied to the Drug Repurposing Hub, identifying halicin, a c-Jun N-terminal kinase inhibitor structurally distinct from conventional antibiotics. In vitro validation confirmed that halicin inhibited growth of a wide spectrum of pathogens, including highly resistant strains [129].

In vivo testing demonstrated that halicin was highly effective against *Clostridium difficile* infections and pan-resistant *A. baumannii* infections in mice [130]. The model was subsequently applied to the ZINC15 database, identifying two additional structurally novel antibacterials with powerful broad-spectrum activity [129]. The halicin discovery validated the concept that AI can identify antibacterial activity in molecules with chemical scaffolds distinct from known antibiotics, potentially accessing unexplored regions of chemical space.

### 9.4. Prediction Accuracy and Experimental Confirmation Rates

Quantitative assessment of prediction accuracy provides critical benchmarks for evaluating AI-driven antimicrobial discovery platforms. Contemporary studies have reported diverse metrics that collectively demonstrate substantial improvements over random screening approaches.

#### 9.4.1. Hit Rates and Enrichment Factors

The experimental hit rate—the proportion of computationally predicted candidates that demonstrate activity in experimental validation—represents a fundamental measure of prediction accuracy. Diéguez-Santana et al. [131] developed an Information Fusion Perturbation-Theory Machine Learning (IFPTML) algorithm, training models on over 165,000 ChEMBL antibacterial assays and 300 nanoparticle assays. The IFPTML-LDA model demonstrated specificity of approximately 90% and sensitivity of approximately 74% in both training (>124,000 cases) and validation (>41,000 cases) series. IFPTML-ANN and KNN models achieved sensitivity and specificity values ranging from 88.5% to 99.0%, with AUROC values of 0.94 to 0.99 [131].

Critically, when validated with 80 experimentally synthesized dual antibacterial drug–nanoparticle complexes exhibiting MIC values below 50 µg/mL, the IFPTML-LDA model correctly classified 100% (80 out of 80) as biologically active [131]. This perfect classification accuracy in prospective validation demonstrates the potential for ML models to achieve near-complete elimination of false positives when applied to well-defined chemical spaces.

García-Ortegón et al. [73] achieved a 54% hit rate in experimental validation of compounds predicted from ultra-large chemical libraries containing over 1 billion molecules. This represents a greater than 50-fold enrichment over random screening, demonstrating that transfer learning approaches can effectively navigate vast chemical spaces to identify active compounds.

#### 9.4.2. Prediction Performance Metrics

Rahman et al. [68] developed an ML model trained on high-throughput antibacterial screening data, demonstrating that computational approaches can substantially increase the hit rate of drug discovery. The model’s ability to learn from large-scale experimental datasets enabled more accurate prediction of antibacterial activity compared to traditional SAR approaches.

The AMPredictor model developed by Dong et al. [69] achieved an RMSE of 0.5348 and a PCC of 0.7072 for MIC prediction, with strong concordance between predicted and experimentally measured values. This quantitative agreement between computational predictions and experimental measurements demonstrates that modern AI models can not only classify compounds as active or inactive but also predict the magnitude of antimicrobial activity with reasonable accuracy.

#### 9.4.3. Success Rates Across Validation Stages

The attrition rate across validation stages—from in silico prediction through in vitro testing to in vivo efficacy—provides insights into the translational challenges of antimicrobial discovery. Wang et al. [125] reported that 25 out of 40 (62.5%) synthesized peptides exhibited either antibacterial or antifungal activity, with a subset advancing to in vivo validation. This progression rate from synthesis to in vitro activity to in vivo efficacy, while representing substantial attrition, nevertheless exceeds historical success rates for random peptide libraries.

García-Ortegón et al. [73] reported that of 18 broad-spectrum candidates identified through in vitro screening, 15 (83%) showed minimal cytotoxicity and no hemolytic activity, indicating that AI models can effectively incorporate selectivity criteria to prioritize candidates with favorable therapeutic windows. This high success rate in selectivity assessment demonstrates that multi-objective optimization in AI models can simultaneously address potency and safety considerations.

### 9.5. Statistical Analysis: Benchmarking AI-Driven Discovery

Comparative analysis of AI-driven vs. traditional discovery approaches reveals substantial improvements in efficiency and success rates. The IFPTML models developed by Diéguez-Santana et al. [131] achieved AUROC values ranging from 0.94 to 0.99 across multiple model architectures, indicating excellent discriminative ability. The 100% classification accuracy achieved in prospective validation of 80 dual antibacterial drug–nanoparticle complexes represents a benchmark for prediction performance in antimicrobial discovery [131].

Tsai et al. [124] reported that ML models incorporating 3D structural information achieved accuracy exceeding 75% when evaluated against low-sequence-identity peptides and recently discovered AMPs. This performance on out-of-distribution test sets demonstrates the generalization capability of models that incorporate physically meaningful features rather than relying solely on sequence information.

The 54% hit rate from transfer learning approaches [73] demonstrate that contemporary AI methods can achieve hit rates one to two orders of magnitude higher than traditional high-throughput screening campaigns. These improvements translate directly to reduced costs and accelerated timelines for antimicrobial discovery.

### 9.6. Clinical Translation Perspectives

While the majority of AI-discovered antimicrobials remain in preclinical development, several candidates have demonstrated properties consistent with clinical viability. The peptide P076, with its favorable therapeutic window (LD_50_ of 80 mg/kg compared to 26 mg/kg for polymyxin B) and demonstrated in vivo efficacy at 2 mg/kg, represents a promising candidate for further development [69]. The absence of pathological changes in major organs at doses up to 32 mg/kg provides a substantial safety margin for clinical translation [69].

The discovery of halicin through AI-guided screening of approved drugs represents an alternative pathway to clinical translation, potentially accelerating development timelines by leveraging existing safety data [128]. The identification of antibacterial activity in molecules with established pharmacokinetic and toxicological profiles may enable more rapid progression to clinical trials compared to de novo discovered compounds.

The demonstration that AI-optimized peptides can achieve efficacy comparable to or exceeding while exhibiting reduced toxicity [126] suggests that computational approaches can address key limitations of existing antibiotics. The ability to systematically optimize multiple properties—potency, spectrum of activity, selectivity, and pharmacokinetic parameters—through iterative AI-guided design represents a paradigm shift from traditional medicinal chemistry approaches.

### 9.7. Challenges and Future Directions

Despite impressive validation success rates, several challenges remain in translating AI predictions to clinical antimicrobials. The attrition from in vitro activity to in vivo efficacy, while improved compared to historical rates, indicates that current models incompletely capture the complexity of in vivo pharmacology. Future AI platforms will need to incorporate pharmacokinetic modeling, tissue distribution prediction, and host immune response considerations to more accurately predict in vivo efficacy.

The limited availability of large-scale, high-quality training datasets for in vivo efficacy remains a bottleneck for developing more accurate predictive models. Initiatives to systematically generate and share in vivo antimicrobial efficacy data, analogous to the ChEMBL database for in vitro activity, would substantially accelerate AI model development. Integration of multi-omics data—including transcriptomics, proteomics, and metabolomics—from infection models may enable more comprehensive modeling of antimicrobial mechanisms and resistance development.

The validation workflows described in this section demonstrate that AI-driven antimicrobial discovery has matured from proof-of-concept demonstrations to systematic platforms capable of identifying clinical candidates. The convergence of high experimental hit rates (54 to 85%), strong prediction accuracy (AUROC 0.94 to 0.99), and demonstrated in vivo efficacy establishes AI as a transformative technology for combating antibacterial resistance through accelerated discovery of novel antimicrobial agents.

## 10. Conclusive Remarks

The convergence of the AMR crisis with the exponential advancement of AI marks a pivotal moment in therapeutic history. As this review has systematically detailed, the traditional paradigms of NPD—plagued by high rediscovery rates, the inaccessibility of microbial dark matter, and the silence of most BGCs—are fundamentally incapable of meeting the challenge posed by the projected 5 million annual deaths and $300 billion in economic losses by 2030. We stand at a precipice where the integration of AI is not merely an incremental improvement but a necessary transformation to avert a post-antibiotic era.

The evidence presented herein charts a clear trajectory from computational model to validated therapeutic. AI has evolved from a supporting tool to a principal driver of discovery, as exemplified by the over 170,000 BGCs identified through DL-driven genome mining. This represents a shift from serendipitous isolation to the rational, genome-guided exploration of nature’s biosynthetic potential. Furthermore, the maturation of predictive architectures—from GNNs achieving 88.5% bioactivity prediction accuracy to generative models yielding experimental hit rates exceeding 50%—has inverted the traditional discovery funnel. The 90-fold improvements in hit rates and the successful in vivo validation of molecules like P076 against multidrug-resistant *A. baumannii* are not academic benchmarks; they are proof that AI can deliver novel, selective, and efficacious antibacterial candidates directly from the vast expanse of chemical space.

Crucially, the field has moved beyond black-box prediction. The integration of explainable AI through methods like SHAP is demystifying the molecular logic of activity, allowing researchers to understand and rationally optimize the features that govern antimicrobial action. Concurrently, sophisticated dereplication platforms such as GNPS and SIRIUS, augmented by transformer-based models like DreaMS and contrastive learning frameworks like CSU-MS^2^, are resolving the historic bottleneck of rediscovery. By achieving annotation rates of up to 90% and structure prediction accuracies exceeding 75%, these tools are ensuring that discovery pipelines are focused on true novelty. The ultimate power lies in the synergy of these approaches: metabologenomics workflows that seamlessly link a BGC to its expressed metabolite, and multi-omics strategies that connect microbial genomics to chemical phenotypes, are forging an end-to-end, data-driven highway from environmental sample to lead compound.

Looking forward, the full realization of AI’s potential in antibacterial discovery hinges on addressing several frontiers. First, the development of foundation models pre-trained on vast, unlabeled genomic and chemical datasets promises to unlock even greater predictive power for rare and novel biosynthetic pathways. Second, the systematic generation and integration of in vivo pharmacokinetic and pharmacodynamic data into predictive models will be critical to bridge the current gap between in vitro potency and clinical efficacy. Finally, fostering a culture of open data and model sharing will be essential to democratize these powerful tools and accelerate the global response to AMR.

Despite the transformative potential highlighted throughout this review, it is important to acknowledge several well-documented limitations rather than purely optimistic interpretations of AI-driven drug discovery. First, model reproducibility remains a significant concern, as many AI tools are not accompanied by publicly available code, trained models, or standardized benchmarking datasets, making direct comparisons between studies difficult. Second, publication bias toward positive results where studies reporting high prediction accuracy or successful experimental validation are more likely to be published than those reporting null or negative findings; may overstate the true performance of AI methodologies in real-world applications. Third, the quality and curation of training data vary considerably across studies; issues such as inconsistent bioactivity annotation, incomplete reporting of experimental conditions, and the presence of duplicate or erroneous entries in public databases can propagate biases into learned models. Recognizing these limitations does not diminish the substantial progress AI has enabled but rather underscores the need for community standards in model sharing, benchmark development, and data curation to ensure that future advances are both reproducible and generalizable across the antibacterial discovery pipeline.

In conclusion, the narrative of antibiotic discovery is being rewritten. By integrating AI across the entire discovery continuum—from mining silent genomes and predicting complex structures to optimizing lead candidates and validating their efficacy—we are no longer simply accelerating an old process; we are inventing a new one. The tools to explore the uncharted chemical darkness, to learn the language of metabolism, and to rationally design the next generation of antibiotics are now at our disposal. The fight against AMR has entered a new epoch, one where human ingenuity, amplified by machine intelligence, is finally equipped to match the evolutionary prowess of the microbial world.

## Figures and Tables

**Table 1:** Overview of global and regional burden of AMR.

Region/Country	Mortality Burden	Resistance Rates	Economic Impact	Key Pathogens	Ref.
Global	4.95 million deaths associated with AMR annually 1.27 million deaths directly attributable to resistant infections 65% increase in resistant infections (2000 to 2023)	Hospital-associated drug-resistant infections: 136 million cases annually	$693 billion in hospital costs (2019) $194 billion in productivity losses Projected $300 billion by 2030 Cumulative $100 trillion at risk by 2050	Multi-drug-resistant organisms ESKAPE pathogens	[1,2,3,4]
China	27.45% of inpatient infections resistant 15.77% exhibit MDR	MRSA: 32.2% 3GCREC: 54.2% CRAB: 56.1%	$77 billion total (0.37% of gross domestic product) $57 billion from MDR infections Excess cost per MDR patient: $3391	*A. baumannii* *E. coli* *S. aureus*	[3,5,6]
India	9 million hospital-associated infections annually	ESBL-producing Enterobacterales: 40–60%	$2.5 billion annually	*K. pneumonia* *E. coli*	[3,5]
Pakistan	10 million HAIs annually	High resistance to third-gen cephalosporins	PKR 361.9 million annually	Multiple MDR organisms	[3,5]
Thailand	28 deaths per 100,000 population	*A. baumannii*: 22.4 deaths/100,000 *S. aureus*: 4.1 deaths/100,000	$0.5 billion total MRSA alone: $180 million	*A. baumannii* *S. aureus* *E. coli*	[7]
Europe	541,000 associated deaths 133,000 attributable deaths	*E. coli*: 153,982 associated deaths	€1.1 to 1.5 billion annually €1.5 billion healthcare costs	*E. coli* MRSA *K. pneumoniae*	[1,6,8]
Greece	2100 deaths annually 580 Disability-Adjusted Life Years per 100,000	Meropenem-resistant *K. pneumoniae*: 75% Meropenem-resistant *Acinetobacter* spp.: 98% Colistin-resistant *K. pneumoniae*: 40% Colistin-resistant *Acinetobacter* spp.: 47%	€73 million hospitalization costs annually €13.9 billion projected over 10 years	*K. pneumoniae* *Acinetobacter* spp.	[8]
Germany	Significant increase in VREfm bloodstream infections (odds ratio = 1.18)	3GC-resistant: 5 to 25% *P. aeruginosa* carbapenem-resistant: 17.0% *P. aeruginosa* fluoroquinolone-resistant: 24.9% VREfm: 34.9%	Substantial healthcare burden	*P. aeruginosa* *E. faecium* Enterobacteriaceae	[9]
Russia	*E. coli* resistant: 8551 attributable deaths; 31,977 associated deaths	Aminopenicillin-resistant *E. coli* predominant	Significant economic burden	*E. coli*	[1]
Lebanon	HAI predominance for resistant organisms	CRAB: 84% HAI vs. 16% CAI CRPA: 79% HAI vs. 21% CAI MRSA: 65% HAI vs. 35% CAI ESBL-E: 55% HAI vs. 45% CAI	Excess charges: $1807 (HAI) Excess charges: $889 (CAI)	*A. baumannii* *P. aeruginosa* Enterobacteriaceae	[10]
Africa (Regional)	MDR bacteria cause 45% of deaths	MRSA: Cameroon (72%), South Africa (52%), Ethiopia (42.8%) >50% 3GC-resistant and fluoroquinolone-resistant in *E. coli* >50% carbapenem-resistant in *K. pneumoniae*	Country variation: <$250 (Senegal) to >$35,000 (Turkey)	*K. pneumoniae* *E. coli* *S. aureus*	[11]
South Africa	Nosocomial Enterobacteriaceae: 57.1%	MDR infections: 15.4%	Substantial healthcare costs	Enterobacteriaceae	[11]
United States	4.6 deaths per 100,000 population *S. aureus*: 3.5 deaths/100,000	Resistance rates comparatively lower	$55 billion excessive healthcare costs $35 billion annual cost $2.9 billion for 5 key pathogens	*S. aureus* *K. pneumoniae* *E. coli*	[5,6,7]
High-income countries	Lower mortality rates	15 million hospital-associated infections annually	Better infection control infrastructure	Variable by region	[3]
Developing countries	Resistance rates 3 to 4× higher than high-income nations	119 million hospital-associated infections annually	Disproportionate economic burden	All major pathogens	[2,3]
	Limited surveillance data	2 million hospital-associated infections annually	Limited healthcare resources	Limited data	[3]

**Table 2:** Molecular mechanisms and class-specific limitations of antibacterial resistance.

Resistance Mechanism	Description	Antibiotic Classes Affected	Key Genes/Enzymes	Clinical Examples/Prevalence	Ref.
Enzymatic degradation and modification: bacteria produce enzymes that chemically modify or degrade antibiotics, preventing target binding.					
β-Lactamases	Hydrolysis of β-lactam ring	Penicillins, cephalosporins	*blaZ*	Primary resistance mechanism in *S. aureus*	[15,16]
ESBLs	Hydrolyze third-gen cephalosporins and monobactams	Third-gen cephalosporins, monobactams	CTX-M, TEM, SHV variants	Global prevalence: 40 to 60% in Enterobacterales	[17]
Carbapenemases	Hydrolyze carbapenem antibiotics	Carbapenems, all β-lactams	KPC, NDM, OXA, IMP, VIM	Greece: *K. pneumoniae* 75%, Acinetobacter spp. 98% China: CRAB 56.1% Italy, Egypt: >25% in *K. pneumoniae*	[8,17,18]
Aminoglycoside-modifying enzymes	Acetylation, phosphorylation, adenylation	Aminoglycosides	aph(3′)-IIIa, aac(6′)-aph(2″), ant(6)-Ia	High-level resistance in Gram-positive and Gram-negative bacteria	[15]
16S rRNA methyltransferases	Methylation of ribosomal target site	Aminoglycosides	ArmA, RmtA-E	High-level pan-aminoglycoside resistance	[17]
Macrolide-modifying enzymes	Methylation of 23S rRNA	Macrolides, lincosamides	*erm*(B), *erm*(C)	Target site modification prevents antibiotic binding	[15,19]
Tetracycline resistance determinants	Ribosomal protection or efflux	Tetracyclines	*tet*(M), *tet*(K), *tet*(L), *tet*(O)	Widespread in Gram-positive and Gram-negative bacteria	[15]
Target site modification: alteration in antibiotic binding sites while maintaining essential bacterial functions					
PBP alterations	Altered PBPs with reduced β-lactam affinity	All β-lactams	*mecA* (PBP2a)	MRSA: 32.2% (China) US military: 9.3% (2005) to 16.7% (2014) Historical mortality: 82% (1940s), 98% (>50 years)	[6,16]
Fluoroquinolone resistance mutations	Mutations in DNA gyrase and topoisomerase IV	Fluoroquinolones	*gyrA*, *gyrB*, *parC*, *parE*	Germany: *P. aeruginosa* 24.9% fluoroquinolone-resistant >50% of *E. coli* in 5/6 WHO regions Clinical case: Ciprofloxacin failure with S79F *parC* mutation + efflux	[9,11,20]
Glycopeptide resistance	Remodeling of cell wall precursors (D-Ala-D-Ala to D-Ala-D-Lac or D-Ala-D-Ser)	Glycopeptides (vancomycin, teicoplanin)	*vanA*, *vanB*, *vanC*	Germany: VREfm 34.9% in bloodstream infections (odds ratio = 1.18) 1000-fold reduced vancomycin binding	[9,15]
Lipopeptide resistance	Cell envelope modifications	Daptomycin	Multiple regulatory systems	Emerging in *S. aureus* and enterococci	[19]
Rifampicin resistance	Mutations in RNA polymerase	Rifamycins	*rpoB*	Critical in MDR-TB	[16]
Oxazolidinone resistance	23S rRNA mutations or methyltransferases	Linezolid, tedizolid	*cfr*, 23S rRNA mutations	Saudi Arabia: 2.4% among MRSA isolates Emerging in Gram-positive bacteria	[17,19]
Efflux pumps and reduced permeability: decreased intracellular antibiotic concentration.					
Multidrug efflux pumps	Active export of multiple antibiotic classes	Tetracyclines, fluoroquinolones, β-lactams, aminoglycosides	RND family (MexAB-OprM, AcrAB-TolC) MFS family SMR family *qacA/B* (chlorhexidine)	Synergistic with other mechanisms Plasmid-mediated dissemination Contributes to MDR phenotypes	[15,16,19,20]
Reduced outer membrane permeability	Porin loss or modification	Hydrophilic antibiotics (β-lactams, tetracyclines)	*ompF*, *ompC*, ompK35/36	Particularly relevant in Gram-negative bacteria Acts synergistically with β-lactamases	[13,14,19]
Metabolic bypass: alternative pathways circumvent inhibited steps.					
Alternative metabolic enzymes	Acquisition of resistant metabolic variants	Sulfonamides, trimethoprim	*sul1*, *sul2*, *dfr* genes	Maintains bacterial viability while circumventing antibiotic action	[13,14]

**Table 3:** Overview of bottlenecks in traditional NPD.

Category	Specific Challenge	Quantitative Data/Statistics	Key Examples/Case Studies	Impact on Discovery	Ref.
Dereplication challenges	Rediscovery of known compounds	Only 1% of ~150,000 cataloged natural products investigated for bioactivity 95 to 98% of LC-MS features cannot be confidently identified New antibiotic-producing actinomycetes: 10^−7^ per random isolate	Extensive screening of >10 million microbes identified >2000 antibiotics Pfizer screened ~20 million tests from 135,000 soil samples for tetracyclines	>50% reduction in new chemical entity approvals Wasted resources on known compounds Declining discovery efficiency	[21,22,23,24]
	Limitations of current dereplication methods	Annotation rate: only 2 to 5% for LC-MS-based untargeted metabolomics CSI:FingerID: 150% more accurate identifications than second-best SIRIUS 4: >70% identification on challenging datasets	GNPS dataset of 3868 compounds used for benchmarking 5.4-fold more unique identifications with advanced tools	30% or more compounds remain unidentified Performance lower for truly novel scaffolds Resource-intensive manual validation	[23]
Low hit rates in screening programs	Quantitative hit rate data	Natural products: 0.3% hit rate (20 drugs from 7000 compounds) Synthetic libraries: <0.001% hit rate 300-fold advantage for natural products NCI program: 0.9% hit rate (3000 active from 326,656 fractions)	NCI program for NPD: 1,000,000 fractions from 125,000 extracts Large-scale antimicrobial screening: 326,656 fractions tested Biofilm inhibitor screen: 5.6% hit rate (165/2960)	99.1% of fractions show no activity Massive screening infrastructure required Low probability of success	[22,23,25,26]
	Hit-to-lead conversion challenges	10 to 20 years from discovery to market approval Thousands of compounds screened per approved drug Hundreds evaluated in preclinical/clinical studies	Multiple attrition points: potency, pharmacokinetics, toxicity, resistance High failure rate post-hit identification	Extended timelines High development costs Uncertain return on investment	[22]
Difficulty accessing unculturable organisms	Scale of unculturable diversity	99.9% of microorganisms unculturable using current techniques Only 0.1% cultivable in any given environment	Soil and marine environments: vast ‘white space’ of biochemical diversity ‘Biological dark matter’ of uncultured microbes	99.9% of microbial diversity inaccessible Limited to fast-growing, nutrient-generalist species Extensive screening of readily culturable organisms led to rediscovery	[27,28,29]
	Metagenomic approaches and limitations	Requires 10 to 20× genome coverage for reliable assembly Cosmid/fosmid: ~50 kb inserts Bacterial artificial chromosome: ~150 kb inserts Many BGCs: 50 to 100+ kb	10 million cosmid library screened for omnipeptin discovery Fungal artificial chromosomes-MS platform: 17 compounds from 3 *Aspergillus* species, including 15 novels	Size limitations prevent capture of complete BGCs Resource-intensive screening Success remains exceptional	[28]
	Alternative cultivation methods	Low cultivation success despite optimization	Marine microbes: combination methods showed modest improvement Requires substantial optimization and resource investment	Marginal expansion of cultivable fraction Not scalable for high-throughput	[30]
Silent BGCs	Prevalence of silent BGCs	>50% of BGCs in *Salinispora* expressed at detectable levels (implying ~50% silent) *A. nidulans*: produces 32 PKS, 14 NRPS, 2 indole alkaloids; >50% unidentified 3080 actinobacterial genomes: ~18,000 distinct gene cluster families, vast majority with no known products	*S. coelicolor*: fifth antibiotic (coelimycin P1) discovered after genome sequencing Model organisms still yield new compounds decades later	Cryptic biosynthetic potential inaccessible Even extensively studied organisms harbor silent clusters Massive untapped chemical diversity	[21,27,31]
	OSMAC approach	Variable success, empirical approach	*A. ochraceus*: 15 additional metabolites under different conditions *Streptomyces* sp. C34: 3 new macrolactone polyketides *P. quadriseptata*: 6 new metabolites by changing water source	Labor-intensive Unpredictable No guarantee of activation	[21]
	Co-cultivation	36.6% of actinomycetes produced unknown compounds in co-culture	*Gluconobacter* sp. + *N. crassa* or *A. oryzae*: produced enacycloxin Requires screening multiple organism combinations	Empirical approach Moderate success rate Labor-intensive	[21,32]
	Ribosome engineering	6% of non-Streptomyces actinomycetes acquired antibacterial activity 43% of *Streptomyces* species acquired activity after *rpsL* or *rpoB* mutations	*S. lividans* with *rpsL* mutations: produced actinorhodin from silent BGC Streptomycin-resistant mutants show activation	57 to 94% of strains show no activation Variable success across taxa Requires mutant generation	[21,32]
	Regulatory gene manipulation	Success in specific cases	*S. coelicolor*: *scbR2* deletion led to novel antibacterial activity *A. nidulans*: *hda* disruption activated sterigmatocystin, penicillin, and aspyridones	Requires genetic tools Target identification challenges Not universally applicable	[32]
	High-throughput elicitor screening	Identified antibiotics as major elicitors	*B. thailandensis*: 2 cryptic clusters activated Trimethoprim: global activator inducing ≥5 pathways Most elicitors were clinically used antibiotics	Requires screening infrastructure Elicitor specificity limitations Scalability challenges	[31]
	Heterologous expression	Cost per compound: up to $50,000+ Exonuclease Combined with RecET recombination + CRISPR/Cas9: cloned 106 kb salinomycin cluster Direct Pathway Cloning: ≤22 kb per round	Successfully expressed PKS and NRPS clusters Fontizine A5 and sodorifen discovered	Economically prohibitive for large-scale screening Technical challenges with large BGCs Host compatibility issues	[21,28]
Time, cost, and complexity constraints	Time requirements	10 to 20 years from discovery to market approval Marine natural products: 17 to 24 years (average 23 years) Isolation: 2 weeks Structure elucidation: 2 weeks (>90% of new compounds)	Extended timelines increase risk Scientific priorities shift over decades Market conditions change	Protracted return on investment High opportunity cost Discourages investment	[22,23]
	Cost implications	2004: $802 million per drug Current: >$1 billion to $4 to 11 billion BGC refactoring: $50,000+ per compound Screening infrastructure: millions in capital investment	NCI program: 326,000 fractions in 384-well plates Pfizer historical: 20 million tests from 135,000 samples 10^−7^ frequency = millions of isolates per novel producer	Prohibitive costs for academia Pharma divestment from NPD Economic barriers to entry	[21,22,23,24]
	Process complexity	Multi-disciplinary integration required: microbiology, chemistry, molecular biology, pharmacology, computational biology	Empirical screening dominates Limited rational prediction Gap between genomic potential and chemical diversity	Coordination challenges Multiple failure points High project risk	[27]
	Integration challenges	Genomic potential → chemical diversity gap remains substantial	Most predicted BGCs have no known products Limited ability to predict optimal expression conditions	Inefficient resource allocation Serendipity-driven discovery Suboptimal outcomes	[27]

## Data Availability

No new data were created or analyzed in this study. Data sharing is not applicable to this article.

## Abbreviations

The following abbreviations are used in this manuscript:

3GC	Third-generation cephalosporin
3GCREC	Third-generation cephalosporin-resistant *Escherichia coli*
AI	Artificial intelligence
AMP	Antimicrobial peptide
AMR	Antimicrobial resistance
ANN	Artificial Neural Network
antiSMASH	antibiotics & Secondary Metabolite Analysis Shell
AUC	Area under the curve
AUROC	Area under receiver operating characteristic
BGCs	Biosynthetic gene clusters
CAIs	Community-associated infections
CGRP	Calcitonin gene-related peptide
CI	Confidence interval
CNNs	Convolutional neural networks
COCONUT	COlleCtion of Open Natural prodUcTs
CRAB	Carbapenem-resistant *Acinetobacter baumannii*
CRPA	Carbapenem-resistant *Pseudomonas aeruginosa*
CSU-MS^2^	Cross-Modal Compound Identification from MS/MS Spectra to Molecular Structures
DiscERN	Discoverer of Evolutionarily Related Natural products
DL	Deep learning
D-MPNN	Directed-message-passing neural network
DreaMS	Deep Representations Empowering the Annotation of Mass Spectra
EEA	European Economic Area
ESBL	Extended-spectrum beta-lactamase
ESM	Evolutionary Scale Modeling
EU	European Union
FBMN	Feature-Based Molecular Networking
FIDDLE	Formula IDentification by Deep LEarning
GANs	Generative adversarial networks
GCNs	Graph Convolutional Networks
GeMS	GNPS Experimental Mass Spectra
GNNs	Graph neural networks
GNPS	Global Natural Products Social Molecular Networking
HAIs	Hospital-acquired infections
HILIC	Hydrophilic interaction liquid chromatography
iDCNNPred	Interpretable deep convolutional neural network prediction
IFPTML	Information Fusion Perturbation-Theory Machine Learning
IR	Infrared
JESTR	Joint Embedding Space Technique for Ranking Candidate Molecules
Kd	Dissociation constant
KNN	K-nearest neighbor
LC-MS	Liquid chromatography-mass spectrometry
LSTM	Long Short-Term Memory
MAW	Metabolome Annotation Workflow
MCC	Matthews correlation coefficient
MDR	Multi-drug resistance
MHSDPA	Multi-head scaled dot-product attention
MIC	Minimum inhibitory concentration
MIST	Metabolite Inference with Spectrum Transformers
ML	Machine learning
MolE	Molecular representation through redundancy reduced Embedding
MRSA	Methicillin-resistant *Staphylococcus aureus*
MS	Mass spectrometry
MTL	Multitask learning
NCI	National Cancer Institute
NLP	Natural language processing
NMR	Nuclear magnetic resonance
NPD	Natural product discovery
NPOmix	Natural Products Mixed Omics
NRPS	Non-ribosomal peptide synthetases
OSMAC	One Strain Many Compounds
PBP	Penicillin-binding protein
PCC	Pearson’s correlation coefficient
PECAN	Predicting Patterns of Cancer Cell Cytostatic Activity of Natural Products
PKS	Polyketide synthases
RiPPs	Ribosomally Synthesized and Post-translationally Modified Peptides
RMSE	Root mean squared error
QSAR	Quantitative Structure-Activity Relationships
RF	Random forest
RL	Reinforcement learning
SAR	Structure–activity relationship
SSFDB	Spectrum-searchable Structural Feature Database
SVMs	Support vector machines
UV	Ultraviolet
VREfm	Vancomycin resistance in *Enterococcus faecium*
WHO	World Health Organization
XOD	Xanthine oxidase
